# Preimplantation development regulatory pathway construction through a text-mining approach

**DOI:** 10.1186/1471-2164-12-S4-S3

**Published:** 2011-12-22

**Authors:** Elisa Donnard, Adriano Barbosa-Silva, Rafael LM Guedes, Gabriel R Fernandes, Henrique Velloso, Matthew J Kohn, Miguel A Andrade-Navarro, J Miguel Ortega

**Affiliations:** 1 Laboratório Biodados, Dept. de Bioquímica e Imunologia, Universidade Federal de Minas Gerais, 31270-901, Belo Horizonte - MG, Brazil; 2 Departamento de Bioquímica, Universidade de São Paulo - SP, Brazil; 3 Computational Biology and Data Mining Group, Max-Delbrück Center for Molecular Medicine, Robert-Rössle-Strasse 10, D-13125, Berlin, Germany; 4 Bioinformatics Graduate Program, Federal University of Paraná - UFPR (SEPT). Rua Dr. Alcides Vieira Arcoverde 1225, CEP 81520-260. Curitiba-PR, Brazil; 5 New York State Stem Cell Science, New York State Department of Health Wadsworth Center, Rm C345, New York, USA

## Abstract

**Background:**

The integration of sequencing and gene interaction data and subsequent generation of pathways and networks contained in databases such as KEGG Pathway is essential for the comprehension of complex biological processes. We noticed the absence of a chart or pathway describing the well-studied preimplantation development stages; furthermore, not all genes involved in the process have entries in KEGG Orthology, important information for knowledge application with relation to other organisms.

**Results:**

In this work we sought to develop the regulatory pathway for the preimplantation development stage using text-mining tools such as Medline Ranker and PESCADOR to reveal biointeractions among the genes involved in this process. The genes present in the resulting pathway were also used as seeds for software developed by our group called SeedServer to create clusters of homologous genes. These homologues allowed the determination of the last common ancestor for each gene and revealed that the preimplantation development pathway consists of a conserved ancient core of genes with the addition of modern elements.

**Conclusions:**

The generation of regulatory pathways through text-mining tools allows the integration of data generated by several studies for a more complete visualization of complex biological processes. Using the genes in this pathway as “seeds” for the generation of clusters of homologues, the pathway can be visualized for other organisms. The clustering of homologous genes together with determination of the ancestry leads to a better understanding of the evolution of such process.

## Background

Bioinformatics tools currently allow research to focus on the integration of large-scale data generated by sequencing, differential expression analysis, gene interaction studies and others. Several initiatives exist to organize this knowledge in secondary databases, thus allowing easier access and visualization. Databases containing interaction information are a good source for novel research. iHOP [1] allows users to tag gene names of interest and browse through the related PubMed literature with highlighted keywords. Another interaction database is STRING [2], which contains physical interactions and functional associations between proteins and integrates data retrieved from literature (PubMed), genomic context, large scale experiments and conserved co-expression. Text-mining, therefore, has a fundamental role in these tools and allows access to interactions spread throughout the literature. The extraction of biological events from literature through text-mining tools is essential to not only update the interaction databases but also for the creation and annotation of pathways.

Metabolic and regulatory pathways are an example of organized knowledge that allow a better visualization of a complex system and can be found in databases such as iPath [3], BioCyc [4] or KEGG Pathways [5]. When orthology information is added to pathways, the same process can be represented in different organisms. Orthology is also an important tool for sequence annotation. Current orthologue databases such as COG and KOG [6], eggNOG [7], OrthoMCL [8] and KEGG Orthology [5] all provide a good source for manually curated clusters of orthologues defined for organisms with complete genomes. We developed a procedure to enrich the COG database with UniRef50 clusters from the UniProt database [9], creating the UECOG database [10]. Recently, a similar procedure was applied to the KEGG Orthology database creating the enriched UEKO database (unpublished, Fernandes *et al*.).

The available tools described raise the possibility of integrating current information and generating complex regulatory pathways. Previous publications individually reported the regulatory interactions that control preimplantation embryo development [11-15]. However, a complete preimplantation development regulatory pathway has never been built.

In humans, the preimplantation phase of embryonic development is a period of approximately six days after fertilization prior to attachment of the embryo to the uterine wall. Implantation can occur before or in the seventh embryonic day (E7), a time during which the uterus is receptive [16]. Mammalian embryonic development has been thoroughly studied in mice and the blastomeres remain totipotent, able to generate any other cell, up to the eight-cell stage, unlike other animals [17]. After fertilization, successive cleavages take place during the first two days of development, resulting in the eight-cell embryo. The next stage of development is called the morula stage. An increase in cell-cell contact results in formation of a compacted morula. The subsequent divisions increase the complexity of the embryo and cells may be located on the inside, surrounded by other cells, or on the outside, in contact with the environment. The identification of the initial cells for each lineage has shown that the trophectoderm (TE) is derived mostly from the outer cells, whereas the inner cells give rise to the inner cell mass (ICM). Later, the ICM divides into the primitive endoderm (PE) and the epiblast (EPI). During the differentiation of the TE from the ICM, the blastocoel is formed through a process of cavitation. The embryo is called a blastocyst when all three structures are present (TE, ICM and blastocoel). Twenty-four hours after blastocyst formation occurs, the last stage of preimplantation development takes place when the PE differentiates from the ICM. The three lineages thus formed in preimplantation development present different fates during subsequent embryonic development. While the epiblast, which forms from the ICM following implantation, is still undifferentiated and will give rise to the fetus itself, the trophectoderm will become the fetal portion of the placenta and the primitive endoderm (as part of the extraembryonic endoderm) will form the yolk sac [14]. Complex regulatory processes such as animal development are a result of the interaction of many different gene products and elements that control the expression of these genes. Traditional experiments that determine the function of one or a few genes are essential, but do not result in a comprehensive view of complex systems. A complex regulatory network should be able to portray specific and general aspects of development, such as the embryonic fate of certain cells [18].

In this work, we noticed the absence in databases of a pathway describing the preimplantation phase of embryo development and sought to develop the given pathway using text-mining tools, complementing it with orthology information. The resulting pathway comprises 86 genes and the interactions between them. Clusters of orthologous groups were generated for each gene represented in the pathway and provided the necessary information to determine the last common ancestor. This determination revealed that the preimplantation development pathway is an ancient Chordata pathway with addition of modern elements throughout evolution.

## Results

### Text-mining

Initially, we used the PubMed platform to search for articles related to the embryo preimplantation development (query: “preimplantation development”) and obtained 3524 entries as a result. To obtain a more efficient set of articles with relevancy to our work, the result entries were submitted to MedlineRanker [19]. This software computes discriminating words by comparing a set of user selected abstracts indicated as highly relevant to a background set and then scores any abstracts in terms of their content of those discriminating words. After the classification, we selected the top 1000 abstracts for further analysis, which presented p-value lower than 0.01 and by manual inspection provide large amount of information when uploaded in PESCADOR. Since human and mouse embryo development are highly similar, it was plausible to use abstracts from work on both organisms as source of information for the preimplantation pathway construction, paying attention to any possible conflict.

**Figure 1:**
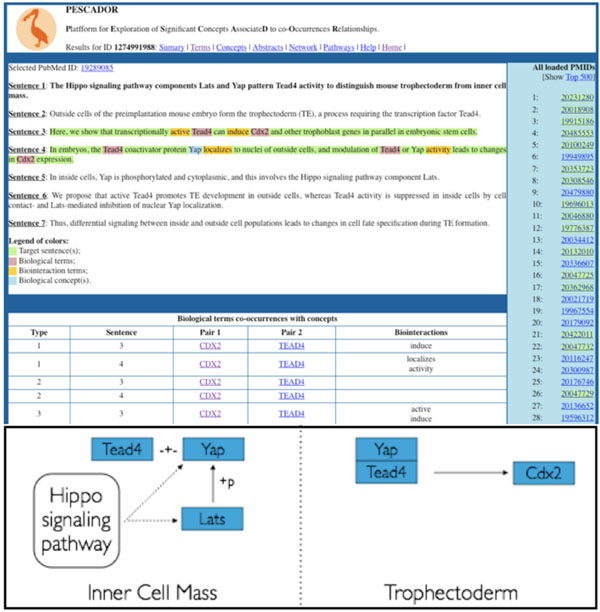
**Biointeraction extraction from PESCADOR.** Top: Sample abstract tagged by PESCADOR. Gene or protein names (terms) recognized are highlighted in violet and the biointeraction words in yellow. The platform allows users to search for their interactions of interest by terms, abstracts or concepts of interest added initially by the user. Bottom: Manual curation of the information presented in the abstract and its graphical representation in the form of a regulatory pathway.

**Figure 2:**
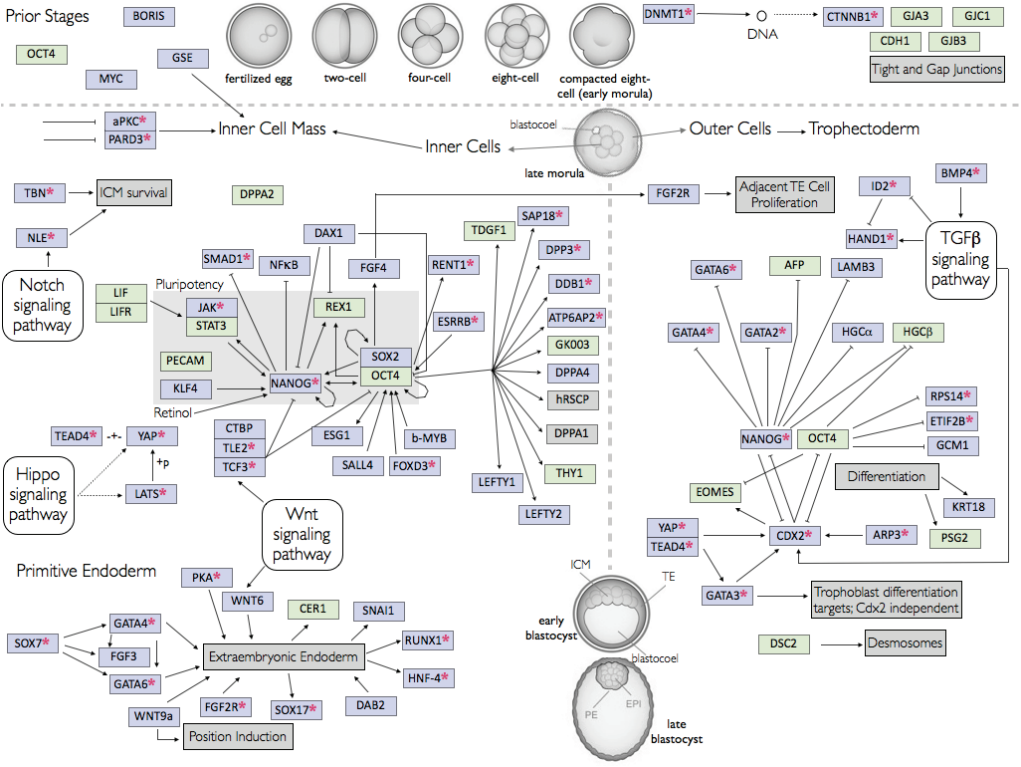
**Preimplantation development pathway.** The figure shows a pathway representation of the genes involved in the regulation of the preimplantation development and interactions between them. Some functions are also detailed in the grey rectangles. In the upper part of the figure are located genes involved in the early stages of development (until blastocyst formation) below these, the left part corresponds to the regulations that occur in the inner cell mass, the portion of cells that remains undifferentiated during a longer period of time, part of these cells will give rise to the primitive endoderm and the genes that regulate this process are shown in the bottom left. In the right are the genes involved in the development of the outer cells of the blastocyst, which differentiate to form the trophectoderm. The interactions are described in the text. KEGG Markup Language was used for pathway representation. The developmental stages figures were adapted from Yamanaka *et al.* 2006 [14]. The pathway genes are represented according to their ancestry based on the determination of their Last Common Ancestor. Genes considered recent are shown in green while genes of more ancient origin are shown in lilac. Genes that present an ortholog in *D. melanogaster* are marked (*). This will be further adressed in the text section “Pathway Ancestry”. DPPA1 and hRSCP are shown in grey due to the fact that the lack of corresponding SwissProt annotated gene product to be used as seed prevented their use in this analysis.

Using these 1000 highly informative abstracts as our input, PESCADOR (manuscript in preparation, Barbosa-Silva *et al*.) an online platform for friendly operation of the LAITOR software [20]) was used for tagging of gene names and biointeractions extraction from each abstract. As a result, 722 gene names were tagged and 223 type 1 biointeractions were highlighted as well as other informative biointeractions. Biointeractions are classified by LAITOR [20] as type 1 when in the same sentence the software encounters a gene name, a biointeraction word and another gene name, in that order (e.g.: CDX2 downregulates NANOG). From these tagged abstracts we manually curated the information and constructed the pathway for the preimplantation embryo development describing 86 genes and numerous interactions between them during the early developmental stages, trophectoderm differentiation from the inner cell mass and posterior extraembrionary endoderm differentiation. A sample abstract tagged by PESCADOR and the manual extraction of the information it contains is exemplified in Figure 1. The pathway shown in Figure 2 was constructed according to KGML (KEGG Markup Language). The large decrease in the initial number of genes tagged in the abstracts is mainly due to redundancy between abstracts (same genes mentioned) and also to genes tagged in type 3 and 4 biointeractions, which not always result in pathway building information.

### Preimplantation pathway

The pathway obtained after the analysis of all the abstracts from the PESCADOR output is represented in Figure 2 and the regulations are reviewed below.

#### First embryonic cleavages

The oncogene c-MYC is an important transcriptional regulator and its expression is observed in the initial stages of development, where it is present in embryonic cells until the morula stage and repressed thereafter [21]. Two additional genes recently associated with these early developmental stages are BORIS and ECSA. BORIS is involved in early development following fertilization and soon afterwards repressed, and ECSA, expression begins in the blastocyst exclusively in the cells of the inner cell mass (ICM). The presence of these genes was compared to the expression pattern of the Oct4 transcription factor, which is present in the early cleavages, repressed after this initial stage, and then its expression is afterwards stimulated again in the blastocyst [22]. The expression of the gametogenesis associated gene Gse was also recently identified in cells of the early embryo; later this protein is found only in the ICM, suggesting a role in the specification of cell lineage [23].

#### Methylation patterns and correct preimplantation development

Genomic methylation patterns in mammalian cells depend on Dnmt1 (DNA methyltransferase-1). In the mouse, an embryo-specific variant called Dnmt1o is expressed in the early stages of development. In the 8-cell stage this protein relocates to the cell nucleus where maintains essential methylation patterns, allowing embryos to complete early developmental events [24]. It was recently shown [25] that the inability of Dnmt1o to properly relocate not only results in a developmental arrest at the 5-7 cell stage, but is also responsible for the downregulation of five genes involved in the formation of gap and tight junctions (Cx31, Cx43, Cx45, Cdh1 and Ctnnb1). These junctions are crucial for early processes such as compaction of the 8-cell embryo and cavitation of the blastocoel.

#### TE versus ICM dichotomy: key role of Lats controlling Tead4 co-activator Yap

Cells destined to become part of the ICM are marked by repression of two genes (aPKC and PARD3) [26] and by upregulation of Sox2 [11]. In these cells, the major pluripotency transcription factors, including Nanog and Oct4, remain active due to the expression of an important player and member of the Hippo signaling pathway: Lats. This serine/threonine protein kinase is responsible for phosphorylating Yap, leading to its cytoplasmic localization and thus preventing its association with the transcription factor Tead4.

#### Triggering TE differentiation: Tead4/Yap target Cdx2 to repress Nanog and Oct4

Conversely, in the outer cells that will differentiate and form the trophectoderm, Yap is unphosphorylated, remains in the nucleus and associates with Tead4, leading to the activation of Cdx2, a key repressor of Nanog and Oct4 [27]. Repression of Oct4 and Nanog transcription by Cdx2 then releases the inhibition that these two key factors were exerting on many different genes, in turn activating these targets [28,29]. Activation of Cdx2 requires release from basal repression; Nanog [30] and Oct4 [31] repress basal levels of Cdx2 and induction of higher levels of Cdx2 by Tead4/Yap overcomes this repression, allowing Cdx2 to play its role [28]. Tead4 was also recently determined to activate another trophectoderm differentiation factor, GATA3 [32], which acts alongside Cdx2 and affects transcription of a number of genes independent of Cdx2. The Tead4-dependent activation of GATA3 seems to be independent of Yap, suggesting Tead4 interacts with another partner as well as Yap. Also required for high level expression of Cdx2 in trophectoderm cells is the cell motility protein Arp3; experiments with complete knockdown of this protein show trophoblast cells unable to develop properly, possibly undergoing apoptosis as a result of loss of Cdx2 [33]. The TGFbeta pathway is another important pathway for trophectoderm differentiation; TGFbeta signaling is stimulated by BMP4, which leads to the activation of SMAD proteins. These proteins can also stimulate transcription of Cdx2 [34], and BMP4 is known to inhibit Id2, an inhibitor of differentiation [35], and to activate Hand1, which is involved in trophoblast cell differentiation [36].

#### In the absence of Oct4 and Nanog

The downregulation of Oct4 in the outer cells of the embryo leads to the activation of a positive regulator of TE cell fate, Eomes (T-box protein eomesodermin) [29,37], which is also a possible Cdx2 target [38]. The subsequent differentiation of these cells into trophectoderm is accompanied by the expression of several genes, such as the glycoprotein PSG2 [39] and the marker KRT18. PSG2 and KRT18 expression are among the first signs that a blastomere has lost its totipotent competence, prior to any visible differentiation [33]. Removal of Oct4-dependent repression also results in activation of genes such as ETIF2B and Rps14 [40], allowing these cells to engage in an intense translation routine. Knockdown studies targeting Oct4 also show that it represses the expression of Gcm1, which is normally placenta specific [41], and of the hCG hormone’s beta chain [42].

Concurrently, Nanog downregulation allows the expression of a number of genes associated with both trophectoderm (GATA2, hCG-alpha and hCG-beta) and extraembryonic endoderm (GATA4, GATA6, LAMB1 and AFP) [30]. These latter genes will in turn initiate the formation of tissues such as the primitive endoderm, a component of the yolk sac. From the early blastocyst stage on, desmosomes are assembled in the trophectoderm in response to desmocollin (DSC2), which is also not expressed in the ICM [43].

Thus, Tead4/Yap activation of Cdx2, accompanied by the subsequent repression of Nanog and Oct4, describes a scenario for the TE differentiation.

#### Underneath the maintained activation of Oct4 and Nanog

Back in the ICM, the main pluripotency genes remain active and form a complex regulation pathway. Recently it was discovered that transcription of Nanog is further stimulated by the presence of compounds such as retinol [44]. Klf2, Klf4 and Klf5 exert a redundant role in the activation of Nanog. These krüppel-like factors were described as essential for the maintenance of pluripotency. Indeed, Klf4 was already known for this role and is commonly used in reprogramming of differentiated cells into induced pluripotent stem cells. However, only the simultaneous depletion of Klf4, 2 and 5 results in the differentiation of stem cells, indicating functional redundancy [45]. Other proteins known to activate Nanog include the two other main pluripotency regulators, Oct4 [37,46] and Sox2 [47]. The estrogen receptor ESRRB is also reported to be involved in the activation of Nanog by Oct4 and Sox2 [47]. Conversely, Nanog can activate Oct4 [46], and ESRRB is necessary to maintain Oct4 promoter activity [48].

Each of the three key factors, Oct4, Sox2 and Nanog, also act as self-activators, e.g. the partners Oct4 and Sox2 bind and activate Oct4 transcription [49]. Another key transcription factor involved in the maintenance of cell pluripotency is Sall4 [50]. Sall4 binds to the conserved regulatory region in the Pou5f1 (the Oct4 gene) distal enhancer and activates its transcription [31]. Studies with microRNA interference of Sall4 show that the loss of this factor leads to reduction of Oct4 mRNA levels and significant expression of Cdx2 in the ICM [31]. b-MYB, a gene expressed in proliferating cells, is also a positive regulator of Oct4 and studies report early differentiation of ICM in the absence of b-MYB [51].

The Notch signaling pathway is a conserved pathway that is involved in cellular communication processes and correct cell fate decisions that also has a role in ICM development [52]. Nle protein, a direct regulator of this pathway, is essential for survival of the ICM [53]. Another protein associated with development and survival of the ICM is Tbn (Taube nuss), whose absence promotes cell apoptosis in the ICM [54].

Expression of the platelet and endothelial cell adhesion molecule (PECAM1 or CD31) was detected by immunofluorescence confocal microscopy in the blastocyst and restricted to the ICM cells. Subsequently, PECAM1 remains only in the pluripotent epiblast cells, disappearing the moment these cells undergo differentiation [55], and indicating a new role for this molecule during embryo development.

#### Activation, but with moderation

Other control pathways maintain expression of these genes at a steady-state concentration and balance these many mechanisms for activation and upregulation of transcription. A complex regulation feedback loop consists of FOXD3, Nanog and Oct4 [46]. To keep Oct4 and Nanog expression within steady-state levels, these three genes interact so that (i) expression of Nanog activates FOXD3 and Oct4 but not above steady-state levels due to Oct4 exerted repression; and (ii) FOXD3 and Nanog activate Oct4 expression but not above steady-state levels due to Oct4 self-repression.

Dax1 is an orphan nuclear hormone receptor recently identified as a repressor of Oct4 transcription [56].

Dax1 expression was also capable of reducing Nanog and Rex1 expression. Assays show that Dax1 binds to Oct4 and abolishes its DNA binding activity, thus decreasing the transcription of Nanog and Rex1, targets of Oct4 activation.

Another repressor in the ICM is Tcf3, a Wnt signaling pathway effector. TLE2 (a Groucho family protein) and CtBP (C-terminal binding protein) are key partners of Tcf3 in mediating this repressive effect. Tcf3 binds to and represses the Oct4 promoter, and this repressive effect requires both the Groucho and CtBP interacting domains of Tcf3 [13]. Tcf3 also limits the steady-state levels of Nanog mRNA, protein, and promoter activity in self-renewing embryonic stem cells (ESCs); the Tcf3 Groucho domain is involved in this repression [57]. Thus, Tcf3 is critical for maintaining the appropriate levels of both Oct4 and Nanog in ESCs. Experiments show that loss of Tcf3 by RNA interference (RNAi) knockdown blocks the ability of ESCs to differentiate [13], emphasizing the importance of this interaction.

#### Downstream of Oct4 and Sox2

Oct4 activates embryonic stem cell-specific gene 1 (Esg1), which encodes an RNA binding protein present in the ICM that is responsible for regulating several specific target transcripts [58]. Oct4 and Sox2 are also responsible for the regulation of the fibroblast growth factor 4 (FGF4) [49]. Expression of FGF4, therefore, requires the combined activity of these two transcription factors that bind to adjacent sites on the FGF4 enhancer DNA region [59]. Once expressed, the FGF4 protein can interact with its receptor FGFR2 and activate ICM and adjacent TE cell proliferation, activating extraembryonic endoderm cells as well in later stages.

Several other genes with important functions in embryonic development are also targets of Oct4-dependent activation. These include growth factor TDGF1, growth inhibitor SAP18, regulator of nonsense transcripts RENT1, two proteins involved in stem cell self-renewal DPPA4 and DPPA1 (developmental pluripotency associated), anterior visceral endoderm (AVE) markers LEFTY1 and LEFTY2, surface antigen THY1, and other genes encoding proteins involved in specialized cellular processes (DPP3, ATP6AP2, DDB1) and hypothetical proteins (GK003, hRscp) [37,40].

The master regulation exerted by Sox2 and Oct4 during mammalian embryogenesis is believed to operate through their cooperative binding to DNA regulatory regions composed of adjacent HMG and POU motifs (HMG/POU cassettes) [60]. Exemplifying this arrangement, DPPA4 is one such gene with the presence of an HMG/POU cassette in its promoter region [61].

#### Downstream of Nanog and STAT3

Activation of JAK/STAT pathway also has an important contribution to pluripotency. In mice, the LIF/STAT3 pathway [44,62,63] for maintenance of cell pluripotency comprises LIF and LIF receptor, which deliver intracellular signaling through STAT3. STAT3, a signal transducer and activator of transcription is activated by the JAK1 kinase and binds to several promoters inducing transcription of pluripotency related genes [64]. Nanog and Stat3 were found to bind to and synergistically activate Stat3-dependent promoters [64]. Nanog also functions as a transcriptional inhibitor to NFκB, a factor known to have pro-differentiation activity [64]. Nanog is also responsible for SMAD1 repression, thereby preventing BMP4-induced differentiation through the TGFbeta signaling pathway, for which SMAD1 is a key signal transducer [65].

#### Extraembryonic endoderm differentiation from ICM cells

Prior to embryo implantation one more differentiation takes place. Certain cells from the ICM give rise to the primitive endoderm, the first morphologically distinct cell type of the extraembryonic endoderm. The extraembryonic endoderm comprises the primitive, parietal and visceral endoderm components and will become the yolk sac during posterior development stages.

Wnt6 was recently identified as an inducer of primitive endoderm and this induction is accompanied by translocation of beta-catenin (CTNNB1) and Snail1 to the nucleus [66]. This study also showed that up-regulation of protein kinase A (PKA) induces markers of parietal endoderm. Another Wnt family member, Wnt9a, is expressed only in ICM cells that surround the bastocoel [67] and induces repositioning of the cells expressing GATA6, which is necessary for formation of primitive endoderm [68].

Sox7 plays a major role in parietal endoderm differentiation. Through studies with short interfering RNA molecules, it was established that Sox7 is responsible for transcription induction of GATA4 and GATA6 [69]. Individual or combined silencing of Sox7, GATA4 and GATA6 result in suppression of cell shape changes and production of laminin-1 (LAMB1), characteristic changes present in parietal endoderm differentiation [69]. Gata4 was previously identified as a transcription factor responsible for the activation of FGF3 [70]. Sox7 also activates the FGF3 promoter. Conversely, Sox2 can negatively modulate the GATA4-dependent activation of FGF3, which is supported by the role of this factor in ICM pluripotency [71]. Another Sox family member, Sox17, is responsible for the differentiation of the extraembryonic endoderm in the final steps of preimplantation development [72]. The Runx1 factor is associated with the expression of Sox17 and is also specific for the extraembryonic endoderm [73]. HNF4 is a transcription factor specific of the extraembryonic endoderm with subsequent roles in post-implantation development and organogenesis [74]. Its expression may result from BMP4-induced differentiation [75]. Finally, the Dab2 protein is indispensable for the development of visceral endoderm; though its exact role is still not established, it is perhaps related to correct cell positioning [76,77]. The expression of Cer1, a marker of the anterior visceral endoderm (AVE), commences before embryo implantation in the subset of cells that comprise the primitive endoderm. This ancestral population includes both cells expressing Cer1 together with cells in which Cer1 expression begins after implantation and formation of the AVE [60].

### Search for homologues

To establish an ortholog database and provide sequence information to the genes contained in the preimplantation pathway, aminoacid sequences corresponding to the human and mouse gene products were used as seed for the software SeedServer (Guedes *et al*., unpublished, see Methods for details). In fact, only the UniProt identifier for these proteins is necessary to execute SeedServer - gene symbols were verified in the NCBI Gene database and converted to the corresponding geneID, and the desired identifiers were obtained afterwards from the UniProt database. For each gene a cluster of homologues was generated comprising from 2 to 260 sequences (Additional file 1).

The recruited sequences contained in each cluster can be Swiss-Prot annotated or unrevised TrEMBL sequences. In total, 25% of the cluster sequences are Swiss-Prot, the great majority of clusters being comprised of TrEMBL sequences (75%). The search for homologues through SeedServer provides therefore a large amount of candidates for manual curation in Swiss-Prot. Furthermore, SeedServer can recruit sequences from organisms without a complete genome due to its use of UEKO (UEKO is built on top of Kegg Orthology homologues as UECOG [8] has been built on top of COG database) and bidirectional best hit (BBH) searches conducted by SeedLinkage [78], and in fact only 27% of the sequences present in all clusters are from organisms with a complete genome. The ortholog clustering by SeedServer was only performed for genes that had a corresponding SwissProt annotated gene product to be used as seed, therefore hRSCP and DPPA1, which are described in the pathway, did not go through this analysis.

### Pathway ancestry

We then focused on the putative origin of these genes, determining which clade in the human lineage (e.g. class, order, family) shares each gene. The generation of ortholog clusters allowed for the determination of the last common ancestor (LCA) for each of the genes in the pathway. Figure 2 shows the genes according to their origin. Genes were arbitrarily considered ancient for this analysis if their last common ancestor originated before the divergence of the clade Euteleostomi and are coloured grey. Genes with a LCA belonging to the clade Euteleostomi or originated after divergence of Euteleostomi are considered recent genes and are coloured blue. Ancient origin genes with an ortholog in *Drosophila melanogaster* are marked with a red asterisk. This arbitrary classification was meant to attract attention to the two key pluripotency controlling genes, Nanog (ancient) and Oct4 (modern).

**Figure 3:**
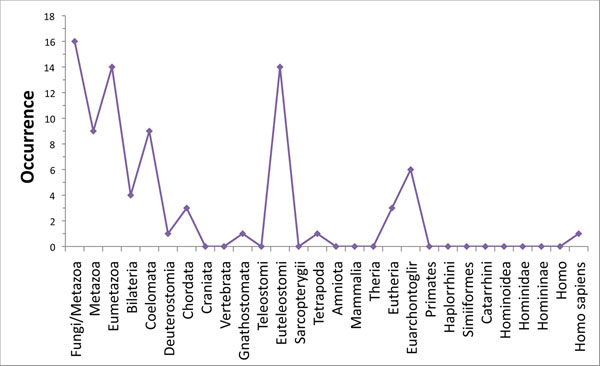
**Gene origin in human evolution.** Distribution of the genes in the preimplantation pathway according to their origin in clades of the human lineage, based on the determination of the Last Common Ancestor for the ortholog clusters generated by SeedServer. The y-axis represents the number of genes and the x-axis represents the taxonomical groups in which the genes originated.

The graph shown in Figure 3 represents the distribution of all the genes in the pathway according to their origin respect to clades of the human lineage. It may be observed that a large quantity of genes originates in certain periods as seen in Eumetazoa, Coelomata, Euteleostomi and Eutheria. The reasons for this wavelike origin need to be further analysed. On the other hand, the apparent origin of complex structures, that characterize all descendents from a certain moment of evolution, might have occurred simultaneously to the specialization of gene groups. The coverage of genomic sequences in the database is far from homogeneous and can influence the shape of this graph [79]. In any case, the pattern observed agrees with the expansion of protein families related to stem cell markers observed in the ray-finned fish, that is, after divergence of the Euteleostomi [80].

Furthermore, we searched for functional information related to the *D. melanogaster* orthologues in order to determine if these functions are somehow similar or related to the functions of the corresponding pathway genes. This was done through a second text mining approach similar to the first and from the information recovered a secondary pathway was generated simply to illustrate the ortholog genes and their relative functional roles (Additional file 2). The regulatory pathways in which these genes are involved show us that these genes are all related to some part of *Drosophila* embryo development, some of them with highly conserved functions still observed in the preimplantation pathway described. An example is the Hippo signalling pathway, which is extremely conserved, showing Wts (Lats ortholog) phosphorylating Yki (Yap ortholog); this modification prevents Yki interaction with Sd (Tead4 ortholog). The correlation between the human gene names and corresponding *D. melanogaster* ortholog names can be found in Additional file 3 and also the PMID reference for the gene function in Drosophila development.

## Discussion

The use of text-mining tools for the generation of regulatory pathways is an effective approach and it is important for the current interest of gathering data related to an organism or biological process. The search for information related to a specific concept such as “preimplantation development” resulted in the selection of data related to this process only. When other tools such as iHOP [1] and STRING [2] are used for the search of biointeractions, it is necessary to know the names for the genes you are interested on and the information is then retrieved. Moreover in the case of iHOP, the information retrieved consists of a large list of papers related to the gene of interest, which need to be manually analysed to extract the information related to the specific process. In the case of STRING, the result of a query is a network of direct associations to other genes, which can be activations, repressions, or unknown, but for which it is not possible to perform a search restricting the query to a specific process for which you seek to determine the involvement of a given gene.

**Figure 4:**
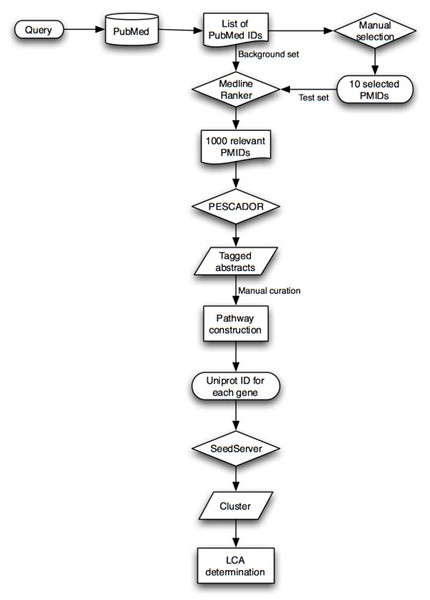
**Pathway construction flowchart.** The initial step consists of a PubMed search with the subject of interest (e.g. preimplantation development). The list of PubMed identifiers (PMIDs) obtained in the search is then used in the web tool Medline Ranker as the background set along with a list of PMIDs of manually selected abstracts considered informative which form the test set. The tool generates a list of abstracts classified by order of relevance. Best 1000 abstracts are recovered and their corresponding PMID is then introduced in the PESCADOR platform. Abstracts are tagged by PESCADOR and provide a source of biointeractions for manual curation and pathway construction. UniProt IDs for products of the genes present in the final pathway are obtained and used as seed in SeedServer. The software recruits homologues for each gene and creates the final clusters. Taxonomy IDs from each cluster can be used for Last Common Ancestor (LCA) determination.

The approach described in this work (using PubMed, MedlineRanker, PESCADOR) summarized in Figure 4, allows the researcher to initiate the study of a pathway without knowing exactly the genes involved, simply by selecting the published information related to the process of interest. The manual curation required to create a pathway through this approach is significantly smaller. However, the verification of all the interactions highlighted by the tool is essential. Text-mining is not able to eliminate the selection of false interaction pairs; in the case of LAITOR (contained in the PESCADOR platform), the type 3 and 4 interactions can present genes with no association specified in the text [20].

The text-mining data contribute the complete description of the pathway in the form of a literature review, a necessary step for the validation of the regulations represented, and for the inclusion of the pathway in a specific database, such as KEGG Pathway. The establishment of this procedure for pathway generation allows future work to enlarge the knowledge on subjects still not approached, such as regulatory pathways for several types of cancer, mechanisms of pathogen resistance in plants and response to abiotic stresses in plants, among other themes of interest.

The inclusion of the preimplantation pathway in databases such as the KEGG database will allow automatic annotation for several other organisms, as it is usually done in this database. Concurrently, a laboratory with a specific interest can promptly build a similar Pathway for its local use. From the 86 genes present in the pathway, 20 do not possess entries in KEGG Orthology and would constitute important additions. Considering that the contribution of KEGG for the sequence recruitment in the SeedServer clusters is only 25% of the total number of sequences, some organisms evolutionarily divergent from the ones represented in KEGG begin to play a more relevant role for a more efficient annotation of new sequences. It is relevant to stress that only the SeedLinkage and UEKO components of SeedServer are capable of clustering sequences proceeding from organisms without a complete genome project. Moreover, linkage of recruited to seed sequences are verified with PSI-BLAST.

Another important contribution from the ortholog clustering by SeedServer is the identification of candidates for Swiss-Prot Annotation. Swiss-Prot annotation depends on the correct association of sequences to gene families and proteins with known function, using the available literature as a reference. The annotation is facilitated since each of the genes is associated with PubMed Identifiers (PMIDs) stored in the PESCADOR tool, which are important references for the related orthologs.

The search for functional information for the *D. melanogaster* orthologues revealed the involvement of the genes in processes related to the embryonic development and was also a good validation for the clustering by SeedServer, since all sequences from *D. melanogaster* that clustered to the initial human and mouse genes present an embryo development related function.

Generation of correct clusters is essential for the correct determination of gene ancestry, but it is not the sole limiting factor. Sequencing of key organisms from taxonomic outgroups relative to the ones with complete genome sequences available will be a crucial source of sequences that will allow a revaluation of gene ancestry. Meanwhile, additional sequences clustered by software (SeedLinkage) and database enrichment (UEKO) improve the inspection of ancestry.

Determination of the ancestry for the genes in the preimplantation pathway was nonetheless a central analysis, given the expectancy that this pathway would be mainly formed by more contemporary components. Our data suggest that an ancient fraction of the pathway including Nanog and Sox2 originated before Chordata, whereas a modern fraction including Oct4 and LIF has appeared near the origin of Eutheria, the placentary organisms. Thus, an important transcriptional pathway comprising ancient and modern members has been characterized with text mining, and homologues search with SeedServer promptly allowed LCA determination.

## Conclusions

Generation of regulatory pathways through text-mining tools allows integration of data generated by previous studies for a more complete view of a biological process. If the genes present in this pathway are associated with clusters of orthologues this information is added to the pathway making the visualization of the same process available for different organisms. The analysis of orthology also permits determination of the ancestry of the genes involved in the process leading to a better understanding of the evolution of such process.

## Methods

### Text-mining and pathway construction

NCBI’s PubMed database was used as a source of available literature (http://www.ncbi.nlm.nih.gov/pubmed) for the text-mining approach. The search query used was “preimplantation development” and the PubMed identification numbers of the selected papers (PMIDs) were saved as a text file. Ten papers were selected manually by us to be used in the Medline Ranker software ([19]; http://cbdm.mdc-berlin.de/tools/medlineranker/). These papers, (references [23,26,28,29,31,50,59,81,82]), were considered by us as highly informative because they described numerous gene regulations concerning preimplantation development. We used the PMIDs retrieved by the PubMed search as the background set and the 10 manually selected PMIDs as the training set. After classification by order of relevance we selected the 1000 better-classified abstracts for further analysis presenting a p-value < 0.01. These abstracts were then submitted through PESCADOR (manuscript under preparation, Barbosa-Silva *et al*.), an online platform for the software LAITOR [20]. After PESCADOR, results were manually curated and the gene biointeractions recovered were used to build a regulatory pathway in Keynote MacOS according to the markup language used by KEGG for pathway construction (KGML can be found at http://www.genome.jp/kegg/xml/docs/). This process consisted mainly of finding the highlighted interaction in the abstract tagged by PESCADOR, confirming its involvement in the preimplantation development by checking the corresponding paper and drawing this interaction in the pathway picture.

### SeedServer search for homologues

UniProt IDs for human and mouse gene products corresponding to each of the genes represented in the preimplantation pathway were used as seed in the SeedServer software (not published, Guedes et al.). SeedServer is a web application (http://biodados.icb.ufmg.br/seedserver/) which searches for homologous sequences through two components: the program SeedLinkage [78] and the databases KEGG Orthology (KO) [5] and its enriched version UEKO (unpublished, developed by Fernandes *et al.* by application of the procedure described to enrich COG [10] to the KEGG Orthology database). Clustering was verified by PSI-BLAST searches using seed sequences as query and the recruited proteins as database, and eventual false positives were discarded (1.5% of the recruited sequences).

### LCA determination

Clusters generated for each of the pathway genes were used to determine the Last Common Ancestor (LCA) of each gene. Each cluster provided a list of Taxonomy IDs corresponding to the organisms in which orthologs of the pathway genes were found. The clade in the human lineage that comprised these Taxonomy IDs as leaves in the Taxonomy Tree was considered to bear the LCA.

## Note added in proof

PESCADOR, referred in the text as in preparation, is now published: PESCADOR, a web-based tool to assist text-mining of biointeractions extracted from PubMed queries. Barbosa-Silva A, Fontaine JF, Donnard ER, Stussi F, Ortega JM, Andrade-Navarro MA. BMC Bioinformatics. 2011 Nov 9;12(1):435. [Epub ahead of print] PMID: 22070195[83].

## Competing interests

The authors declare that they have no competing interests.

## Authors' contributions

ERD and JMO conceived the project and wrote the paper. ERD performed the research and pathway construction. MJK curated the pathway biointeractions. ABS (author of SeedLinkage and LAITOR) and MAAN designed the PESCADOR platform. RLMG designed the SeedServer software and conducted the ortholog search. HV was responsible for the LCA determination. GRF constructed the UEKO database. All authors read and approved the final manuscript.

## Supplementary Material

Additional file 1 **Homolog clusters.** Clusters of homologous sequences found by SeedServer for each of the genes in the preimplantation pathway. For each gene, the left column shows the clustered sequence Uniprot ID and the right column shows the Taxonomy ID for this sequence. Click here for file

Additional file 2 **Ortholog functions in *Drosophila melanogaster*.** This figure represents the corresponding *D. melanogaster* orthologs found by SeedServer and their respective interactions and functions in fruit fly development. Note that these orthologs are involved in processes related to *D. melanogaster* embryo development. See Additional file 3 for a table with gene name correspondence between the genes in this figure and the ones on Figure 3. Click here for file

Additional file 3 **Gene correspondence table.** Human and *Drosophila melanogaster* gene name correspondence for the orthologs grouped by SeedServer. Column 3 lists the PubMed identifiers (PMIDs) from the papers where functions described in Additional file 2 were found. Click here for file
