# Supplementary material for: Preimplantation development regulatory pathway construction through a text-mining approach
Source: BMC Genomics. 2011 Dec 22;12(Suppl 4):S3. doi: 10.1186/1471-2164-12-S4-S3 (PMC3287586; doi:10.1186/1471-2164-12-S4-S3)
Supplement: Additional file 1 — Homolog clusters. Clusters of homologous sequences found by SeedServer for each of the genes in the preimplantation pathway. For each gene, the left column shows the clustered sequence Uniprot ID and the right column shows the Taxonomy ID for this sequence. [file 1471-2164-12-S4-S3-S1.pdf]

CLUSTERS

| symbol CTCFL (BORIS) | |
|---|---|
| A2APF3 | 10090 |
| A5GFN3 | 9823 |
| B0WNM1 | 7176 |
| B0WNM2 | 7176 |
| B1H2R4 | 8364 |
| B2CMK7 | 9315 |
| B2CMK8 | 9258 |
| B2CMK9 | 103695 |
| B2CML1 | 9315 |
| B4J365 | 7222 |
| B4LFC4 | 7244 |
| B4MLE6 | 7260 |
| B4PK64 | 7245 |
| B4QKD1 | 7240 |
| B6PZY1 | 7739 |
| B9EKP6 | 10090 |
| P49711 | 9606 |
| Q08705 | 9031 |
| Q08DH9 | 9913 |
| Q16EW4 | 7159 |
| Q3Y6S0 | 10090 |
| Q4H2H9 | 7719 |
| Q4H2I0 | 7719 |
| Q4KR73 | 7159 |
| Q4SIG6 | 99883 |
| Q53XI7 | 9606 |
| Q61164 | 10090 |
| Q6JAA4 | 7955 |
| Q6PF79 | 8355 |
| Q8NI51 | 9606 |
| Q8TA44 | 7227 |
| Q8WPX1 | 7656 |
| Q9DDQ9 | 8355 |
| Q9R1D1 | 10116 |
| Q9VS55 | 7227 |

| symbol Zfp42 (REX1) | |
|---|---|
| P22227 | 10090 |
| Q96MM3 | 9606 |

| symbol LAMB3 | |
|---|---|
| Q13751 | 9606 |
| A2VDM1 | 9913 |
| Q91V90 | 10090 |
| Q61087 | 10090 |

| symbol DPPA2 (ECSA) | |
|---|---|
| Q7Z7J5 | 9606 |
| Q9CWH0 | 10090 |
| B2RQ54 | 10090 |

| symbol SNAI1 | |
|---|---|
| O95863 | 9606 |
| Q6AY35 | 10116 |
| Q02085 | 10090 |
| Q4FK48 | 10090 |
| Q6JBR3 | 45264 |
| B2R842 | 9606 |
| B5X301 | 8030 |
| P19382 | 8355 |
| Q4SLL9 | 99883 |

| symbol HAND1 | |
|---|---|
| O96004 | 9606 |
| Q0VCE2 | 9913 |
| P97832 | 10116 |
| P57100 | 9986 |
| Q64279 | 10090 |
| Q5SQG1 | 10090 |
| Q80UL7 | 10090 |
| Q58L58 | 9823 |
| Q28555 | 9940 |
| Q28JA1 | 8364 |
| Q90691 | 9031 |
| O73615 | 8355 |
| B7ZRJ2 | 8355 |
| Q4SPY5 | 99883 |
| Q4H3E6 | 7719 |
| Q75UU2 | 51511 |
| A7S670 | 45351 |
| P61295 | 10116 |
| P61296 | 9606 |
| Q29CX1 | 46245 |
| Q5XJD8 | 7955 |
| Q61039 | 10090 |
| Q90690 | 9031 |
| Q9VL05 | 7227 |
| A4QNK1 | 8364 |
| B0BLY7 | 8355 |
| B6ECG9 | 9606 |
| B6KXY3 | 7739 |
| P57101 | 8355 |
| Q9EPN2 | 10090 |
| B3N962 | 7220 |
| B4G7J0 | 7234 |
| B4HWJ7 | 7238 |
| B4KJF8 | 7230 |
| B4NZ86 | 7245 |
| B4Q8S6 | 7240 |
| A2BDM6 | 7955 |
| P57102 | 7955 |

| symbol Sap18 | |
|---|---|
| O55128 | 10090 |
| Q3MHS8 | 10116 |
| Q3T022 | 9913 |
| Q5RDT5 | 9601 |
| O00422 | 9606 |
| B2R494 | 9606 |
| Q90ZH5 | 9031 |
| B5G450 | 59729 |
| B5G461 | 59729 |
| Q28FL5 | 8364 |
| Q6PC41 | 7955 |
| Q5EAY9 | 8355 |
| Q4SEE6 | 99883 |
| B5XE12 | 8030 |
| B9EQ61 | 8030 |
| B9EQ87 | 8030 |
| Q09JJ5 | 34602 |
| B6P369 | 7739 |
| B4QX67 | 7240 |
| B4PR29 | 7245 |
| B3P0Z2 | 7220 |
| Q9VEX9 | 7227 |
| B4HLC7 | 7238 |
| B4GMJ9 | 7234 |
| B3LZX9 | 7217 |
| B4KA04 | 7230 |
| B4NL01 | 7260 |
| B4LY00 | 7244 |
| B4JG20 | 7222 |
| Q294V7 | 46245 |
| Q16GI2 | 7159 |
| A7RY60 | 45351 |
| Q7PR28 | 7165 |
| B0XBD8 | 7176 |
| B0XKQ7 | 7176 |
| A8Q2P3 | 6279 |
| B3RYA5 | 10228 |
| Q09250 | 6239 |
| Q5XUB8 | 223852 |
| Q5UEN2 | 223852 |
| Q5DEW0 | 6182 |
| Q1E366 | 5501 |
| Q4FZH3 | 10090 |

| symbol DPPA5 (ESG1) | |
|---|---|
| A6NC42 | 9606 |
| B7NZC1 | 9986 |
| Q9CQS7 | 10090 |
| P85965 | 10090 |

CLUSTERS

| symbol DPPA4 | |
|---|---|
| Q7L190 | 9606 |
| Q8CCG4 | 10090 |
| Q9CWG7 | 10090 |
| B7Z5Q7 | 9606 |

| ymbol 1700011E24Rik (GSE | |
|---|---|
| Q9DAG5 | 10090 |
| Q8N801 | 9606 |
| B6LAL7 | 7739 |
| B6PLG8 | 7739 |
| B3RJY3 | 10228 |

| symbol GATA2 | |
|---|---|
| A7RFY2 | 45351 |
| A8Q0N5 | 6279 |
| A9ZND4 | 138676 |
| B0X8S3 | 7176 |
| B2DBE1 | 10116 |
| B3DGG5 | 7955 |
| B3LZW4 | 7217 |
| B3NZW9 | 7220 |
| B3S690 | 10228 |
| B4G4Y3 | 7234 |
| B4HLP8 | 7238 |
| B4JF53 | 7222 |
| B4M159 | 7244 |
| B4NJ90 | 7260 |
| B4PSL4 | 7245 |
| B6LGL9 | 7739 |
| B7WNQ9 | 9606 |
| B7Z0V4 | 7227 |
| B8PJZ6 | 561896 |
| O09100 | 10090 |
| O77156 | 7668 |
| P15976 | 9606 |
| P17678 | 9031 |
| P17679 | 10090 |
| P23769 | 9606 |
| P23770 | 8355 |
| P23771 | 9606 |
| P23772 | 10090 |
| P23773 | 8355 |
| P23824 | 9031 |
| P23825 | 9031 |
| P43429 | 10116 |
| P91623 | 7227 |
| Q08DV0 | 9913 |
| Q0ZHH4 | 9823 |
| Q172Z8 | 7159 |
| Q299A5 | 46245 |
| Q2TAR8 | 8355 |

| symbol GATA2 | |
|---|---|
| Q32NN9 | 8355 |
| Q3B845 | 10090 |
| Q3TZD6 | 10090 |
| Q3TZS0 | 10090 |
| Q3U0R5 | 10090 |
| Q3U223 | 10090 |
| Q3U320 | 10090 |
| Q3UIH9 | 10090 |
| Q53YE0 | 9606 |
| Q560R5 | 5207 |
| Q59IU1 | 8090 |
| Q5KA62 | 5207 |
| Q5KPR4 | 5207 |
| Q5KST7 | 8090 |
| Q5VWG7 | 9606 |
| Q5VWG8 | 9606 |
| Q60I22 | 7764 |
| Q6DG89 | 7955 |
| Q6DIQ6 | 8364 |
| Q6S5H3 | 45351 |
| Q7T3G1 | 7955 |
| Q7TMX8 | 10090 |
| Q865U9 | 9823 |
| Q8QFW5 | 33514 |
| Q91428 | 7955 |
| Q924Y4 | 10116 |
| Q96BH0 | 9606 |
| Q96BH8 | 9606 |
| Q99NH5 | 10116 |
| Q9DBY9 | 10090 |
| Q9DC59 | 10090 |

| symbol WNT9A | |
|---|---|
| O14904 | 9606 |
| Q8R5M2 | 10090 |
| Q3L251 | 9031 |
| O42280 | 9031 |
| B6V3E1 | 7955 |
| Q0P406 | 7955 |
| B6L7F0 | 7739 |
| B6NS64 | 7739 |
| B7PV27 | 6945 |
| O14905 | 9606 |
| Q8C718 | 10090 |
| A4ZGP2 | 8364 |
| B6V3E2 | 7955 |
| O35468 | 10090 |
| Q2TBA6 | 10090 |
| Q6NZE9 | 10090 |
| Q6ZS96 | 9606 |
| Q6ZSP0 | 9606 |

| symbol Eomes | |
|---|---|
| O54839 | 10090 |
| Q9JJL1 | 10090 |
| Q52KJ1 | 10090 |
| Q8BN22 | 10090 |
| O95936 | 9606 |
| B3DL32 | 8364 |
| Q5XH32 | 8355 |
| P79944 | 8355 |
| A0JMS3 | 8355 |
| Q9PVX6 | 8330 |
| Q98TU2 | 7955 |
| Q9DDU3 | 7955 |
| B2GTZ2 | 8364 |
| B7Z5W6 | 9606 |
| B7Z619 | 9606 |

| symbol LEFTY1/LEFTY2 | |
|---|---|
| O75610 | 9606 |
| B2R7U0 | 9606 |
| O00292 | 9606 |
| B4E332 | 9606 |
| P57785 | 10090 |
| Q64280 | 10090 |
| Q52M97 | 8355 |
| Q5UCE3 | 10116 |
| Q9PW55 | 7955 |
| Q9W6I6 | 7955 |
| Q9W6I7 | 7955 |
| Q95YK6 | 51511 |
| B6LUA8 | 7739 |
| B3FNR6 | 7668 |
| Q6T265 | 7656 |
| A0A7U9 | 8255 |
| B3DLA0 | 8364 |
| A2TJL5 | 8090 |
| Q9PVN4 | 9031 |
| Q4SGU3 | 99883 |
| Q4H393 | 7719 |
| Q9DD36 | 8355 |
| Q9DFC5 | 8355 |
| Q9DFC6 | 8355 |

| symbol Gcm1 | |
|---|---|
| P70348 | 10090 |
| Q9Z288 | 10116 |
| Q9NP62 | 9606 |
| Q6UEJ0 | 9031 |
| Q7ZY38 | 8355 |
| B7QBR5 | 6945 |
| A9YWQ5 | 7654 |
| Q3UQD1 | 10090 |

CLUSTERS

| symbol Krt18 | |
|---|---|
| P05784 | 10090 |
| A2BDA6 | 8355 |
| Q5BJY9 | 10116 |
| P05783 | 9606 |
| B2RA03 | 9606 |
| Q6P864 | 8364 |
| Q7ZTS4 | 7955 |
| Q5K2N9 | 7886 |
| Q7SYF8 | 27689 |
| O57607 | 8022 |
| Q07427 | 7957 |
| O57611 | 68454 |
| A1KQY9 | 55291 |
| Q4SRP1 | 99883 |
| Q8AWA6 | 7748 |
| Q8AWA8 | 7748 |
| Q8AWA7 | 7748 |
| A1L317 | 10090 |
| A1L595 | 9913 |
| A3KN26 | 9913 |
| A3RF35 | 9615 |
| A5A6M0 | 9598 |
| A5A6M5 | 9598 |
| A5A6M9 | 37012 |
| A5A6N2 | 9598 |
| A5A6P3 | 9598 |
| A5PJJ1 | 9913 |
| A6QNZ7 | 9913 |
| A6QP62 | 9913 |
| A6QP90 | 9913 |
| A6QQQ9 | 9913 |
| A7YWM2 | 9913 |
| B0BMG5 | 8364 |
| B6PNK6 | 7739 |
| O18740 | 9615 |
| O76009 | 9606 |
| O76011 | 9606 |
| O76013 | 9606 |
| O76014 | 9606 |
| O93256 | 9031 |
| P02533 | 9606 |
| P02535 | 10090 |
| P06394 | 9913 |
| P08727 | 9606 |
| P08728 | 9913 |
| P08730 | 10090 |
| P08778 | 8355 |
| P08779 | 9606 |
| P13645 | 9606 |
| P13646 | 9606 |
| P19001 | 10090 |

| symbol Krt18 | |
|---|---|
| P19012 | 9606 |
| P25030 | 10116 |
| P35527 | 9606 |
| P35900 | 9606 |
| Q04695 | 9606 |
| Q05AL9 | 7955 |
| Q0P5J4 | 9913 |
| Q0P5J6 | 9913 |
| Q0P5J7 | 9913 |
| Q14525 | 9606 |
| Q14532 | 9606 |
| Q148H6 | 9913 |
| Q148I8 | 9913 |
| Q148J0 | 9913 |
| Q15323 | 9606 |
| Q17QL7 | 9913 |
| Q1LXJ1 | 7955 |
| Q1LXJ7 | 7955 |
| Q1RLR3 | 7955 |
| Q2M2I5 | 9606 |
| Q2VPN9 | 8355 |
| Q497I4 | 10090 |
| Q4QR57 | 8355 |
| Q568U3 | 7955 |
| Q5XGU0 | 8355 |
| Q61414 | 10090 |
| Q61765 | 10090 |
| Q61781 | 10090 |
| Q62168 | 10090 |
| Q63279 | 10116 |
| Q64291 | 10090 |
| Q6A162 | 9606 |
| Q6A163 | 9606 |
| Q6DDD5 | 8355 |
| Q6DG66 | 7955 |
| Q6DHB6 | 7955 |
| Q6DHU3 | 7955 |
| Q6DK80 | 8364 |
| Q6DKC7 | 8355 |
| Q6EIZ0 | 9615 |
| Q6IFU7 | 10116 |
| Q6IFU8 | 10116 |
| Q6IFU9 | 10116 |
| Q6IFV0 | 10116 |
| Q6IFV1 | 10116 |
| Q6IFV3 | 10116 |
| Q6IFV4 | 10116 |
| Q6IFV5 | 10116 |
| Q6IFV6 | 10116 |
| Q6IFV7 | 10116 |
| Q6IFV8 | 10116 |

| symbol Krt18 | |
|---|---|
| Q6IFV9 | 10116 |
| Q6IFW0 | 10116 |
| Q6IFW1 | 10116 |
| Q6IFW2 | 10116 |
| Q6IFW3 | 10116 |
| Q6IFW4 | 10116 |
| Q6IFW5 | 10116 |
| Q6IFW6 | 10116 |
| Q6IFW8 | 10116 |
| Q6IFX0 | 10116 |
| Q6IFX2 | 10090 |
| Q6IFX3 | 10090 |
| Q6IFX4 | 10090 |
| Q6IRP5 | 8355 |
| Q6P0E4 | 7955 |
| Q6P7K6 | 8364 |
| Q6PVZ1 | 9031 |
| Q6PVZ2 | 9031 |
| Q6RHW0 | 10090 |
| Q7SY65 | 8355 |
| Q7Z3Y7 | 9606 |
| Q7Z3Z0 | 9606 |
| Q7ZXE6 | 8355 |
| Q7ZYN9 | 8355 |
| Q803Y5 | 7955 |
| Q8CIS9 | 10116 |
| Q8K0Y2 | 10090 |
| Q8VCW2 | 10090 |
| Q92764 | 9606 |
| Q99456 | 9606 |
| Q99PS0 | 10090 |
| Q9C075 | 9606 |
| Q9D312 | 10090 |
| Q9D646 | 10090 |
| Q9GJW4 | 9598 |
| Q9PUB6 | 7955 |
| Q9QWL7 | 10090 |
| Q9Z2K1 | 10090 |
| Q9Z320 | 10090 |
| A1A4E9 | 9606 |
| A4UYK3 | 28829 |
| A5A6P4 | 37012 |
| A6BLY7 | 10090 |
| A6H712 | 9913 |
| A7TUG6 | 8400 |
| A8K2H9 | 9606 |
| A8K488 | 9606 |
| B0BMH1 | 8364 |
| B0BMI0 | 8364 |
| B0BMI8 | 8364 |
| B0LKN9 | 9940 |

CLUSTERS

| symbol Krt18 | |
|---|---|
| B1AQ77 | 10090 |
| B1AQ78 | 10090 |
| B2GUG8 | 8364 |
| B2R9E0 | 9606 |
| B2RU72 | 10090 |
| B2ZHC2 | 10141 |
| B3DM07 | 8364 |
| B3KVF5 | 9606 |
| B7ZRD4 | 8355 |
| B7ZSA0 | 8355 |
| O77727 | 9940 |
| P02537 | 8355 |
| P05781 | 8355 |
| P51856 | 9310 |
| Q05AX6 | 8355 |
| Q1LXJ0 | 7955 |
| Q3SYP5 | 10090 |
| Q3TRJ4 | 10090 |
| Q3ZAW8 | 10090 |
| Q505L4 | 8355 |
| Q6IFW9 | 10116 |
| Q6IFX1 | 10116 |
| Q6R649 | 9925 |
| Q6R650 | 9925 |
| Q7Z3Y8 | 9606 |
| Q7Z3Y9 | 9606 |
| Q8AV12 | 8355 |
| Q8JFQ6 | 8022 |
| Q98UJ1 | 8355 |
| Q98UJ2 | 8355 |
| Q9BGM5 | 9940 |
| Q9EQD6 | 10090 |
| Q9EQD7 | 10090 |
| B2GU21 | 8364 |
| Q1LXJ8 | 7955 |
| Q28IM9 | 8364 |
| Q6IFW7 | 10116 |
| Q6ZPD6 | 9606 |
| B0LKP0 | 9940 |
| B0LKP2 | 9940 |
| B1AQ75 | 10090 |
| B1ATJ5 | 10090 |
| B6Z1W3 | 28377 |
| B7ZM39 | 9606 |
| P02534 | 9940 |
| P25690 | 9940 |
| Q148N4 | 10090 |
| Q61897 | 10090 |
| Q6R651 | 9925 |
| Q9BGM6 | 9940 |
| Q9GJW5 | 9593 |

| symbol Krt18 | |
|---|---|
| A2A513 | 10090 |
| A2VDC4 | 8355 |
| B0UYS0 | 7955 |
| B6Z1W4 | 28377 |
| P08777 | 8355 |
| Q07426 | 7957 |
| Q0X0A4 | 8355 |
| Q7SYG4 | 27689 |
| Q7T0X6 | 8355 |
| Q90303 | 7957 |
| Q9NG14 | 7740 |
| Q9NG16 | 7740 |
| Q9XYG9 | 7739 |
| A2RRM5 | 9606 |
| B0LKP1 | 9940 |
| O76015 | 9606 |
| Q5K2N2 | 7886 |
| Q5K2P1 | 7886 |
| Q5K2P4 | 7886 |
| Q5K2P5 | 7886 |
| Q5K2P6 | 7886 |
| P08802 | 8355 |
| A4UYK4 | 28829 |
| B1AQ76 | 10090 |
| Q4RZM7 | 99883 |
| Q5R8S9 | 9601 |
| Q9PWD8 | 7955 |
| A1L222 | 7955 |
| A2SXI5 | 8267 |
| Q1LXJ2 | 7955 |
| Q1LXJ9 | 7955 |
| Q1LXK0 | 7955 |
| Q1RM74 | 7955 |
| Q3ULX3 | 10090 |
| Q544I8 | 10090 |
| Q8CB30 | 10090 |
| Q8TC04 | 9606 |
| B4DL17 | 9606 |
| Q28FN7 | 8364 |
| Q2KI75 | 9913 |
| Q8CCX5 | 10090 |
| Q8N1A0 | 9606 |

| symbol Mybl2 | |
|---|---|
| P48972 | 10090 |
| P10244 | 9606 |
| B2RBS5 | 9606 |
| B7Z8D9 | 9606 |
| Q17QY0 | 9913 |
| Q03237 | 9031 |
| P52551 | 8355 |
| Q6NRE6 | 8355 |
| Q4KMF7 | 7955 |
| Q6DRH7 | 7955 |
| A2A5P2 | 10090 |
| P10243 | 9606 |
| P51960 | 10090 |
| P52550 | 9031 |
| Q3ZB50 | 10090 |
| Q495F9 | 9606 |
| Q495G0 | 9606 |

| symbol Jak1 | |
|---|---|
| P52332 | 10090 |
| B1ASP2 | 10090 |
| Q3URU8 | 10090 |
| Q3U8P8 | 10090 |
| P23458 | 9606 |
| Q4LDX3 | 9606 |
| Q9TTJ1 | 9823 |
| Q9PWM9 | 9031 |
| Q6DDJ0 | 8355 |
| Q7ZU16 | 7955 |
| O12990 | 7955 |
| Q09178 | 7962 |
| O57612 | 47145 |
| B4H0H6 | 7234 |
| B9EQC7 | 8030 |

| symbol DAB2 | |
|---|---|
| P98082 | 9606 |
| P98078 | 10090 |
| O88797 | 10116 |
| Q5RF13 | 9601 |
| B5X191 | 8030 |
| Q4S2B9 | 99883 |
| Q6PAY5 | 8355 |
| A1XI03 | 8355 |
| Q6PAV7 | 8355 |
| B8A566 | 7955 |
| Q7SYH8 | 7955 |
| Q6P0K3 | 7955 |
| A8Q553 | 6279 |
| B2RAW0 | 9606 |
| B3S3X8 | 10228 |

CLUSTERS

| symbol DNMT1 | |
|---|---|
| P26358 | 9606 |
| Q24K09 | 9913 |
| Q4TTV6 | 9823 |
| Q865V5 | 9940 |
| P13864 | 10090 |
| Q7TSJ0 | 10090 |
| Q3UHZ3 | 10090 |
| Q9Z330 | 10116 |
| Q8MJ28 | 13616 |
| Q27746 | 7656 |
| Q8IAJ7 | 7656 |
| O42803 | 5191 |
| O42731 | 5191 |
| A8NEZ8 | 240176 |
| A8N3W7 | 240176 |
| B2AKW2 | 5145 |
| A7ERM2 | 325569 |
| B0B066 | 5147 |
| Q96W73 | 5141 |
| Q1E6Q1 | 5501 |
| Q0UYX5 | 13684 |
| B6Q1V3 | 441960 |
| B7ZLW6 | 9606 |
| Q92072 | 9031 |
| Q6GQH0 | 8355 |
| P79922 | 8355 |
| B1WBA5 | 8355 |
| Q9I8X6 | 96538 |
| B6NJV2 | 7739 |
| Q2GQX6 | 38033 |
| A1DLL2 | 331117 |
| A2R4V8 | 425011 |
| A4RBT3 | 148305 |
| A4RDS0 | 148305 |
| A6RJV4 | 332648 |
| A6S0I6 | 332648 |
| A7E505 | 325569 |
| A7UVZ2 | 5141 |
| B0CNH7 | 486041 |
| B0CPJ9 | 486041 |
| B0CSP1 | 486041 |
| B2AUK7 | 5145 |
| B3MVH6 | 7217 |
| B3RL43 | 10228 |
| B6L3A0 | 7739 |
| B6PIX4 | 7739 |
| O14717 | 9606 |
| O55055 | 10090 |
| P40999 | 4896 |
| Q0E8R4 | 7227 |
| Q177E1 | 7159 |

| symbol DNMT1 | |
|---|---|
| Q29NK7 | 46245 |
| Q2KFY7 | 242507 |
| Q2U949 | 5062 |
| Q4G073 | 10116 |
| Q4W5Z2 | 9031 |
| Q4WD73 | 5085 |
| Q588C1 | 7955 |
| Q6DIS0 | 8364 |
| Q7YS61 | 9913 |
| Q7ZTJ0 | 8355 |
| Q876R1 | 162425 |
| Q8QGB8 | 7955 |
| A1CMG6 | 5057 |
| B0YD76 | 451804 |
| Q0CGV2 | 341663 |
| Q8NJV9 | 5142 |
| Q8NJW0 | 5141 |
| Q9HFI8 | 5141 |
| B3DKL6 | 7955 |
| B3DKL7 | 7955 |
| B6VB52 | 7957 |
| Q8IAJ2 | 7656 |
| Q8IAJ3 | 7656 |
| Q8IAJ4 | 7656 |
| B0XCF5 | 7176 |
| B0YJ02 | 9606 |
| B0YJ03 | 9606 |
| B4DQZ2 | 9606 |
| B7Z1Y7 | 9606 |
| Q29R90 | 7955 |
| Q5I0V6 | 10090 |
| Q8C7F0 | 10090 |
| Q8CE27 | 10090 |
| B6K0C0 | 402676 |
| B3N3X2 | 7220 |
| B4GQK4 | 7234 |
| B4IE64 | 7238 |
| B4JBL7 | 7222 |
| B4KI02 | 7230 |
| B4LSF6 | 7244 |
| B4P1Y6 | 7245 |
| B4Q3Q2 | 7240 |
| Q8MSZ1 | 7227 |
| Q9U6H7 | 7227 |
| Q9VKB3 | 7227 |

| symbol Dsc2 | |
|---|---|
| P55292 | 10090 |
| Q3T1K6 | 10116 |
| Q02487 | 9606 |
| A9X9K9 | 9606 |
| A8K2P8 | 9606 |
| A9X9L0 | 9606 |
| B4DLJ5 | 9606 |
| Q63HM4 | 9606 |
| Q544V1 | 10090 |
| Q6PEU5 | 10090 |
| B3DLX7 | 8364 |
| A6QR67 | 9913 |
| A8K6T3 | 9606 |
| P55849 | 10090 |
| P55850 | 10090 |
| Q01107 | 9913 |
| Q14574 | 9606 |
| Q32ME9 | 10090 |
| Q8C4K6 | 10090 |
| Q9HB00 | 9606 |

| symbol Nr0b1 (DAX1) | |
|---|---|
| Q61066 | 10090 |
| P70503 | 10116 |
| Q9BG94 | 9483 |
| P79386 | 9823 |
| Q9BG93 | 9544 |
| Q9BG97 | 9598 |
| Q9BG96 | 9600 |
| P51843 | 9606 |
| Q9PTE9 | 9031 |
| Q8QGS4 | 8410 |
| A3KNP5 | 7955 |
| Q59AE8 | 13489 |
| A7BFV4 | 8090 |
| Q8AY13 | 8128 |
| Q1A7P2 | 8128 |
| Q8AUM4 | 8128 |
| B6MX29 | 7739 |
| B6PIM4 | 7739 |
| Q53ZY9 | 10090 |

CLUSTERS

| symbol RPS14 | |
|---|---|
| P62263 | 9606 |
| P62264 | 10090 |
| P62265 | 10029 |
| Q6PI76 | 8355 |
| Q5ZHW8 | 9031 |
| Q5DVH5 | 8260 |
| Q4SQ18 | 99883 |
| B5G2V9 | 59729 |
| A4IGS7 | 8364 |
| A2IBV8 | 13489 |
| Q6PDV6 | 10116 |
| Q6PBW3 | 7955 |
| B5DGX1 | 8030 |
| A9Z0N4 | 173242 |
| Q90YR0 | 7998 |
| A2Q0S9 | 28829 |
| Q08699 | 6096 |
| B6L4I6 | 7739 |
| Q3T076 | 9913 |
| Q6IV88 | 155462 |
| B0Z9Q5 | 56195 |
| Q0PXX5 | 121845 |
| B4LK78 | 7244 |
| B4M2G0 | 7244 |
| B4M715 | 7244 |
| B4KU90 | 7230 |
| B4L4Q3 | 7230 |
| B4L542 | 7230 |
| B6RB78 | 91233 |
| B4JN94 | 7222 |
| B4JLX6 | 7222 |
| B4JUT2 | 7222 |
| Q6EUY9 | 285223 |
| A7S2J5 | 45351 |
| Q1HR24 | 7159 |
| Q4GXR8 | 292460 |
| A6N9P0 | 140564 |
| Q6B8A8 | 29930 |
| Q4PM10 | 6945 |
| Q9XYQ4 | 35570 |
| B5DKT4 | 46245 |
| B5DKT3 | 46245 |
| B5DRY2 | 46245 |
| B4H3V3 | 7234 |
| B4G5E9 | 7234 |
| B4H871 | 7234 |
| Q56FC4 | 77504 |
| Q8WSQ7 | 27405 |
| A9UDP3 | 478208 |
| A2I3Z5 | 177089 |
| B3NXZ6 | 7220 |

| symbol RPS14 | |
|---|---|
| B3NXZ7 | 7220 |
| P14130 | 7227 |
| B3MRI2 | 7217 |
| B3MRI3 | 7217 |
| B4NU46 | 7240 |
| B4N1W4 | 7260 |
| B4NMG4 | 7260 |
| Q7QBX2 | 7165 |
| Q7QEH1 | 7165 |
| B3S4M7 | 10228 |
| B6DDR5 | 43151 |
| B4IL57 | 7238 |
| B4IL58 | 7238 |
| Q962R5 | 7108 |
| Q4GXR9 | 197013 |
| Q6F434 | 51655 |
| Q5UAM9 | 7091 |
| Q66SW2 | 7091 |
| P48150 | 6239 |
| B4Q0P9 | 7245 |
| A9UTG2 | 81824 |
| A1BQ64 | 6279 |
| P48855 | 6728 |
| A1BQ87 | 34506 |
| Q4KTC8 | 55567 |
| Q2H733 | 38033 |
| Q56J82 | 317012 |
| Q5EN09 | 148305 |
| B2AXS3 | 5145 |
| A7E468 | 325569 |
| A6S046 | 332648 |
| Q0V1M6 | 13684 |
| Q0CXQ6 | 341663 |
| A6REL0 | 339724 |
| B6QPA0 | 441960 |
| B8M8L7 | 441959 |
| Q1DZB5 | 5501 |
| A2QD79 | 425011 |
| Q4X1C6 | 5085 |
| B0XS79 | 451804 |
| A1DGX0 | 331117 |
| A1C695 | 5057 |
| Q2U044 | 5062 |
| B8NB42 | 332952 |
| P19115 | 5141 |
| Q5B0H0 | 162425 |
| B6HR74 | 500485 |
| B2WF70 | 426418 |
| B8P8W4 | 561896 |
| Q5KFT0 | 5207 |
| B0D6C8 | 486041 |

| symbol RPS14 | |
|---|---|
| A8NNH7 | 240176 |
| A8QA30 | 425265 |
| Q6BHV6 | 4959 |
| A3LQJ1 | 4924 |
| Q96W53 | 5476 |
| B2G3U1 | 4956 |
| B2G3T1 | 4956 |
| P06367 | 4932 |
| P39516 | 4932 |
| B3LUA2 | 285006 |
| B3LPT6 | 285006 |
| A6ZTL2 | 307796 |
| A6ZQE5 | 307796 |
| P27069 | 28985 |
| A7TFH1 | 436907 |
| Q758E0 | 33169 |
| Q6CA55 | 4952 |
| Q6FKW5 | 5478 |
| Q6FNE1 | 5478 |
| O14150 | 4896 |
| B6K471 | 402676 |
| B6JY89 | 402676 |
| B2R5G5 | 9606 |
| P13471 | 10116 |
| Q4PA42 | 5270 |
| Q8SSA6 | 6035 |
| B4GQF6 | 7234 |
| B5DJ85 | 46245 |
| B5G2W0 | 59729 |
| O70569 | 10090 |
| Q3UJS5 | 10090 |
| Q6GQQ8 | 9606 |
| B3MH77 | 7217 |
| A5DSS0 | 379508 |
| A7TGT1 | 436907 |
| B4IS84 | 7234 |
| B8XVP3 | 329105 |

| symbol SALL4 | |
|---|---|
| Q9UJQ4 | 9606 |
| A5GFR9 | 9823 |
| A2AV00 | 10090 |
| Q8BX22 | 10090 |
| Q5F3B8 | 9031 |
| A4D0F7 | 9031 |
| Q6NX24 | 8364 |
| Q7T184 | 8355 |
| Q640I8 | 8355 |
| Q9PVN7 | 8355 |
| A0MWC1 | 7955 |
| Q27ZK0 | 54126 |

CLUSTERS

| symbol EIF2B1 | |
|---|---|
| Q14232 | 9606 |
| Q5RAR0 | 9601 |
| Q4R4V8 | 9541 |
| Q0IIF2 | 9913 |
| Q99LC8 | 10090 |
| Q3UZR8 | 10090 |
| Q64270 | 10116 |
| B5XAS9 | 8030 |
| B5DG92 | 8030 |
| B5XGV9 | 8030 |
| Q4S231 | 99883 |
| A5WWE5 | 7955 |
| A5WWE4 | 7955 |
| Q6IQQ8 | 7955 |
| Q28D56 | 8364 |
| Q6NUG3 | 8355 |
| B6M402 | 7739 |
| A7SMK4 | 45351 |
| Q0ZB82 | 7091 |
| A9UQZ5 | 81824 |
| B0W6F7 | 7176 |
| B3LYV6 | 7217 |
| Q9VAD4 | 7227 |
| Q9GQ89 | 7227 |
| B3P819 | 7220 |
| B4R0W6 | 7240 |
| B4HZI5 | 7238 |
| B4PPH0 | 7245 |
| B4JSA2 | 7222 |
| B7PJZ1 | 6945 |
| Q1HQT4 | 7159 |
| Q29C92 | 46245 |
| B4GNF8 | 7234 |
| B4KC31 | 7230 |
| B4NLD5 | 7260 |
| B4MB98 | 7244 |
| A8PCE7 | 6279 |
| P34604 | 6239 |
| A5DAR0 | 4929 |
| Q6BKH5 | 4959 |
| B6K4W4 | 402676 |
| A3LWH5 | 4924 |
| Q59S48 | 5476 |
| Q6CBJ8 | 4952 |
| Q86E66 | 6182 |
| Q5DDN9 | 6182 |
| A5E767 | 379508 |
| Q9USP0 | 4896 |
| P14741 | 4932 |
| B5VMH4 | 545124 |
| B3LRB0 | 285006 |

| symbol EIF2B1 | |
|---|---|
| A6ZZZ0 | 307796 |
| Q8TFL1 | 5478 |
| A7TNT2 | 436907 |
| Q754J0 | 33169 |
| Q6CK30 | 28985 |
| A8PX87 | 425265 |
| Q0CF06 | 341663 |
| Q0U2M2 | 13684 |
| A2RAX1 | 425011 |
| Q2UE99 | 5062 |
| Q1DKM9 | 5501 |
| Q5BH13 | 162425 |
| Q4WV99 | 5085 |
| A7ESN9 | 325569 |
| Q4P9P2 | 5270 |
| B2WGX9 | 426418 |
| B6HIV9 | 500485 |
| A1DE56 | 331117 |
| A1CB87 | 5057 |
| B8NH33 | 332952 |
| B8LTE3 | 441959 |
| B6Q5K4 | 441960 |
| A8NFI4 | 240176 |
| B0Y0X9 | 451804 |
| Q5KKV3 | 5207 |
| Q55VD2 | 5207 |
| A6RS37 | 332648 |
| A6QU16 | 339724 |
| Q7RX32 | 5141 |
| B2AZE1 | 5145 |
| A4QW79 | 148305 |

| symbol Ddb1 | |
|---|---|
| Q3U1J4 | 10090 |
| Q16531 | 9606 |
| P33194 | 9534 |
| A1A4K3 | 9913 |
| Q5R649 | 9601 |
| Q9ESW0 | 10116 |
| Q805F9 | 9031 |
| Q6P6Z0 | 8355 |
| B6NP50 | 7739 |
| B6MGX7 | 7739 |
| B7PIF3 | 6945 |
| Q6PGT6 | 7955 |
| A7S0J1 | 45351 |
| Q7QC66 | 7165 |
| B0X4E5 | 7176 |
| Q17I87 | 7159 |
| Q17I86 | 7159 |
| B3P186 | 7220 |

| symbol Ddb1 | |
|---|---|
| Q9XYZ5 | 7227 |
| B4G5M0 | 7234 |
| B4PPW4 | 7245 |
| Q299R6 | 46245 |
| B3LX39 | 7217 |
| B4NG66 | 7260 |
| B4HGB4 | 7238 |
| B4R1W6 | 7240 |
| B4M6L6 | 7244 |
| B4K8H1 | 7230 |
| B4JEV4 | 7222 |
| B3RLC6 | 10228 |
| Q21554 | 6239 |
| A8PZR4 | 6279 |
| A9UM77 | 8364 |
| A9URW6 | 81824 |
| Q0UHP7 | 13684 |
| B2WGB8 | 426418 |
| Q4WM68 | 5085 |
| B0Y8E0 | 451804 |
| A1DNF2 | 331117 |
| Q2UHA6 | 5062 |
| A1CTY5 | 5057 |
| B6QNF9 | 441960 |
| B8MEU5 | 441959 |
| B8N8Z0 | 332952 |
| Q0CML9 | 341663 |
| A2QS04 | 425011 |
| Q5BFT4 | 162425 |
| B6HLE5 | 500485 |
| A6QUN8 | 339724 |
| O13807 | 4896 |
| B2B3E6 | 5145 |
| Q2H1Z3 | 38033 |
| Q7S9R0 | 5141 |
| A7ESR3 | 325569 |
| A4QTE6 | 148305 |
| A6S939 | 332648 |
| B6K4A1 | 402676 |
| B0CVD1 | 486041 |
| Q1EBM7 | 5501 |
| A8PZR5 | 6279 |
| B4DSA8 | 9606 |
| B7Z2A1 | 9606 |
| Q91YC8 | 10090 |
| B4DZP5 | 9606 |

CLUSTERS

| symbol TAF8 (TBN) | |
|---|---|
| Q7Z7C8 | 9606 |
| A7MAZ4 | 9913 |
| Q9EQH4 | 10090 |
| Q7ZYA2 | 8355 |
| Q28J24 | 8364 |
| Q5ZMS1 | 9031 |
| Q6P0T2 | 7955 |
| B5XDG9 | 8030 |
| B6LXJ0 | 7739 |
| Q4PLY8 | 6945 |
| B7PFJ3 | 6945 |
| Q174K2 | 7159 |
| B0WBV2 | 7176 |
| B0WBV1 | 7176 |
| B4H082 | 7234 |
| Q29H69 | 46245 |
| Q9VWY6 | 7227 |
| B4Q2U6 | 7245 |
| A7RU65 | 45351 |
| B3NTA7 | 7220 |
| B4L2H9 | 7230 |
| B4JXF0 | 7222 |
| B3MXU6 | 7217 |
| B4I6N7 | 7238 |
| B4M7V9 | 7244 |
| B4NQ20 | 7260 |
| B3RLM7 | 10228 |
| A8NY35 | 6279 |
| Q8WQ97 | 6239 |
| Q7YTH5 | 6239 |

| symbol PSG2 | |
|---|---|
| P11465 | 9606 |
| Q9UQ72 | 9606 |
| Q8WW91 | 9606 |
| P11464 | 9606 |
| Q00887 | 9606 |
| Q00888 | 9606 |
| Q13046 | 9606 |
| Q15235 | 9606 |
| Q15238 | 9606 |
| Q16557 | 9606 |
| Q9UQ74 | 9606 |
| A5PKV3 | 9606 |
| B2R869 | 9606 |
| B3KQL2 | 9606 |
| B4DTF1 | 9606 |
| Q00889 | 9606 |
| Q15227 | 9606 |
| Q68CR6 | 9606 |
| Q6P520 | 9606 |

| symbol PSG2 | |
|---|---|
| Q8NC17 | 9606 |
| Q96QL5 | 9606 |
| B4DTG5 | 9606 |
| B4DTI6 | 9606 |
| Q8NBY8 | 9606 |
| A8K848 | 9606 |
| A8MT77 | 9606 |
| A8MY49 | 9606 |
| B4DTI5 | 9606 |
| O75237 | 9606 |
| O75238 | 9606 |
| P31997 | 9606 |
| P40199 | 9606 |
| Q0Z7S6 | 9606 |
| Q13774 | 9606 |
| Q14002 | 9606 |
| Q53XP7 | 9606 |
| Q5UB49 | 9606 |
| Q68DM9 | 9606 |
| Q9UPK8 | 9606 |
| Q9UPK9 | 9606 |
| A6NCK4 | 9606 |
| Q13178 | 9606 |
| Q8WYY6 | 9606 |

| symbol UPF1 (RENT1) | |
|---|---|
| Q92900 | 9606 |
| Q9EPU0 | 10090 |
| Q6GNR2 | 8355 |
| Q7ZVZ4 | 7955 |
| Q4SBL0 | 99883 |
| Q98TR3 | 31033 |
| B7PZ74 | 6945 |
| Q16NZ5 | 7159 |
| B0WYS1 | 7176 |
| B4L1X0 | 7230 |
| B5DMP8 | 46245 |
| B3N1D5 | 7217 |
| B4Q1H2 | 7245 |
| B4JKW7 | 7222 |
| B3NT15 | 7220 |
| B4IL93 | 7238 |
| Q9VYS3 | 7227 |
| B4M8B6 | 7244 |
| B4NTS6 | 7240 |
| B0D7I8 | 486041 |

| symbol UPF1 (RENT1) | |
|---|---|
| B4NDR4 | 7260 |
| A8NEE6 | 240176 |
| Q4PI26 | 5270 |
| B8M268 | 441959 |
| Q6MYI2 | 5085 |
| Q8TFW3 | 5085 |
| B0XR92 | 451804 |
| A1D2B4 | 331117 |
| Q5KKH8 | 5207 |
| Q55VR1 | 5207 |
| A8QBY0 | 6279 |
| Q1DS86 | 5501 |
| Q0D0N3 | 341663 |
| B6QCK9 | 441960 |
| A1CPF6 | 5057 |
| B6H5A7 | 500485 |
| Q2UCH7 | 5062 |
| B8N676 | 332952 |
| Q5BFN4 | 162425 |
| A2QRH3 | 425011 |
| A6QTP5 | 339724 |
| A6S6M8 | 332648 |
| A4RDH7 | 148305 |
| Q9HEH1 | 5141 |
| B2WIV2 | 426418 |
| B2B731 | 5145 |
| Q0UC02 | 13684 |
| Q2GY88 | 38033 |
| B6K5B6 | 402676 |
| B3RSL3 | 10228 |
| Q09820 | 4896 |
| A7E6P6 | 325569 |
| O76512 | 6239 |
| Q5A507 | 5476 |
| B5RTZ7 | 4959 |
| A5E297 | 379508 |
| A6ZMD9 | 307796 |
| P30771 | 4932 |
| B5VPK6 | 545124 |
| B3LLX3 | 285006 |
| A7TT23 | 436907 |
| B4GY25 | 7234 |
| A5DR11 | 4929 |
| Q75DS7 | 33169 |
| Q6CW68 | 28985 |
| A8Q0S3 | 425265 |
| Q6FVZ1 | 5478 |
| Q6C803 | 4952 |
| Q4R4A7 | 9541 |
| Q8SR02 | 6035 |
| B6Q0K1 | 7739 |

CLUSTERS

| symbol CDX2 | |
|---|---|
| Q99626 | 9606 |
| Q04649 | 10036 |
| Q9ESV7 | 10116 |
| P43241 | 10090 |
| Q543L9 | 10090 |
| Q8BQI5 | 10090 |
| P79788 | 9031 |
| Q0ZD37 | 55291 |
| B7ZUR8 | 8364 |
| Q90X89 | 8364 |
| B1H2D1 | 8364 |
| Q90X88 | 8364 |
| Q90X90 | 8364 |
| A5PLE8 | 7955 |
| Q0ZDF8 | 7924 |
| A6H8J8 | 8355 |
| Q91622 | 8355 |
| Q869A1 | 114398 |
| Q17243 | 7091 |
| Q3LRS0 | 6359 |
| Q8I757 | 6465 |
| A0MIN3 | 7425 |
| Q7Z1M7 | 168635 |
| Q9XYQ3 | 7165 |
| Q16G14 | 7159 |
| Q4H3T1 | 7719 |
| A9YU93 | 178772 |
| O96714 | 7070 |
| O96715 | 7070 |
| B4MWB4 | 7260 |
| P09085 | 7227 |
| A4V0Y1 | 7227 |
| B4G734 | 7234 |
| Q29LX3 | 46245 |
| B4KEC9 | 7230 |
| A9YU92 | 92596 |
| B3MK24 | 7217 |
| B0WCI9 | 7176 |
| B4Q3H6 | 7240 |
| B4P6K2 | 7245 |
| A9YU97 | 88686 |
| A9YU96 | 88686 |
| A9YU98 | 88686 |
| B3NKZ2 | 7220 |
| B4IFF8 | 7238 |
| B8XJD7 | 286459 |
| B4JC09 | 7222 |
| B4LST5 | 7244 |
| Q65ZB9 | 6239 |
| P34766 | 6239 |
| Q86C64 | 54126 |

| symbol CDX2 | |
|---|---|
| Q2T9K7 | 8355 |
| Q32NJ4 | 8355 |
| A6QLL3 | 9913 |
| O14627 | 9606 |
| P18111 | 10090 |
| P47902 | 9606 |
| Q07424 | 10090 |
| Q90Z54 | 9031 |
| Q91542 | 8355 |
| Q9DEB6 | 9031 |
| A2T7H5 | 9600 |
| A1A513 | 9606 |
| Q78Z64 | 10090 |
| Q32NU3 | 8355 |
| Q7T0X3 | 8355 |
| A8KBT9 | 7955 |
| Q6DG63 | 7955 |
| Q6T5L3 | 8022 |
| Q90262 | 7955 |
| Q90320 | 7962 |
| P46692 | 9031 |

| symbol NLE1 | |
|---|---|
| Q9NVX2 | 9606 |
| Q5RFF8 | 9601 |
| B2GV82 | 10116 |
| Q58D20 | 9913 |
| Q8VEJ4 | 10090 |
| B1ARD5 | 10090 |
| Q3URT5 | 10090 |
| Q3TC83 | 10090 |
| Q0D2B0 | 8364 |
| Q7ZXK9 | 8355 |
| O93531 | 8355 |
| Q503R2 | 7955 |
| Q4SFU2 | 99883 |
| A8P4L3 | 6279 |
| B4JDR5 | 7222 |
| Q9VPR4 | 7227 |
| O96995 | 7227 |
| B4LTN5 | 7244 |
| Q9TYV3 | 6239 |
| B5DI07 | 46245 |
| B4G881 | 7234 |
| B4ICZ0 | 7238 |
| B4KFG7 | 7230 |
| B3N7S2 | 7220 |
| B4P2G2 | 7245 |
| B4Q658 | 7240 |
| B3MLP8 | 7217 |
| B4N0R7 | 7260 |

| symbol NLE1 | |
|---|---|
| B0WQV8 | 7176 |
| Q17DU2 | 7159 |
| Q6CHQ3 | 4952 |
| Q5KHS6 | 5207 |
| Q6FPI9 | 5478 |
| P25382 | 4932 |
| B3LUC9 | 285006 |
| A6ZTP5 | 307796 |
| A6R0X1 | 339724 |
| Q756W4 | 33169 |
| B6HTT2 | 500485 |
| Q6CU08 | 28985 |
| A1CS96 | 5057 |
| A3LYL8 | 4924 |
| Q6BUQ3 | 4959 |
| A7TG10 | 436907 |
| Q4WKM1 | 5085 |
| B0XRG3 | 451804 |
| A1D562 | 331117 |
| A5E4A7 | 379508 |
| Q2URB3 | 5062 |
| Q0CN43 | 341663 |
| B2G4U1 | 4956 |
| O74855 | 4896 |
| A2Q912 | 425011 |
| A6SK49 | 332648 |
| B6K132 | 402676 |
| B8MB26 | 441959 |
| Q4P8F4 | 5270 |
| Q5BGG9 | 162425 |
| A8PS28 | 425265 |
| B8PB85 | 561896 |
| B8PHB8 | 561896 |
| B6QE75 | 441960 |
| Q0UX06 | 13684 |
| Q2HEX4 | 38033 |
| B2W009 | 426418 |
| A4QQ33 | 148305 |
| A7EGD9 | 325569 |
| Q7SFT7 | 5141 |
| A5DKH1 | 4929 |
| A8NAE4 | 240176 |
| B2AAZ6 | 5145 |
| B8MYK8 | 332952 |
| A9V9Z8 | 81824 |
| Q1DI90 | 5501 |
| B6MJC7 | 7739 |
| B6MJB6 | 7739 |

CLUSTERS

| symbol BMP4 | |
|---|---|
| P12644 | 9606 |
| B0CM38 | 9555 |
| A7LJT9 | 9823 |
| B3RF16 | 42254 |
| B2KI82 | 59479 |
| B5FW32 | 30611 |
| Q8MJV5 | 9378 |
| Q2KJH1 | 9913 |
| A8VTF8 | 9940 |
| A6N998 | 9925 |
| Q6AYU9 | 10116 |
| Q811S3 | 10116 |
| Q06826 | 10116 |
| P21275 | 10090 |
| Q3ULR1 | 10090 |
| O46576 | 9986 |
| Q29607 | 30532 |
| Q2L6L2 | 10047 |
| Q2VEW5 | 42716 |
| Q90752 | 9031 |
| A5HMF9 | 8839 |
| A5HMF8 | 8839 |
| Q90YD6 | 8364 |
| O73818 | 8355 |
| Q91703 | 8355 |
| Q6PAF3 | 8355 |
| B5X135 | 8030 |
| Q8JIJ5 | 8128 |
| Q8JIK2 | 40171 |
| Q8JIK1 | 194657 |
| Q8JFE2 | 32488 |
| Q78DH6 | 143505 |
| Q78DH5 | 70786 |
| Q78DH4 | 68046 |
| Q78DH3 | 27763 |
| Q8JIJ8 | 303518 |
| A4UY01 | 77306 |
| Q8JIK0 | 51791 |
| Q8JIJ9 | 8153 |
| Q8JIJ3 | 8148 |
| Q8JIJ7 | 70784 |
| Q8JIJ4 | 64557 |
| Q8JIJ6 | 50897 |
| Q8JIJ2 | 8129 |
| A0MK35 | 7994 |
| O57574 | 7955 |
| O13107 | 7955 |
| Q9U418 | 7741 |
| Q9U5E8 | 63121 |
| Q869A4 | 114398 |
| O76851 | 7729 |

| symbol BMP4 | |
|---|---|
| B0WCI2 | 7176 |
| P07713 | 7227 |
| B3NA13 | 7220 |
| P91706 | 7240 |
| B4Q848 | 7240 |
| Q1WKY6 | 7245 |
| B4NWQ1 | 7245 |
| Q1WKY8 | 7233 |
| P91720 | 7244 |
| B4LUE0 | 7244 |
| B4KGU4 | 7230 |
| B4GSL3 | 7234 |
| P91699 | 46245 |
| B3N1Z7 | 7217 |
| B4I2K9 | 7238 |
| B3S0I2 | 10228 |
| A8QEZ8 | 6279 |
| O16134 | 6279 |
| O16123 | 6280 |
| Q53XC5 | 9606 |
| A5PJI9 | 9913 |
| A7SAY4 | 45351 |
| B6NVZ7 | 7739 |
| O02424 | 6239 |
| O93369 | 7955 |
| P12643 | 9606 |
| P30884 | 8355 |
| P30885 | 8355 |
| P49001 | 10116 |
| Q17JZ3 | 7159 |
| Q26974 | 7070 |
| Q497W8 | 10090 |
| Q4H3U6 | 7719 |
| Q6XDQ0 | 9031 |
| Q90YD7 | 8364 |
| A0MK34 | 7994 |
| A0MK36 | 8090 |
| B1P8C3 | 8296 |
| B3DI86 | 7955 |
| B7ZPR8 | 8355 |
| B7ZRN7 | 8355 |
| O13108 | 7955 |
| O13109 | 7955 |
| O19006 | 30532 |
| O46564 | 9986 |
| O57573 | 7955 |
| O96504 | 7739 |
| P21274 | 10090 |
| P25703 | 8355 |
| Q3V1I4 | 10090 |
| Q498L8 | 8355 |

| symbol BMP4 | |
|---|---|
| Q58E94 | 8355 |
| Q0P6N0 | 45351 |
| Q5YJC4 | 45351 |
| O76514 | 6239 |
| B4JAU3 | 7222 |
| B4MU02 | 7260 |

| symbol Klf2 | |
|---|---|
| Q60843 | 10090 |
| B2RS60 | 10090 |
| Q3V293 | 10090 |
| Q9ET58 | 10116 |
| Q9Y5W3 | 9606 |
| B4X5Z2 | 9823 |
| Q28D19 | 8364 |
| Q8QGW5 | 8355 |
| Q90XE7 | 7955 |
| Q1LXI7 | 7955 |
| Q6P3M5 | 7955 |
| Q4RWQ1 | 99883 |
| B5X170 | 8030 |
| B5X2J1 | 8030 |
| B5X3X7 | 8030 |
| B6MI74 | 7739 |
| Q4H2J1 | 7719 |
| A7YWE2 | 9913 |
| B8XIA4 | 9544 |
| O43474 | 9606 |
| Q13351 | 9606 |
| Q28BQ7 | 8364 |
| Q28D29 | 8364 |
| Q52JJ4 | 9823 |
| Q60793 | 10090 |
| Q90XS5 | 8355 |
| Q923V7 | 10116 |
| Q9DFS2 | 7955 |
| B4YSE4 | 9823 |
| Q4R355 | 9541 |
| B4YSE2 | 9823 |
| P46099 | 10090 |
| Q0P5B6 | 9913 |
| Q1RME5 | 10090 |
| Q3TZ82 | 10090 |
| Q2TAV3 | 8355 |
| Q7SZU5 | 8355 |
| Q8QGW6 | 8355 |
| Q90XE8 | 7955 |

CLUSTERS

| symbol SMAD1 | |
|---|---|
| P70340 | 10090 |
| Q6GT95 | 10090 |
| Q8C3Y6 | 10090 |
| P97454 | 10090 |
| Q8CC31 | 10090 |
| Q15797 | 9606 |
| A8KAJ0 | 9606 |
| Q1JQA2 | 9913 |
| Q864V7 | 9823 |
| Q6P7A6 | 10116 |
| P97588 | 10116 |
| Q9I962 | 93934 |
| Q56IA0 | 9031 |
| Q6DIS7 | 8364 |
| Q6PF32 | 8355 |
| Q5HZ76 | 8355 |
| P79947 | 8355 |
| Q91693 | 8355 |
| Q4RKM8 | 99883 |
| Q5PQZ2 | 7955 |
| Q9I8V2 | 7955 |
| Q7T082 | 7955 |
| Q179T8 | 7159 |
| Q7Q8L8 | 7165 |
| A5HNG5 | 155462 |
| B6N1T6 | 7739 |
| B4I2W3 | 7238 |
| B4NXE4 | 7245 |
| B3NAR3 | 7220 |
| B4MZB1 | 7260 |
| B4KEV0 | 7230 |
| P42003 | 7227 |
| Q4H2T7 | 7719 |
| B0W056 | 7176 |
| B4LU30 | 7244 |
| B5DJI3 | 46245 |
| B4GSG1 | 7234 |
| B3N202 | 7217 |
| B7Q8J0 | 6945 |
| O97044 | 7729 |
| B4JAN5 | 7222 |
| B4Q940 | 7240 |
| A8SLH6 | 79327 |
| A7RNC1 | 45351 |
| Q27W08 | 45351 |
| Q70TA7 | 6211 |
| Q810W4 | 10090 |
| Q5R6H7 | 9601 |
| Q99717 | 9606 |
| Q68DB7 | 9606 |
| Q6I9T1 | 9606 |

| symbol SMAD1 | |
|---|---|
| Q9R1V3 | 10116 |
| B1WBR0 | 10116 |
| Q56I99 | 9031 |
| Q9W7E7 | 7955 |
| A5PMC0 | 7955 |
| Q7ZU01 | 7955 |
| B5X4J3 | 8030 |
| B5LVZ1 | 10224 |
| Q8T6S2 | 6087 |
| B3RY25 | 10228 |
| Q9GQN9 | 6183 |
| Q5DBJ6 | 6182 |
| A8Q2C6 | 6279 |
| A8Q7L1 | 6279 |
| O15198 | 9606 |
| O54835 | 10116 |
| Q56I98 | 9031 |
| Q6DIZ6 | 8364 |
| Q6PF37 | 8355 |
| Q9JIW5 | 10090 |
| A2PZA6 | 7668 |
| A7S9L0 | 45351 |
| A8K4B6 | 9606 |
| B0WCK9 | 7176 |
| B1B1A2 | 10090 |
| B2XS64 | 50426 |
| B3NWK5 | 7220 |
| B6LVE9 | 7739 |
| B6MYD0 | 7739 |
| B7Z4Z5 | 9606 |
| B7Z5N5 | 9606 |
| O70436 | 10116 |
| P84022 | 9606 |
| P84023 | 9031 |
| P84024 | 9823 |
| P84025 | 10116 |
| Q06BS0 | 7959 |
| Q06BS1 | 7959 |
| Q07G47 | 8364 |
| Q08DE0 | 9913 |
| Q15796 | 9606 |
| Q1W668 | 9913 |
| Q2A658 | 6211 |
| Q32N71 | 8355 |
| Q4H2T5 | 7719 |
| Q4H2T6 | 7719 |
| Q53XR6 | 9606 |
| Q5R7C0 | 9601 |
| Q5U4Q3 | 8364 |
| Q62432 | 10090 |
| Q66HX9 | 7955 |

| symbol SMAD1 | |
|---|---|
| Q68EP5 | 8364 |
| Q6IRR4 | 8355 |
| Q6QAN7 | 7957 |
| Q6QAN8 | 7957 |
| Q7ZU35 | 7955 |
| Q8AY15 | 7955 |
| Q8AY16 | 7955 |
| Q8BUN5 | 10090 |
| Q8T8C9 | 7729 |
| Q8UWF3 | 9031 |
| Q90YE5 | 8355 |
| Q91912 | 8355 |
| Q9I9P9 | 7955 |
| B0WBS4 | 7176 |
| B3RI80 | 10228 |
| Q02330 | 6239 |
| A2A2Y6 | 9606 |
| Q0VER3 | 10090 |
| Q3UVC6 | 10090 |
| Q1LZG1 | 9913 |
| Q4T7H2 | 99883 |
| Q5TBA1 | 9606 |
| Q5XJE0 | 7955 |
| Q6A3P7 | 7955 |
| Q8QGG4 | 8355 |
| Q8QGG5 | 8355 |
| Q8QGG6 | 8355 |
| Q91913 | 8355 |
| Q98TU1 | 8355 |
| Q06AL5 | 9913 |

CLUSTERS

| symbol Cer1 | |
|---|---|
| O55233 | 10090 |
| B0BN87 | 10116 |
| Q3SY34 | 9606 |
| O95813 | 9606 |
| Q9PWB0 | 9031 |
| Q9PUK2 | 9031 |
| P70041 | 8355 |
| Q07G34 | 8364 |
| B7ZTZ7 | 8364 |
| A8E7M9 | 7955 |
| Q76C29 | 7955 |
| Q76C27 | 8255 |
| Q76C28 | 31033 |
| A2ADM9 | 10090 |

| symbol Cga | |
|---|---|
| P01216 | 10090 |
| Q3MU80 | 10032 |
| Q2L4U1 | 10029 |
| Q2HWE5 | 10044 |
| Q9ERG5 | 10036 |
| Q9ERG4 | 35658 |
| Q9ERJ6 | 10047 |
| P11962 | 10116 |
| Q6P509 | 10116 |
| P68268 | 9337 |
| Q6YNX4 | 13616 |
| P68267 | 9321 |
| Q9BDI8 | 74533 |
| P01219 | 9823 |
| Q8HZS0 | 9649 |
| P07474 | 9986 |
| Q52R91 | 9685 |
| Q9XSW8 | 9615 |
| Q9ERG3 | 79202 |
| Q8WN20 | 9646 |
| P01217 | 9913 |
| Q19PY8 | 30521 |
| Q8WMR3 | 9863 |
| P01218 | 9940 |
| Q9GL36 | 89462 |
| Q8WMW8 | 9925 |
| B5TXE0 | 9860 |
| P51499 | 9483 |
| Q3YC03 | 39432 |
| Q28365 | 9793 |
| Q8JJB6 | 260615 |
| O46642 | 9790 |
| Q9BEH3 | 9541 |
| P22762 | 9544 |
| Q3HRV5 | 37293 |

| symbol Cga | |
|---|---|
| Q1EPR1 | 10160 |
| P01220 | 9796 |
| P68241 | 9103 |
| P68242 | 93934 |
| B2YI99 | 8845 |
| Q9JK68 | 10141 |
| Q8JIE9 | 128390 |
| Q8JJB7 | 8508 |
| Q9DGA6 | 7892 |
| P37036 | 9767 |
| P25329 | 9755 |
| P01215 | 9606 |
| Q6I9S8 | 9606 |
| Q96QJ4 | 9606 |
| Q6INB1 | 8355 |
| P80665 | 8801 |
| Q90YF0 | 7830 |
| B2ZFP8 | 61970 |
| B2ZFP7 | 61970 |
| Q8JJB5 | 118850 |
| Q5YFR9 | 7902 |
| P37037 | 13095 |
| A7XUG4 | 7965 |
| P30983 | 7959 |
| A7XUG2 | 7959 |
| B8Y6D6 | 7939 |
| P27794 | 7936 |
| Q9YGP3 | 7998 |
| Q75NP8 | 7937 |
| Q75N55 | 8402 |
| P18857 | 7962 |
| P01221 | 7962 |
| Q90WD7 | 27689 |
| Q5IAT0 | 211491 |
| Q5IAS9 | 211491 |
| Q5I9C7 | 211491 |
| Q5IAT1 | 211491 |
| Q5D1X7 | 211488 |
| Q5D1X6 | 211488 |
| Q5D1X8 | 211488 |
| Q5D1X9 | 211488 |
| P53542 | 13013 |
| Q5RT66 | 7957 |
| Q5RT67 | 7957 |
| Q14DX5 | 175797 |
| Q6QX43 | 7955 |
| B9EMI1 | 8030 |
| Q6TM06 | 8030 |
| Q91370 | 8020 |
| Q91371 | 8020 |
| P80051 | 8400 |

| symbol Cga | |
|---|---|
| P12836 | 7946 |
| Q90ZY3 | 13489 |
| Q9DG94 | 8022 |
| P69062 | 74940 |
| P69063 | 8018 |
| Q6X4V6 | 188791 |
| A6BMG6 | 42892 |
| B5LSW8 | 229072 |
| Q91119 | 34816 |
| Q7SYG7 | 232417 |
| Q9DEH2 | 143350 |
| P30970 | 8177 |
| Q71SH0 | 8175 |
| A5Z1E4 | 72011 |
| A7UDN7 | 8090 |
| Q8AXA3 | 43700 |
| Q8AXA4 | 205852 |
| Q90W29 | 8255 |
| Q8AUU2 | 94232 |
| A5JVA3 | 323802 |
| P37204 | 8241 |
| Q7T2Y4 | 8128 |
| A8J4H5 | 98377 |
| Q56TL7 | 214486 |
| Q801A1 | 215347 |
| Q2EGA1 | 219752 |
| Q4TUC5 | 103715 |
| P47744 | 8078 |
| B2KS58 | 28829 |
| Q90W18 | 8267 |
| B0VXR7 | 8049 |
| A1ILL6 | 146921 |
| A2AVN3 | 10090 |

| symbol AFP | |
|---|---|
| P02771 | 9606 |
| P28050 | 9595 |
| Q28789 | 9598 |
| Q8MJU5 | 9615 |
| Q8MJ76 | 9823 |
| P49066 | 9796 |
| Q7TSF3 | 9995 |
| Q3SZ57 | 9913 |
| P02772 | 10090 |
| Q8BK56 | 10090 |
| Q8BK65 | 10090 |
| P02773 | 10116 |
| Q4QR90 | 10116 |
| B4DMX4 | 9606 |
| B4DMW9 | 9606 |

CLUSTERS

| symbol ATP6AP2 | |
|---|---|
| O75787 | 9606 |
| Q5R563 | 9601 |
| Q4R500 | 9541 |
| P81134 | 9913 |
| Q1XID4 | 10090 |
| Q9CYN9 | 10090 |
| A2BDN5 | 10090 |
| Q6AXS4 | 10116 |
| Q1XIH7 | 9031 |
| Q5ZMF2 | 9031 |
| B5X2F3 | 8030 |
| Q5M8F1 | 8364 |
| Q6PBY2 | 7955 |
| Q7T0S3 | 8355 |
| Q4V855 | 8355 |
| Q4RWA5 | 99883 |
| B6M7F9 | 7739 |
| B6NBN0 | 7739 |
| Q29CF3 | 46245 |
| B4GN51 | 7234 |
| B3M1L3 | 7217 |
| B3M161 | 7217 |
| Q9VHG4 | 7227 |
| B4HKG4 | 7238 |
| B4PUL1 | 7245 |
| B4K620 | 7230 |
| B4KU35 | 7230 |
| A7SQ62 | 45351 |

| symbol Pou5f1 (OCT4) | |
|---|---|
| P20263 | 10090 |
| Q6MG27 | 10116 |
| A0MPW0 | 30630 |
| Q5TM49 | 9544 |
| B8XIA3 | 9544 |
| Q0KKP8 | 9541 |
| Q01860 | 9606 |
| Q06416 | 9606 |
| Q9TSV5 | 9823 |
| Q7YR49 | 9598 |
| A2ICN2 | 9986 |
| O97552 | 9913 |
| A7X5W5 | 9258 |
| Q5J1Q2 | 8296 |
| B7ZQA9 | 8355 |
| Q03917 | 8355 |
| Q6DVF4 | 8090 |
| B3DM50 | 8364 |
| B3DM25 | 8364 |
| B3DM23 | 8364 |

| symbol GJB3 | |
|---|---|
| O75712 | 9606 |
| Q58D78 | 9913 |
| P25305 | 10116 |
| P28231 | 10090 |
| A2A8M5 | 10090 |
| Q6PYT9 | 10036 |
| Q6GQC4 | 8355 |
| Q8QFX8 | 8355 |
| A8WGZ8 | 8364 |
| Q567J2 | 7955 |
| Q8AWR5 | 7955 |
| B3DIR8 | 7955 |
| B2R790 | 9606 |
| A1YRJ5 | 9606 |
| A2A8M6 | 10090 |
| B3KQ82 | 9606 |
| O95377 | 9606 |
| P28232 | 10116 |
| P36380 | 10116 |
| Q02738 | 10090 |
| Q02739 | 10090 |
| Q0H791 | 8355 |
| Q4RNY0 | 99883 |
| Q542M8 | 10090 |
| Q6PEY0 | 9606 |
| Q7SXS1 | 8355 |
| Q8C677 | 10090 |
| Q8QGJ5 | 31033 |
| Q91XA4 | 10090 |
| Q9NTQ9 | 9606 |

| symbol THY1 | |
|---|---|
| P04216 | 9606 |
| Q5R508 | 9601 |
| O62643 | 9544 |
| Q9WUR5 | 10141 |
| Q3SX33 | 9913 |
| P01830 | 10116 |
| P01831 | 10090 |
| Q53YX2 | 10090 |
| Q7T252 | 9031 |
| Q07212 | 9031 |
| B5G3M1 | 59729 |
| B0YJA4 | 9606 |
| Q8AV81 | 7955 |
| A2BFD8 | 7955 |
| A7MCJ5 | 7955 |
| A9UME7 | 8364 |
| Q3B751 | 7955 |

| symbol Wnt6 | |
|---|---|
| P22727 | 10090 |
| Q80ZM9 | 10090 |
| Q9Y6F9 | 9606 |
| Q8N2E5 | 9606 |
| Q3C2H6 | 9031 |
| B0FK91 | 8355 |
| B0FK92 | 8355 |
| Q4RNC9 | 99883 |
| B6L7E8 | 7739 |
| B0WGM5 | 7176 |
| B4G844 | 7234 |
| Q29N83 | 46245 |
| B4JQN3 | 7222 |
| Q9VM26 | 7227 |
| B3MK98 | 7217 |
| B4KKC9 | 7230 |
| B4LS42 | 7244 |
| B4MZW3 | 7260 |
| B4NZL8 | 7245 |
| B3N6B6 | 7220 |

| symbol Gjc1 | |
|---|---|
| P28229 | 10090 |
| Q6R4A8 | 10029 |
| Q6PYT3 | 10036 |
| A4GG66 | 10116 |
| P36383 | 9606 |
| B3KW68 | 9606 |
| A4GVD1 | 9823 |
| P28228 | 9615 |
| Q2HJ66 | 9913 |
| P18861 | 9031 |
| Q66HV7 | 7955 |
| Q0V9Q2 | 8364 |
| Q7ZXS7 | 8355 |
| Q8QFX7 | 8355 |
| Q1HG92 | 61084 |
| Q1HG91 | 61084 |
| A2AUA9 | 10090 |
| Q6NZH5 | 8364 |
| B0UYC0 | 7955 |
| Q803S5 | 7955 |
| Q92052 | 7955 |

| symbol C7orf36 (GK003) | |
|---|---|
| Q9NRH1 | 9606 |
| Q6TXH1 | 10116 |

CLUSTERS

| symbol FGF4 | |
|---|---|
| P08620 | 9606 |
| P48803 | 9913 |
| Q542N0 | 10090 |
| P11403 | 10090 |
| Q8R5L6 | 10116 |
| B7U3X7 | 8364 |
| P48806 | 8355 |
| P48805 | 8355 |
| P48804 | 9031 |
| Q9YH31 | 8316 |
| Q9DFC9 | 7955 |
| A6H6W8 | 9913 |
| A7SG69 | 45351 |
| A7SI24 | 45351 |
| A8P371 | 6279 |
| A8Q0I7 | 6279 |
| B6KY19 | 7739 |
| B6M7K3 | 7739 |
| O15520 | 9606 |
| O35565 | 10090 |
| O35622 | 10090 |
| O42407 | 9031 |
| O43320 | 9606 |
| O54769 | 10116 |
| O60258 | 9606 |
| O76093 | 9606 |
| O88182 | 10116 |
| O89101 | 10090 |
| O95750 | 9606 |
| P03968 | 9913 |
| P03969 | 9913 |
| P05230 | 9606 |
| P09038 | 9606 |
| P10767 | 9606 |
| P11487 | 9606 |
| P12034 | 9606 |
| P12226 | 8355 |
| P13109 | 10116 |
| P15655 | 10090 |
| P15656 | 10090 |
| P19596 | 9031 |
| P21658 | 10090 |
| P21781 | 9606 |
| P31371 | 9606 |
| P36363 | 10090 |
| P36364 | 10116 |
| P37237 | 10090 |
| P48798 | 13616 |
| P48800 | 9031 |
| P48801 | 9031 |
| P48802 | 7955 |

| symbol FGF4 | |
|---|---|
| P48807 | 10116 |
| P54130 | 10090 |
| P55075 | 9606 |
| P61148 | 10090 |
| P61149 | 10116 |
| P61150 | 10116 |
| P61328 | 9606 |
| P61329 | 10090 |
| P63075 | 10090 |
| P63076 | 10116 |
| P70378 | 10090 |
| P70379 | 10090 |
| P70492 | 10116 |
| P79150 | 9615 |
| Q02195 | 10116 |
| Q0VG15 | 10090 |
| Q11184 | 6239 |
| Q197G3 | 9031 |
| Q20FD0 | 9615 |
| Q28DZ3 | 8364 |
| Q2LGG1 | 7955 |
| Q5KRA4 | 9031 |
| Q5M7N7 | 8364 |
| Q5PRC3 | 7955 |
| Q5TKR1 | 7955 |
| Q641J3 | 8364 |
| Q66IF7 | 8364 |
| Q6DFL4 | 8355 |
| Q6GLR6 | 8355 |
| Q6GLX1 | 8355 |
| Q6NZS6 | 7955 |
| Q6SJP8 | 7955 |
| Q76LI5 | 10116 |
| Q7ZZN4 | 9031 |
| Q8AY90 | 7955 |
| Q8I6J3 | 7719 |
| Q8I6J6 | 7719 |
| Q8R5L5 | 10116 |
| Q8R5L7 | 10116 |
| Q8R5L8 | 10116 |
| Q8R5L9 | 10116 |
| Q8VCY9 | 10090 |
| Q8VI79 | 10116 |
| Q8VI80 | 10116 |
| Q8VI81 | 10116 |
| Q8VI82 | 10116 |
| Q90722 | 9031 |
| Q92913 | 9606 |
| Q92914 | 9606 |
| Q92915 | 9606 |
| Q95L12 | 9823 |

| symbol FGF4 | |
|---|---|
| Q9DDN0 | 9031 |
| Q9EPC2 | 10090 |
| Q9ERW3 | 10116 |
| Q9ESL8 | 10090 |
| Q9ESL9 | 10090 |
| Q9ESS2 | 10090 |
| Q9EST9 | 10116 |
| Q9GZV9 | 9606 |
| Q9HCT0 | 9606 |
| Q9I950 | 9031 |
| Q9IAI3 | 9031 |
| Q9IAI5 | 9031 |
| Q9JJN1 | 10090 |
| Q9NP95 | 9606 |
| Q9NSA1 | 9606 |
| Q9W6A1 | 9031 |
| A0AUQ1 | 7955 |
| A6P7H5 | 8355 |
| A6P7H6 | 8355 |
| A6QPP3 | 9913 |
| A8K1P5 | 9606 |
| B1AK18 | 9606 |
| B2R976 | 9606 |
| B3DHZ8 | 7955 |
| B7U3Y0 | 8364 |
| B7U3Y1 | 8364 |
| B7Z4M7 | 9606 |
| B7Z8N0 | 9606 |
| B8Y4H7 | 8364 |
| B8Y4H8 | 8364 |
| B8Y4H9 | 8364 |
| O89096 | 10090 |
| P70377 | 10090 |
| Q0IHY3 | 8364 |
| Q3UR31 | 10090 |
| Q4RP64 | 99883 |
| Q4RVA8 | 99883 |
| Q5R5C0 | 9601 |
| Q5RDS9 | 9601 |
| Q5TKR2 | 7955 |
| Q5TKR3 | 7955 |
| Q5TKR4 | 7955 |
| Q5U3Q2 | 7955 |
| Q794I6 | 10116 |
| Q9W6A2 | 9031 |
| B5LX82 | 8355 |
| B7ZSQ9 | 8364 |
| Q07G49 | 8364 |
| Q5R2I6 | 13735 |
| Q6RX23 | 9863 |
| Q8QG59 | 8296 |

CLUSTERS

| symbol FGF4 | |
|---|---|
| Q95K97 | 9541 |
| Q9N1B9 | 9940 |
| Q790L8 | 10090 |
| A2RTU5 | 10090 |
| B7ZLG4 | 9606 |
| B7ZMS7 | 10090 |
| Q0VF19 | 10090 |
| Q8C471 | 10090 |
| Q0VCA0 | 9913 |
| Q5SQB2 | 10090 |
| Q5TKR7 | 7955 |
| Q5TKR8 | 7955 |
| A8K147 | 9606 |
| B0CM87 | 9555 |
| B0KW92 | 9483 |
| B1MTE5 | 9523 |
| B2KI41 | 59479 |
| B2R5T0 | 9606 |
| B3DGE3 | 7955 |
| B3EX47 | 42254 |
| B4USY8 | 30611 |
| B7NZB1 | 9986 |
| B7U3X6 | 8364 |
| P34004 | 10036 |
| Q5NVQ3 | 9601 |
| Q6ZWS1 | 10090 |
| Q7M303 | 9940 |
| A4LBB8 | 9606 |
| P20003 | 9940 |
| P78443 | 9606 |
| Q4JH23 | 9940 |
| Q541T2 | 10090 |
| Q8QFR9 | 31033 |
| B7U3X8 | 8364 |
| Q5MFQ8 | 13489 |
| Q5TLE1 | 7955 |
| Q8AXA1 | 7955 |
| Q9YGD8 | 8022 |
| B3DHR1 | 7955 |
| B5X6L5 | 8030 |
| B7ZPF9 | 8355 |
| P05524 | 10090 |
| P36386 | 8355 |
| Q3KQ53 | 8355 |
| A0MTF4 | 9913 |
| A2AXN8 | 9685 |
| Q8NF90 | 9606 |
| A6Y7S4 | 42716 |
| B7ZPK6 | 8355 |
| Q5FXK5 | 8022 |
| Q90Y92 | 8330 |

| symbol FGF4 | |
|---|---|
| Q925A3 | 10090 |
| B2MVW3 | 9940 |
| P48808 | 9940 |
| Q4KL95 | 8355 |
| Q544I6 | 10090 |
| Q5D0X0 | 9860 |
| Q5RAY8 | 9601 |
| Q8C386 | 10090 |
| Q9N198 | 9823 |
| A8K427 | 9606 |
| A9CSE5 | 8410 |
| B2RPH5 | 9606 |
| B7UCP9 | 8364 |
| B7ZQK4 | 8355 |
| Q059W0 | 10090 |
| Q0KJ02 | 8090 |
| Q0KJ03 | 8090 |
| Q2HXK8 | 7955 |
| Q2LJ24 | 7955 |
| Q38PL0 | 7955 |
| Q3ZFI5 | 42716 |
| Q499I9 | 10090 |
| Q91875 | 8355 |
| Q9PVY1 | 8355 |
| B3DJ36 | 7955 |
| B6EBS1 | 7959 |
| B7TIR5 | 38577 |
| O42278 | 7955 |
| O57341 | 7955 |
| Q0PJT5 | 7994 |
| Q3V279 | 10090 |
| Q4R0X9 | 9031 |
| Q805B2 | 7955 |
| Q80ZL6 | 10090 |
| Q8AXC5 | 8355 |
| Q8MJV6 | 9378 |
| Q9DE51 | 8296 |
| A1A514 | 9606 |
| A1A515 | 9606 |
| A2CF52 | 7955 |
| B8Q2W1 | 42716 |
| Q0E9Y8 | 7955 |
| A4IIV2 | 8364 |
| B1AU20 | 10090 |
| B5X194 | 8030 |
| Q0P4D2 | 7955 |
| Q5E9M0 | 9913 |
| Q5F291 | 10090 |
| Q5R8V8 | 9601 |
| Q9IAI7 | 9031 |
| B7Z1C3 | 9606 |

| symbol FGF4 | |
|---|---|
| Q3KNZ6 | 10090 |
| Q1XG75 | 7955 |
| Q4VBV7 | 7955 |
| Q8I6J1 | 51511 |
| B2RPH4 | 9606 |
| O60371 | 9606 |
| Q8N683 | 9606 |
| Q0VBJ8 | 10090 |
| Q3U1V5 | 10090 |

| symbol STAT3 | |
|---|---|
| P40763 | 9606 |
| P42227 | 10090 |
| A2A5D1 | 10090 |
| P52631 | 10116 |
| Q19S50 | 9823 |
| P61635 | 9913 |
| Q5R5Q7 | 9601 |
| Q6DV79 | 9031 |
| Q9PVX8 | 8355 |
| Q6NV46 | 7955 |
| O93599 | 7955 |
| Q6DVF3 | 8090 |
| Q6GUE7 | 8090 |
| Q90Y16 | 47145 |
| O13133 | 8022 |
| Q7ZXK3 | 8355 |
| A0T2M6 | 215402 |
| A8K7B8 | 9606 |
| B2BF21 | 8255 |
| B4DNP0 | 9606 |
| B4DVR6 | 9606 |
| B5BTZ6 | 9606 |
| B7ZA24 | 9606 |
| B7ZC17 | 10090 |
| B7ZC18 | 10090 |
| Q08D02 | 8364 |
| Q32LP6 | 9913 |
| Q3U5Q4 | 10090 |
| Q3U6S9 | 10090 |
| Q3ULI4 | 10090 |
| Q5RF79 | 9601 |
| Q6GU23 | 10090 |
| Q8JGN0 | 8355 |

CLUSTERS

| symbol SOX2 | |
|---|---|
| P48431 | 9606 |
| P48430 | 9031 |
| B8XIA2 | 9544 |
| P54231 | 9940 |
| A2VDX8 | 9913 |
| B1PXF9 | 9823 |
| B1Q0D1 | 9823 |
| Q60I23 | 10090 |
| P48432 | 10090 |
| Q6NVN0 | 8364 |
| B6VA41 | 9796 |
| O42569 | 8355 |
| Q6DFL8 | 8355 |
| B5X176 | 8030 |
| Q6P0E1 | 7955 |
| Q6WNU1 | 31033 |
| Q4T665 | 99883 |
| B5LSX2 | 229072 |
| Q9PT76 | 8090 |
| A2I8A8 | 7957 |
| Q38KZ5 | 94232 |
| Q06B63 | 8187 |
| A5Z1E1 | 72011 |
| Q0Q579 | 7739 |
| Q7YTD4 | 10224 |
| Q9Y0D7 | 7668 |
| Q6QNM5 | 7635 |
| Q6QNM6 | 7634 |
| Q1HTN3 | 62006 |
| Q3S381 | 45351 |
| Q86SB8 | 7729 |
| B7QK34 | 6945 |
| B4ZYS7 | 45264 |
| Q21305 | 6239 |
| Q8IG08 | 6239 |
| A8PJJ8 | 6279 |
| B4QJY1 | 7240 |
| B4QJY2 | 7240 |
| Q17NV3 | 7159 |
| B0WKD7 | 7176 |
| B4PI04 | 7245 |
| B4HHC5 | 7238 |
| B3ND15 | 7220 |
| A2AM37 | 10090 |
| B7SZV3 | 10116 |
| O00570 | 9606 |
| O57401 | 9031 |
| O95416 | 9606 |
| P41225 | 9606 |
| Q0Q578 | 7739 |
| Q17MW0 | 7159 |

| symbol SOX2 | |
|---|---|
| Q2Z1R2 | 7955 |
| Q4H2S2 | 7719 |
| Q4H2S3 | 7719 |
| Q68FA4 | 8364 |
| Q6DGL6 | 7955 |
| Q6EJB7 | 7955 |
| Q6RVD7 | 7955 |
| Q80XF2 | 10090 |
| Q9PSV6 | 9031 |
| Q9W7R6 | 9031 |
| Q9Y0D8 | 7668 |
| Q9Y651 | 9606 |
| A7VKI3 | 8410 |
| A9JSZ3 | 7955 |
| B2R6J0 | 9606 |
| P48433 | 9031 |
| P53784 | 10090 |
| P55863 | 8355 |
| Q4RP71 | 99883 |
| Q5FWM3 | 8355 |
| Q6WNU0 | 31033 |
| Q80XF1 | 10090 |
| A4GU08 | 8128 |
| A4GU09 | 47969 |
| A4QNG3 | 8364 |
| B0ZTE2 | 8355 |
| B2RAC0 | 9606 |
| B5M209 | 10224 |
| B6DXB2 | 7741 |
| B7SZV1 | 9925 |
| B7SZV2 | 9913 |
| P61259 | 9541 |
| Q04892 | 10090 |
| Q32PP9 | 7955 |
| Q3UUP3 | 10090 |
| Q4SV91 | 99883 |
| Q6WNT7 | 31033 |
| Q6WNT8 | 31033 |
| Q7SZS1 | 7955 |
| Q804S0 | 7955 |
| Q9PVF6 | 7955 |
| Q9YH21 | 7955 |
| P53783 | 10090 |
| A2TED2 | 8355 |
| A2TED3 | 8364 |
| B0UY96 | 7955 |
| Q2PG84 | 8355 |
| Q6WNU3 | 31033 |
| A8WFX7 | 7955 |
| Q6I753 | 8330 |
| Q6WNU2 | 31033 |

| symbol SOX2 | |
|---|---|
| B0ZTE1 | 8355 |
| B9EJU0 | 10090 |
| Q5TBS1 | 9606 |
| Q6WNT6 | 31033 |
| Q811W0 | 10090 |
| Q9W7R5 | 9031 |
| B5G687 | 7668 |
| B5G688 | 7668 |
| B5G689 | 7668 |
| B5G693 | 7668 |
| B5G695 | 7668 |
| B5G696 | 7668 |
| B5G699 | 7668 |
| B5G6A0 | 7668 |
| B5G6A4 | 7668 |
| B5G6A6 | 7668 |
| B5G6A7 | 7671 |
| B5G6A8 | 7671 |
| B5G6B0 | 7671 |
| B5G6B3 | 7671 |
| B5G6B9 | 7671 |
| B5G6C0 | 7671 |
| B5G6C1 | 7671 |
| B5G6C2 | 7671 |
| B5G6C4 | 7671 |
| B5G6C5 | 7671 |
| B5G6C6 | 7670 |
| B5G6C7 | 7670 |
| B5G6C8 | 7670 |
| B5G6C9 | 7670 |
| B5G6D2 | 7670 |
| B5G6D7 | 7670 |
| B5G6E0 | 7670 |
| B5G6E3 | 7670 |
| B5G6E5 | 7670 |
| B5G6E6 | 7665 |
| B5G6E7 | 7665 |
| B5G6E9 | 7665 |
| B5G6F1 | 7665 |
| B5G6F2 | 7665 |
| Q6QNM7 | 7635 |
| Q6QNM8 | 7634 |

CLUSTERS

| symbol Ctnnb1 | |
|---|---|
| Q02248 | 10090 |
| Q76LW0 | 10047 |
| P35222 | 9606 |
| A8K1L7 | 9606 |
| B5BU28 | 9606 |
| Q5R5L8 | 9601 |
| Q0VCX4 | 9913 |
| Q8WNW4 | 9823 |
| B6V8E6 | 9615 |
| B1MV73 | 9796 |
| Q9WU82 | 10116 |
| Q5R2I4 | 13735 |
| B5U453 | 8839 |
| O42486 | 9031 |
| B3F375 | 8843 |
| Q4R3D3 | 9541 |
| Q63ZT4 | 8355 |
| P26233 | 8355 |
| Q7ZX35 | 8355 |
| A1A5I6 | 8355 |
| Q28GC2 | 8364 |
| B5U307 | 7957 |
| Q7T192 | 7957 |
| Q7ZU14 | 7955 |
| Q90424 | 7955 |
| B6MTY7 | 7739 |
| Q4U478 | 7739 |
| Q75WV7 | 7741 |
| B5THL1 | 10224 |
| A5YRN6 | 6359 |
| A1L024 | 6500 |
| P35224 | 6431 |
| P35223 | 7673 |
| B4N1V6 | 7260 |
| Q29I35 | 46245 |
| B4M333 | 7244 |
| B4JWY3 | 7222 |
| P18824 | 7227 |
| B3P8S9 | 7220 |
| B3MXH2 | 7217 |
| Q02453 | 7370 |
| Q76CZ7 | 6999 |
| B4Q1K8 | 7245 |
| Q17GS9 | 7159 |
| Q9NL44 | 7719 |
| Q4H3U7 | 7719 |
| O76152 | 51511 |
| Q7QHW5 | 7165 |
| B0WHS4 | 7176 |
| A4UAH1 | 6096 |
| B4L5M8 | 7230 |

| symbol Ctnnb1 | |
|---|---|
| B4I9K6 | 7238 |
| A8Q356 | 6279 |
| Q589S3 | 6161 |
| B2BLN1 | 54126 |
| Q3UZT7 | 10090 |
| Q8WRT5 | 34590 |
| A9YWT3 | 6221 |
| Q75WU5 | 114398 |
| O61229 | 7654 |
| B7SFY0 | 317513 |
| Q6LCQ8 | 6087 |
| Q25100 | 6085 |
| O44326 | 6239 |
| Q10953 | 6239 |
| Q18825 | 6239 |
| B4DGU4 | 9606 |
| B5X1P2 | 8030 |
| B5X4L1 | 8030 |
| Q8JID2 | 7955 |
| A2A4H7 | 10090 |
| B4DL06 | 9606 |
| B4DSW9 | 9606 |
| P14923 | 9606 |
| P70565 | 10116 |
| Q02257 | 10090 |
| Q6P0K8 | 10116 |
| Q8SPJ1 | 9913 |
| Q8WNW3 | 9823 |
| Q9PVF7 | 7955 |
| A8X811 | 6238 |

| symbol TEAD4 | |
|---|---|
| Q15561 | 9606 |
| B7SFP7 | 9823 |
| Q62296 | 10090 |
| P48984 | 9031 |
| B4IKD9 | 7238 |
| Q058U0 | 7227 |
| P30052 | 7227 |
| A8JUY9 | 7227 |
| Q8IR25 | 7227 |
| Q8SZ20 | 7227 |
| B4M2R1 | 7244 |
| B4L4E3 | 7230 |
| B4PX75 | 7245 |
| B4JJP7 | 7222 |
| A8PE41 | 6279 |
| B3M5D2 | 7217 |
| B6VBH7 | 135651 |
| A7MBZ7 | 7955 |
| A7SHJ8 | 45351 |

| symbol TEAD4 | |
|---|---|
| A8CSN2 | 8355 |
| A8JUY5 | 7227 |
| A9V2G2 | 81824 |
| B3RSV7 | 10228 |
| B7SFP5 | 9823 |
| P28347 | 9606 |
| P30051 | 10090 |
| Q29CR3 | 46245 |
| Q2VI02 | 9823 |
| Q4H2U7 | 7719 |
| Q641H5 | 8355 |
| Q7SXX3 | 7955 |
| Q99594 | 9606 |
| A3KN29 | 9913 |
| A9JSZ6 | 7955 |
| B4DTJ6 | 9606 |
| P70210 | 10090 |
| Q4V953 | 7955 |
| Q71U35 | 9606 |
| Q90701 | 9031 |
| Q9Z0P8 | 10090 |
| Q6MZR9 | 9606 |
| B7SFP6 | 9823 |
| Q05B82 | 9913 |
| Q3UFP5 | 10090 |
| Q3USK5 | 10090 |
| Q6PAQ8 | 10090 |
| Q80UL2 | 10090 |
| Q8NA25 | 9606 |
| B3NXJ4 | 7220 |
| A2BDD5 | 10090 |
| B5DF69 | 10116 |
| B7SFP8 | 9823 |
| P48301 | 10090 |
| Q15562 | 9606 |
| Q3UMJ5 | 10090 |
| Q3UPV9 | 10090 |
| Q3V262 | 10090 |
| Q4SIA4 | 99883 |
| B4HC05 | 7234 |

CLUSTERS

| symbol Nanog | |
|---|---|
| Q5TM83 | 57486 |
| Q80Z64 | 10090 |
| A2RS90 | 10090 |
| Q0QJG1 | 10096 |
| Q0QJG0 | 10091 |
| A8QWW8 | 10116 |
| B5B3P3 | 30630 |
| B5B3P4 | 30630 |
| Q9H9S0 | 9606 |
| A8K4D1 | 9606 |
| Q6NSW7 | 9606 |
| Q8N7R0 | 9606 |
| Q3LTE0 | 9598 |
| A2T763 | 9598 |
| Q5ICG3 | 9925 |
| Q5TM84 | 9541 |
| Q95KD6 | 9541 |
| Q4JM65 | 9913 |
| Q1W1Y4 | 9823 |
| A1YG92 | 9597 |
| A7Y7W3 | 9031 |
| A8Q4B2 | 6279 |
| A8PZL2 | 6279 |
| Q4H342 | 7719 |
| O96738 | 7719 |
| Q4H2N7 | 7719 |
| Q5J2F2 | 9615 |
| P43698 | 9615 |
| Q5EVM6 | 34765 |
| Q5EVM7 | 34765 |
| B4KAU6 | 7230 |
| B4L4L5 | 7230 |
| B4KC72 | 7230 |
| Q6VT85 | 145470 |
| B4IV98 | 7245 |
| B4PQU8 | 7245 |
| B4PQU7 | 7245 |
| B4NA09 | 7260 |
| B4N261 | 7260 |
| B4NKI6 | 7260 |
| B4NKI5 | 7260 |
| O61286 | 51511 |
| Q95YL1 | 51511 |
| Q76EX9 | 51511 |
| B4GMA5 | 7234 |
| B4HAA5 | 7234 |
| B4GZB5 | 7234 |
| B3NJ53 | 7220 |
| B3P978 | 7220 |
| B3P004 | 7220 |
| B3P006 | 7220 |

| symbol Nanog | |
|---|---|
| B3MWX3 | 7217 |
| B3MYA4 | 7217 |
| B3LVC5 | 7217 |
| B3LVC4 | 7217 |
| B7U5S3 | 8090 |
| B4LVR0 | 7244 |
| B4M2J7 | 7244 |
| B4MBE1 | 7244 |
| B4MBE2 | 7244 |
| B6N2P9 | 7739 |
| B6L3E8 | 7739 |
| B6PK85 | 7739 |
| B4JHS5 | 7222 |
| B4JN60 | 7222 |
| Q7PL95 | 7227 |
| A8Y525 | 7227 |
| Q5BI77 | 7227 |
| A8Y527 | 7227 |
| P22808 | 7227 |
| Q0KHX8 | 7227 |
| Q00401 | 6162 |
| Q00400 | 6162 |
| Q8MUA8 | 63121 |
| A5HKM4 | 6359 |
| Q2WBW8 | 6359 |
| A5HKM5 | 6359 |
| A8KB41 | 7955 |
| B3DFT3 | 7955 |
| B3DG22 | 7955 |
| Q7YTC3 | 10224 |
| Q1PHP8 | 10224 |
| B2GUT3 | 8364 |
| Q28E82 | 8364 |
| Q642T9 | 8364 |
| Q28CW5 | 8364 |
| Q1WKL2 | 8364 |
| B1H161 | 8364 |
| B7ZN41 | 10090 |
| Q0QJG2 | 10090 |
| Q0QJG6 | 10090 |
| Q0QJG5 | 10096 |

| symbol HNF4a | |
|---|---|
| P49698 | 10090 |
| P22449 | 10116 |
| Q1I181 | 9823 |
| Q0ZHH5 | 9823 |
| P41235 | 9606 |
| A5JW41 | 9606 |
| B6ZGT3 | 9606 |
| Q5QPB7 | 9606 |

| symbol HNF4a | |
|---|---|
| Q9JJI9 | 64680 |
| Q7YRQ5 | 9913 |
| Q5ILH0 | 9031 |
| Q6PHH5 | 7955 |
| Q8AXB6 | 7955 |
| A8E5K2 | 7955 |
| Q91766 | 8355 |
| B6LWI0 | 7739 |
| Q4H3D4 | 7719 |
| Q4H3D5 | 7719 |
| O46174 | 7091 |
| O46175 | 7091 |
| B3MLY9 | 7217 |
| B4N7T1 | 7260 |
| Q29JT9 | 46245 |
| B4M9N0 | 7244 |
| B4NXH1 | 7245 |
| B3N7D7 | 7220 |
| A8DYX8 | 7227 |
| Q8IPF2 | 7227 |
| Q9VLI7 | 7227 |
| P49866 | 7227 |
| B4HYQ4 | 7238 |
| B4Q787 | 7240 |
| B4KEW7 | 7230 |
| B4GJ59 | 7234 |
| B4JQA2 | 7222 |
| B3S6R0 | 10228 |
| O77100 | 7159 |
| O77099 | 7159 |
| O77101 | 7159 |
| B0WSL6 | 7176 |
| B2RPP8 | 9606 |
| Q5ILG9 | 9031 |
| A7E311 | 9913 |
| B6ZGT4 | 9606 |
| Q059V1 | 10090 |
| Q08CC7 | 7955 |
| Q14541 | 9606 |
| Q3UP48 | 10090 |
| Q7YRQ4 | 9913 |
| Q7Z2V9 | 9606 |
| Q9WUU6 | 10090 |
| A2ICG7 | 10116 |
| B9VVT4 | 10116 |
| B9VVT7 | 10090 |

CLUSTERS

| symbol GATA6 | |
|---|---|
| Q92908 | 9606 |
| Q61169 | 10090 |
| P46153 | 10116 |
| P43693 | 9031 |
| Q7T1R5 | 8364 |
| Q6DFL7 | 8355 |
| Q641I8 | 8355 |
| Q91678 | 8355 |
| Q6NW63 | 7955 |
| Q9PTJ1 | 7955 |
| B4QXR5 | 7240 |
| A8NI30 | 240176 |
| B0D7V5 | 486041 |
| Q7YUE8 | 6289 |
| Q7YUE7 | 6289 |
| B8PAT0 | 561896 |
| Q09RL2 | 178876 |
| Q09RL3 | 40410 |
| Q5KA62 | 5207 |
| A3GF27 | 4924 |
| A3LNU1 | 4924 |
| P17429 | 162425 |
| Q9HEV3 | 162425 |
| A6XMX0 | 37727 |
| B6QH84 | 441960 |
| B6Q773 | 441960 |
| Q6CIN1 | 28985 |
| Q6CJ53 | 28985 |
| Q6CYD1 | 28985 |
| A5E7R1 | 379508 |
| A5E358 | 379508 |
| B3MXA8 | 7217 |
| P43692 | 9031 |
| P43694 | 9606 |
| P43695 | 8355 |
| P46152 | 10116 |
| P52168 | 7227 |
| Q08369 | 10090 |
| Q0Q0E4 | 9615 |
| Q16TJ4 | 7159 |
| Q294U2 | 46245 |
| Q3SZJ5 | 9913 |
| Q3UQR0 | 10090 |
| Q5M7L8 | 8364 |
| Q5U2V0 | 10116 |
| Q64HK6 | 7668 |
| Q8MIM5 | 9823 |
| Q95VY5 | 7165 |
| Q9BWX5 | 9606 |
| Q9W6U0 | 7955 |
| B4GML7 | 7234 |

| symbol GATA6 | |
|---|---|
| B4HLE9 | 7238 |
| B4JGA7 | 7222 |
| B4KAG0 | 7230 |
| B4LY20 | 7244 |
| B4NL23 | 7260 |
| B3DL22 | 8364 |
| P43696 | 8355 |
| P70005 | 8355 |
| Q1JQ00 | 7955 |
| Q28FV8 | 8364 |
| Q6GR12 | 8355 |
| Q6GR27 | 8355 |
| Q7T104 | 8355 |
| Q91677 | 8355 |
| A8DS45 | 8128 |
| B7ZKX0 | 9606 |
| B7ZKZ4 | 9606 |
| B9EHF7 | 10090 |
| Q16365 | 9606 |
| Q5IFM8 | 9606 |
| Q8VI87 | 10090 |
| A8WHL1 | 7227 |
| B3P3Y6 | 7220 |
| B4PR51 | 7245 |
| Q8T3J0 | 7227 |
| Q9VEZ8 | 7227 |
| B0WLM8 | 7176 |
| Q5IFI6 | 7159 |
| P97489 | 10090 |
| Q0VGI8 | 10090 |
| Q0VGJ0 | 10090 |

| symbol ESRRB | |
|---|---|
| Q61539 | 10090 |
| P11475 | 10116 |
| Q5F0P8 | 9606 |
| Q5F0P7 | 9606 |
| B6ZGU4 | 9606 |
| O95718 | 9606 |
| A5HNC2 | 8090 |
| A5HNC3 | 8090 |
| Q2I6X3 | 8078 |
| Q2I6X4 | 8078 |
| Q4RR47 | 99883 |
| Q16IU3 | 7159 |
| B0WYX7 | 7176 |
| B4H176 | 7234 |
| Q29FJ2 | 46245 |
| B4N4C7 | 7260 |
| B4J220 | 7222 |
| B4LCB9 | 7244 |

| symbol ESRRB | |
|---|---|
| Q8WS79 | 7227 |
| Q9VSE9 | 7227 |
| B4HJD7 | 7238 |
| B4PCU1 | 7245 |
| B3NF41 | 7220 |
| B3M9M5 | 7217 |
| B4QLJ3 | 7240 |
| A2RTQ7 | 10090 |
| A0JM86 | 8364 |
| A4IIT9 | 8364 |
| A5HNC1 | 8090 |
| A5HNC4 | 8090 |
| A8K6I2 | 9606 |
| B3KY84 | 9606 |
| B6LPX5 | 7739 |
| B6ZGU5 | 9606 |
| P62508 | 9606 |
| P62509 | 10090 |
| P62510 | 10116 |
| Q2I6X5 | 8078 |
| Q5QIB7 | 10116 |
| Q5RAM2 | 9601 |
| Q5UKY7 | 9031 |
| Q5XTQ9 | 7739 |
| Q6AX97 | 8355 |
| Q6Q6F4 | 7955 |
| Q6Q6F6 | 7955 |
| Q8CCV5 | 10090 |

| symbol SOX7 | |
|---|---|
| Q7T1L4 | 219752 |
| Q8JFA9 | 8090 |
| Q8JIP0 | 8090 |
| Q8UWL6 | 31033 |
| Q90Z22 | 43700 |
| Q90Z23 | 43700 |
| Q9BG91 | 9483 |
| Q9BG92 | 150249 |
| Q9DE39 | 8022 |
| Q9DFH1 | 7955 |
| Q9YGP7 | 8496 |
| B3DLD3 | 8364 |
| B7ZCS2 | 10090 |
| Q3UND6 | 10090 |
| A8WAC7 | 192075 |
| Q2PZ13 | 8030 |
| Q543C3 | 10090 |
| Q8CBZ8 | 10090 |
| Q8CCB9 | 10090 |
| Q91216 | 8022 |
| B4DKV0 | 9606 |

CLUSTERS

| symbol SOX7 | |
|---|---|
| Q9BT81 | 9606 |
| P40646 | 10090 |
| Q3U1W5 | 10090 |
| Q3U0W9 | 10090 |
| Q28GD5 | 8364 |
| O42342 | 8355 |
| B7ZSI0 | 8355 |
| B7ZSI1 | 8355 |
| Q6TEN5 | 7955 |
| Q6WNT2 | 31033 |
| Q4RR75 | 99883 |
| B5THP2 | 10224 |
| Q5IAS7 | 7757 |
| B6MX32 | 7739 |
| B6PIM1 | 7739 |
| Q3S378 | 45351 |
| A7RWK8 | 45351 |
| A4IIJ8 | 8364 |
| A5A763 | 9823 |
| B3M1J0 | 7217 |
| O18896 | 9823 |
| O55170 | 10116 |
| O73668 | 9031 |
| P35713 | 9606 |
| P43680 | 10090 |
| P48436 | 9606 |
| P56693 | 9606 |
| P57073 | 9606 |
| P57074 | 9031 |
| P61753 | 9544 |
| Q04886 | 10090 |
| Q04887 | 10090 |
| Q0VC26 | 9913 |
| Q4V7E4 | 10116 |
| Q52W08 | 7955 |
| Q6DFF5 | 8355 |
| Q6F2E7 | 8364 |
| Q6VVD7 | 8355 |
| Q7YRJ7 | 9615 |
| Q8AXX8 | 8355 |
| Q90XD1 | 7955 |
| Q90ZA9 | 9031 |
| Q90ZH7 | 8355 |
| Q90ZH8 | 8355 |
| Q9BG89 | 9598 |
| Q9DFH2 | 7955 |
| Q9VA17 | 7227 |
| Q9W757 | 9031 |
| B7ZR65 | 8355 |
| B7ZR67 | 8355 |
| B7ZR69 | 8355 |

| symbol SOX7 | |
|---|---|
| Q04888 | 10090 |
| Q0GA74 | 30331 |
| Q5BM60 | 31033 |
| Q6FHW7 | 9606 |
| Q6IZ48 | 99883 |
| Q6V4H7 | 69293 |
| Q6WNT1 | 31033 |
| Q8C916 | 10090 |
| Q90YL1 | 8355 |
| Q9IB79 | 8410 |
| B3P7T4 | 7220 |
| B4GN76 | 7234 |
| B4HZY6 | 7238 |
| B4K6I0 | 7230 |
| B4M5W5 | 7244 |
| B4NFA9 | 7260 |
| B4PN97 | 7245 |
| B4QSI9 | 7240 |
| Q29CD5 | 46245 |
| Q9VA16 | 7227 |
| A8WAC8 | 192075 |
| B0G0U8 | 47969 |
| B0LT38 | 9615 |
| B0LT39 | 9627 |
| B0LT40 | 494514 |
| B0LT41 | 192959 |
| B0M178 | 7764 |
| B1AVH1 | 10090 |
| B4DI77 | 9606 |
| B5ATG2 | 8319 |
| B5B424 | 34903 |
| B7X9J4 | 104659 |
| O57395 | 8022 |
| P48434 | 9031 |
| P61754 | 9600 |
| Q06FD9 | 8296 |
| Q0PV97 | 8081 |
| Q19R19 | 192075 |
| Q2MJB6 | 7757 |
| Q2NM26 | 93934 |
| Q2NM27 | 8839 |
| Q3HRN4 | 211491 |
| Q4F985 | 211488 |
| Q4KSL6 | 8090 |
| Q4LE25 | 8090 |
| Q53Y80 | 9606 |
| Q58FG5 | 7962 |
| Q5FYS4 | 211488 |
| Q6B7J1 | 215347 |
| Q6NXE1 | 7955 |
| Q6WNS8 | 31033 |

| symbol Sox17 | |
|---|---|
| Q61473 | 10090 |
| Q9H6I2 | 9606 |
| Q3KQ35 | 8355 |
| O42600 | 8355 |
| Q6PAE5 | 8355 |
| Q90ZH9 | 8355 |
| Q8AWH3 | 8364 |
| Q4LES8 | 9031 |
| B8R1E4 | 13489 |
| B8R1E5 | 13489 |
| Q6WNS5 | 31033 |
| Q8JGN3 | 43700 |
| A3FM17 | 57060 |
| A9J6L0 | 228608 |
| Q5PQZ5 | 7955 |
| Q9PTN0 | 7955 |
| Q5IAS7 | 7757 |
| B5THP2 | 10224 |
| B3NRB4 | 7220 |
| P40657 | 7227 |
| B4HR24 | 7238 |
| B4P6N5 | 7245 |
| B3MCY6 | 7217 |
| B4MPQ9 | 7260 |
| B4LLE3 | 7244 |
| B4KRC4 | 7230 |
| Q28XH5 | 46245 |
| B4GG51 | 7234 |
| B4J7M2 | 7222 |
| Q3S378 | 45351 |
| A7RWK8 | 45351 |
| Q4H2R8 | 7719 |

CLUSTERS

| symbol GATA3 | |
|---|---|
| A7RFY2 | 45351 |
| A8PRN4 | 425265 |
| A8Q0N5 | 6279 |
| A9ZND4 | 138676 |
| B0D7V5 | 486041 |
| B0X8S3 | 7176 |
| B2DBE1 | 10116 |
| B3DGG5 | 7955 |
| B3LZW4 | 7217 |
| B3NZW9 | 7220 |
| B3S690 | 10228 |
| B4G4Y3 | 7234 |
| B4HLP8 | 7238 |
| B4JF53 | 7222 |
| B4M159 | 7244 |
| B4NJ90 | 7260 |
| B4PSL4 | 7245 |
| B5X0Q3 | 8030 |
| B6LGL9 | 7739 |
| B7PFW5 | 6945 |
| B7WNQ9 | 9606 |
| B7Z0V4 | 7227 |
| O09100 | 10090 |
| O77156 | 7668 |
| P15976 | 9606 |
| P17678 | 9031 |
| P17679 | 10090 |
| P23769 | 9606 |
| P23770 | 8355 |
| P23771 | 9606 |
| P23772 | 10090 |
| P23773 | 8355 |
| P23824 | 9031 |
| P23825 | 9031 |
| P43429 | 10116 |
| P91623 | 7227 |
| Q08DV0 | 9913 |
| Q0ZHH4 | 9823 |
| Q172Z8 | 7159 |
| Q299A5 | 46245 |
| Q2TAR8 | 8355 |
| Q32NN9 | 8355 |
| Q3B845 | 10090 |
| Q3TZD6 | 10090 |
| Q3TZS0 | 10090 |
| Q3U0R5 | 10090 |
| Q3U223 | 10090 |
| Q3U320 | 10090 |
| Q3UIH9 | 10090 |
| Q4P855 | 5270 |
| Q53YE0 | 9606 |
| Q59IU1 | 8090 |
| Q5KST7 | 8090 |
| Q5VWG7 | 9606 |
| Q5VWG8 | 9606 |
| Q60I22 | 7764 |
| Q6DG89 | 7955 |
| Q6DIQ6 | 8364 |
| Q6S5H3 | 45351 |
| Q7T3G1 | 7955 |
| Q7TMX8 | 10090 |
| Q865U9 | 9823 |
| Q8QFW5 | 33514 |
| Q91428 | 7955 |
| Q924Y4 | 10116 |
| Q96BH0 | 9606 |
| Q96BH8 | 9606 |
| Q99NH5 | 10116 |
| Q9DBY9 | 10090 |
| Q9DC59 | 10090 |
| Q9P952 | 5334 |

| symbol Runx1 | |
|---|---|
| Q01196 | 9606 |
| Q03347 | 10090 |
| Q3UM65 | 10090 |
| Q63046 | 10116 |
| Q6PF39 | 8355 |
| Q9DGB8 | 7955 |
| Q6YI83 | 7739 |
| Q6YI85 | 7740 |
| B5LVY9 | 10224 |
| B5AXG5 | 45351 |
| B3RYC8 | 10228 |
| B5AXG6 | 6085 |
| Q0KHQ3 | 7227 |
| B4MSX7 | 7260 |
| B4MSY0 | 7260 |
| B4M3P0 | 7244 |
| B4M3N8 | 7244 |
| B4M225 | 7244 |
| B6S2Q2 | 7159 |
| Q177E8 | 7159 |
| B6S2Q5 | 7159 |
| Q173S1 | 7159 |
| B3NY30 | 7220 |
| B3NT81 | 7220 |
| B3NY28 | 7220 |
| B4H0R3 | 7234 |
| B4H0R7 | 7234 |
| B4GW80 | 7234 |
| B5DLS9 | 46245 |

| symbol Runx1 | |
|---|---|
| Q29GL5 | 46245 |
| B5DLK1 | 46245 |
| B3N110 | 7217 |
| B3N088 | 7217 |
| B3N111 | 7217 |
| B4L353 | 7230 |
| B4I6A4 | 7238 |
| B4I698 | 7238 |
| B4JXN6 | 7222 |
| B4JXN9 | 7222 |
| B4PYI7 | 7245 |
| B4PYI4 | 7245 |
| B4PWV3 | 7245 |
| Q8MPK9 | 6720 |
| Q8MPK8 | 6720 |
| A1XC55 | 283909 |
| Q6YI39 | 32264 |
| B4R3A1 | 7240 |
| B4R3A9 | 7240 |
| B7PIX2 | 6945 |
| A7UUZ5 | 7165 |
| B0W2H9 | 7176 |
| Q1WKT8 | 7233 |
| Q1WKT7 | 7243 |
| Q676B1 | 34765 |
| B2RMS4 | 9606 |
| Q90813 | 9031 |
| A0A9N7 | 31033 |
| A0A9P0 | 31033 |
| A1YIX8 | 8355 |
| A5HVZ3 | 31033 |
| B3DKD9 | 7955 |
| B5BNX8 | 8364 |
| Q13950 | 9606 |
| Q3L8U3 | 7955 |
| Q4VW83 | 7955 |
| Q4VW84 | 7955 |
| Q5T802 | 9606 |
| Q642Y3 | 31033 |
| Q6TYZ3 | 7955 |
| Q6TYZ4 | 7955 |
| Q6U1J2 | 7955 |
| Q6U1J3 | 7955 |
| Q8AY99 | 99883 |
| Q8JFD4 | 31033 |
| Q8UVG3 | 9031 |
| Q8UWC8 | 9031 |
| Q9DEF2 | 8090 |
| A0A9P3 | 31033 |

CLUSTERS

| symbol ID2 | |
|---|---|
| P41136 | 10090 |
| Q6QAE9 | 10036 |
| P41137 | 10116 |
| Q5RCH7 | 9601 |
| Q4R5J7 | 9541 |
| Q02363 | 9606 |
| Q53T66 | 9606 |
| Q3ZC46 | 9913 |
| Q2VIU1 | 9823 |
| O73933 | 9031 |
| Q3LSV1 | 93934 |
| Q6PBD7 | 8364 |
| B9EQ26 | 8030 |
| B9EM42 | 8030 |
| B9ELK6 | 8030 |
| Q9PWJ5 | 8355 |
| Q9YGL0 | 8355 |
| Q66J78 | 8355 |
| Q4VKK3 | 8022 |
| O42448 | 8022 |
| Q7SZQ2 | 7955 |
| Q9W619 | 8330 |
| Q4RM81 | 99883 |
| B6MX35 | 7739 |
| A8U072 | 7757 |
| B7QIB0 | 6945 |
| Q545T4 | 10090 |
| A4IF68 | 9913 |
| A7RVE5 | 45351 |
| B3MAE9 | 7217 |
| O42361 | 7955 |
| P18491 | 7227 |
| P41133 | 10090 |
| P41134 | 9606 |
| P41135 | 10116 |
| P41138 | 10116 |
| P41139 | 10090 |
| P47928 | 9606 |
| Q02535 | 9606 |
| Q06AV5 | 9823 |
| Q17HE4 | 7159 |
| Q29DX0 | 46245 |
| Q32PN8 | 7955 |
| Q5E981 | 9913 |
| Q5TX46 | 7165 |
| Q688C4 | 8364 |
| Q6GL62 | 8364 |
| Q6GTZ3 | 10090 |
| Q6PBJ0 | 7955 |
| Q712G9 | 9615 |
| Q7SZ28 | 8355 |

| symbol ID2 | |
|---|---|
| Q7ZXF3 | 8355 |
| Q8CH17 | 10116 |
| Q8QFX4 | 7955 |
| Q90X12 | 9031 |
| Q90X14 | 9031 |
| Q90X15 | 9031 |
| Q91399 | 8355 |
| A0JPJ2 | 10116 |
| A8K537 | 9606 |
| B3W6M6 | 9823 |
| B5X5B7 | 8030 |
| B5X7U5 | 8030 |
| B9ELA7 | 8030 |
| O42447 | 8022 |
| Q4VKK4 | 8022 |
| Q6IQW5 | 7955 |
| Q6VV65 | 8022 |
| B3NJB6 | 7220 |
| B4H496 | 7234 |
| B4HVJ7 | 7238 |
| B4PCN1 | 7245 |
| B4QLF2 | 7240 |
| Q7ZZB5 | 8355 |
| A8K1T8 | 9606 |
| B3W6M8 | 9823 |
| Q545W1 | 10090 |
| Q91417 | 8355 |
| Q9W620 | 8330 |
| Q9Z2W6 | 10116 |
| A2AHY3 | 10090 |
| P20067 | 10090 |
| Q544D2 | 10090 |
| B0WIA4 | 7176 |
| Q2F6A3 | 7091 |
| Q4V970 | 7955 |
| Q7T164 | 7955 |
| Q90X13 | 9031 |

| symbol CTBP1 | |
|---|---|
| O88712 | 10090 |
| Q56P31 | 9091 |
| Q9YHU0 | 8355 |
| Q5XHH5 | 8355 |
| Q13363 | 9606 |
| Q4KMQ8 | 9606 |
| Q7Z2Q5 | 9606 |
| Q66KL2 | 8364 |
| Q5ZIZ6 | 9031 |
| Q1RLQ4 | 7955 |
| Q17A41 | 7159 |

| symbol CTBP1 | |
|---|---|
| A8Q0T8 | 6279 |
| Q20595 | 6239 |
| Q9Z2F5 | 10116 |
| B3LW50 | 7217 |
| B3P196 | 7220 |
| B4G5K5 | 7234 |
| B4HGC4 | 7238 |
| B4JEW9 | 7222 |
| B4K8I6 | 7230 |
| B4M3U9 | 7244 |
| B4N9Y3 | 7260 |
| B4PPV3 | 7245 |
| B4R1X7 | 7240 |
| O46036 | 7227 |
| P56545 | 9606 |
| P56546 | 10090 |
| Q0VCQ1 | 9913 |
| Q28DQ9 | 8364 |
| Q299Q6 | 46245 |
| Q5ZMM8 | 9031 |
| Q640R6 | 8364 |
| Q7ZU55 | 7955 |
| Q9EQH5 | 10116 |
| Q9W758 | 8355 |
| A4V2S3 | 7227 |
| A9UL56 | 7955 |
| Q3UGL5 | 10090 |
| Q3UIX8 | 10090 |
| Q4RE44 | 99883 |
| Q56P32 | 9091 |
| Q5BU17 | 7955 |
| Q5BU18 | 7955 |
| Q5SQP8 | 9606 |
| Q6AZ26 | 10116 |
| Q6NZ25 | 7955 |
| Q7SZN5 | 7955 |
| Q7ZVY6 | 7955 |
| Q8IY44 | 9606 |
| Q91YZ2 | 10090 |
| Q9DEG6 | 7955 |
| Q9DEG7 | 7955 |

CLUSTERS

| symbol CGB | |
|---|---|
| P01233 | 9606 |
| B7FDQ4 | 9515 |
| B7FDQ3 | 9515 |
| Q14DX4 | 175797 |
| Q14DX3 | 175797 |
| Q9DG80 | 7998 |
| Q9DG92 | 8022 |
| P53543 | 13013 |
| P10256 | 8018 |
| Q5GJ78 | 62067 |
| P48253 | 8020 |
| P07732 | 74940 |
| A1ILL7 | 146921 |
| A6NKQ9 | 9606 |
| Q9GL37 | 9544 |
| Q6NT52 | 9606 |
| A1A5E0 | 9606 |
| B4XAN0 | 9598 |
| B4XAN2 | 9598 |
| B4XAN3 | 9598 |
| B4XAN4 | 9598 |
| B6VAC5 | 89462 |
| B7FDP5 | 9521 |
| B7FDP6 | 9521 |
| B7FDP7 | 9487 |
| B7FDP8 | 9487 |
| B7FDP9 | 9490 |
| B7FDQ0 | 9490 |
| B7FDQ1 | 9483 |
| B7FDQ2 | 9483 |
| O09108 | 10090 |
| O46482 | 9337 |
| O46641 | 9790 |
| O77805 | 9685 |
| O77835 | 9807 |
| P01229 | 9606 |
| P01230 | 10116 |
| P01231 | 9940 |
| P01232 | 9823 |
| P04651 | 9913 |
| P07434 | 9555 |
| P08751 | 9796 |
| P19794 | 9793 |
| P51500 | 9483 |
| Q19PY6 | 30521 |
| Q1EPQ9 | 10160 |
| Q1EPR3 | 79202 |
| Q1HG61 | 89462 |
| Q1RP71 | 9925 |
| Q2HWE4 | 10044 |
| Q2L4U0 | 10029 |

| symbol CGB | |
|---|---|
| Q2Q1P0 | 9600 |
| Q2Q1P1 | 9595 |
| Q2Q1P2 | 9598 |
| Q3HRV3 | 37293 |
| Q3MU78 | 9986 |
| Q3S2X5 | 39432 |
| Q3UUG9 | 10090 |
| Q400N5 | 10032 |
| Q62778 | 10116 |
| Q6EV78 | 9541 |
| Q6IY74 | 9986 |
| Q6UJC1 | 10047 |
| Q6V3B5 | 10036 |
| Q6V3B6 | 35658 |
| Q7JHA3 | 9986 |
| Q8HZR9 | 9649 |
| Q8WN18 | 9646 |
| Q924A8 | 10141 |
| Q95J85 | 13616 |
| Q9BDI9 | 74533 |
| Q9BEH1 | 9541 |
| Q9BEH2 | 9541 |

| symbol Cdh1 | |
|---|---|
| P09803 | 10090 |
| Q9R0T4 | 10116 |
| A6QLC4 | 9913 |
| Q6R8F2 | 9913 |
| P12830 | 9606 |
| A8K1U7 | 9606 |
| Q9UII7 | 9606 |
| B3GN61 | 9606 |
| Q9UII8 | 9606 |
| Q5RAX1 | 9601 |
| P08641 | 9031 |
| P33148 | 8355 |
| Q6NTM0 | 8355 |
| P33152 | 8355 |
| Q90Z37 | 7955 |
| Q4T7X6 | 99883 |
| Q4KML8 | 10090 |
| B2R6F4 | 9606 |
| B4DLF0 | 9606 |
| P10287 | 10090 |
| P22223 | 9606 |
| P30944 | 8355 |
| Q8BRE1 | 10090 |
| Q8BSL6 | 10090 |

| symbol TDGF1 | |
|---|---|
| P13385 | 9606 |
| Q8TCC1 | 9606 |
| B2R8C7 | 9606 |
| P51864 | 9606 |
| Q58D57 | 9913 |
| P51865 | 10090 |
| Q7TQ06 | 10090 |
| Q3UZP8 | 10090 |
| O57516 | 7955 |
| A9UMD1 | 8364 |
| B0JZQ5 | 8364 |
| Q0V9F3 | 8364 |
| Q91649 | 8355 |
| B7ZQM8 | 8355 |
| Q504I5 | 7955 |
| O57517 | 7955 |
| A7MCB2 | 7955 |

| symbol LIF | |
|---|---|
| P15018 | 9606 |
| O62728 | 452646 |
| Q27956 | 9913 |
| Q9GKZ8 | 9823 |
| Q256Y4 | 47230 |
| P17777 | 10116 |
| O88211 | 10116 |
| P09056 | 10090 |
| Q5SQP3 | 10090 |
| Q3U1H5 | 10090 |
| Q64015 | 10095 |
| B2RCW7 | 9606 |

| symbol NFKB1 | |
|---|---|
| P19838 | 9606 |
| B2RRQ6 | 10090 |
| P25799 | 10090 |
| Q66IP9 | 8355 |
| B5M235 | 10224 |
| B1NA65 | 6689 |
| Q6DF61 | 8364 |
| Q04861 | 9031 |
| Q0PHA8 | 9823 |
| Q1KNJ0 | 9913 |
| Q6F3J0 | 9615 |
| A3FJ60 | 119488 |
| A7XNS1 | 9823 |
| A7Z034 | 9913 |
| A8K5Y5 | 9606 |
| B3KVE8 | 9606 |
| O13075 | 9031 |

CLUSTERS

| symbol Prkaca (PKA) | |
|---|---|
| P05132 | 10090 |
| A1L1M0 | 10116 |
| P27791 | 10116 |
| P25321 | 10029 |
| P17612 | 9606 |
| Q9MZD9 | 9940 |
| P00517 | 9913 |
| P36887 | 9823 |
| Q8MJ44 | 9615 |
| Q95J97 | 9986 |
| Q6DE61 | 8355 |
| Q90WN3 | 8355 |
| Q28GZ8 | 8364 |
| A3KMS9 | 7955 |
| Q6DBV8 | 7955 |
| B6LG32 | 7739 |
| B6PNX0 | 7739 |
| Q16958 | 6500 |
| Q16957 | 6500 |
| Q4JIV3 | 6523 |
| Q4R0Q2 | 34608 |
| Q4R0Q0 | 34608 |
| Q4R0Q1 | 34608 |
| A5JNM1 | 7091 |
| B3N8G6 | 7220 |
| O97114 | 6943 |
| O97115 | 6943 |
| O97116 | 6943 |
| Q1HQW4 | 7159 |
| Q16933 | 29170 |
| Q4AC21 | 216498 |
| P12370 | 7227 |
| A4V0I0 | 7227 |
| B4Q7V0 | 7240 |
| B4JCE3 | 7222 |
| Q9NAS6 | 88217 |
| Q25115 | 7650 |
| P21137 | 6239 |
| B4KH13 | 7230 |
| Q7PXF7 | 7165 |
| Q55ZX5 | 5207 |
| Q5KP84 | 5207 |
| O42793 | 5466 |
| Q75T36 | 5462 |
| Q0PKT8 | 474922 |
| Q6BK94 | 4959 |
| Q6BWC6 | 4959 |
| A3FM70 | 117187 |
| A5DAZ1 | 4929 |
| A5DES7 | 4929 |
| Q9HEW0 | 5476 |

| symbol Prkaca (PKA) | |
|---|---|
| Q9P932 | 5476 |
| Q59Z23 | 5476 |
| Q5AP71 | 5476 |
| Q9Y777 | 5530 |
| Q6CS82 | 28985 |
| Q6CW38 | 28985 |
| A3LYD5 | 4924 |
| A3LPK9 | 4924 |
| Q6FSP1 | 5478 |
| Q6FJ83 | 5478 |
| A5DYL9 | 379508 |
| A5DT69 | 379508 |
| Q8J0I6 | 178876 |
| B3MUX5 | 7217 |
| B4NY66 | 7245 |
| B4HW89 | 7238 |
| B4M9H6 | 7244 |
| Q29NB1 | 46245 |
| B4MVA0 | 7260 |
| B4G7Y8 | 7234 |
| B0XFG0 | 7176 |
| A1DHC5 | 331117 |
| A2QCP3 | 425011 |
| A4R8U5 | 148305 |
| A6S414 | 332648 |
| A6SCB0 | 332648 |
| A7F7J7 | 325569 |
| A7SM62 | 45351 |
| A7TFJ0 | 436907 |
| A7TJ45 | 436907 |
| A8Q8C9 | 425265 |
| A9UQT5 | 81824 |
| B0D3V5 | 486041 |
| B0DXZ2 | 486041 |
| B2AUF3 | 5145 |
| B3M9V2 | 7217 |
| B3MT87 | 7217 |
| O13367 | 5270 |
| O43930 | 9606 |
| P05131 | 9913 |
| P05986 | 4932 |
| P06244 | 4932 |
| P06245 | 4932 |
| P16911 | 7227 |
| P16912 | 7227 |
| P22612 | 9606 |
| P22694 | 9606 |
| P40376 | 4896 |
| P51817 | 9606 |
| P68181 | 10090 |
| P68182 | 10116 |

| symbol Prkaca (PKA) | |
|---|---|
| Q16NW2 | 7159 |
| Q29CB1 | 46245 |
| Q2UEY6 | 5062 |
| Q3ZB92 | 7955 |
| Q4X0V1 | 5085 |
| Q55MZ9 | 5207 |
| Q5BK52 | 10116 |
| Q5KBC8 | 5207 |
| Q6CCL9 | 4952 |
| Q755B5 | 33169 |
| Q758E1 | 33169 |
| Q7JP68 | 6239 |
| Q7T374 | 7955 |
| Q7ZWV0 | 8355 |
| Q8SRK8 | 6035 |
| Q922R0 | 10090 |
| Q99079 | 5270 |
| Q9P466 | 5141 |
| Q9P472 | 162425 |
| A1C6Q4 | 5057 |
| A6ZQG8 | 307796 |
| A6ZW51 | 307796 |
| A6ZZF8 | 307796 |
| B0LW64 | 5599 |
| B0XSP3 | 451804 |
| B2WHZ4 | 426418 |
| B3LKM6 | 285006 |
| B3LPV9 | 285006 |
| B3LQT8 | 285006 |
| B5VL21 | 545124 |
| B5VSX7 | 545124 |
| B6HHD2 | 500485 |
| B6QK74 | 441960 |
| B8MLH4 | 441959 |
| B8NH99 | 332952 |
| P87077 | 5061 |
| Q01143 | 148305 |
| Q0CYS7 | 341663 |
| Q0U5G3 | 13684 |
| Q1DWD0 | 5501 |
| Q1KTF1 | 54734 |
| Q8J129 | 5085 |
| Q96UM3 | 5085 |
| Q9HGU6 | 34373 |
| Q9UUS9 | 62688 |
| A6QNX4 | 9913 |
| A8K8B9 | 9606 |
| B2RB89 | 9606 |
| B4DKB0 | 9606 |
| B4E2L0 | 9606 |
| B7ZA00 | 9606 |

CLUSTERS

| symbol Prkaca (PKA) | |
|---|---|
| P05383 | 9823 |
| P68180 | 10029 |
| Q5R472 | 9601 |
| Q5R9Y7 | 9601 |
| Q5RC73 | 9601 |
| Q800K1 | 8022 |
| A6R2N1 | 339724 |
| Q5DF14 | 6182 |
| A8N588 | 240176 |
| Q12741 | 4808 |
| Q8NIL1 | 40410 |
| Q9HFW0 | 40410 |
| A1DEX4 | 331117 |
| A7ECY2 | 325569 |
| B0Y409 | 451804 |
| Q1DSD8 | 5501 |
| Q2H4R5 | 38033 |
| Q2KFU8 | 242507 |
| Q5B413 | 162425 |
| Q6QE15 | 5180 |
| Q70KQ0 | 5085 |
| Q7SHX1 | 5141 |
| Q86ZN6 | 148305 |
| B3NDN9 | 7220 |
| B4ITX2 | 7245 |
| B4PD13 | 7245 |
| B4QLR0 | 7240 |
| Q194S5 | 7227 |
| B1AVU0 | 10090 |
| B1AVU1 | 10090 |
| Q3UDT1 | 10090 |
| Q4RY94 | 99883 |
| B4HIK7 | 7238 |
| B6K6I0 | 402676 |
| B1APG3 | 9606 |
| Q5DDU1 | 6182 |
| B4GNC3 | 7234 |
| B1APG4 | 9606 |
| Q8NIK9 | 40410 |
| P49673 | 6253 |
| Q20541 | 6239 |
| Q8MQ39 | 6239 |

| symbol TCF3 | |
|---|---|
| P15923 | 9606 |
| P15806 | 10090 |
| P98180 | 10036 |
| P21677 | 10116 |
| Q68G29 | 10116 |
| Q70IK3 | 9031 |
| Q5ZL14 | 9031 |
| Q70IK2 | 9031 |
| Q01978 | 8355 |
| Q7ZXY5 | 8355 |
| A4IIL6 | 8364 |
| Q56R80 | 7998 |
| Q32T22 | 7998 |
| B1H1L4 | 7955 |
| B3MJG5 | 7217 |
| B0WJ83 | 7176 |
| Q17588 | 6239 |
| Q17358 | 6239 |
| Q17326 | 6239 |
| A0JN84 | 9913 |
| P11420 | 7227 |
| P15881 | 9615 |
| P15884 | 9606 |
| P51514 | 10116 |
| Q16TH4 | 7159 |
| Q3ZBP9 | 9913 |
| Q4H3N7 | 7719 |
| Q60722 | 10090 |
| Q61286 | 10090 |
| Q62655 | 10116 |
| Q6NSM7 | 7955 |
| Q99081 | 9606 |
| A6QPK5 | 9913 |
| B3KT62 | 9606 |
| B3KUC0 | 9606 |
| B3KVA4 | 9606 |
| B4DZH5 | 9606 |
| B7Z5M6 | 9606 |
| B7Z6Y1 | 9606 |
| Q0V9M4 | 8364 |
| Q3UZE5 | 10090 |
| Q641A7 | 8355 |
| Q86TC1 | 9606 |
| Q8CE98 | 10090 |
| B3N9N6 | 7220 |
| B4HWN1 | 7238 |
| B4JP31 | 7222 |
| B4MG51 | 7244 |
| B4NGK5 | 7260 |
| B4NZN2 | 7245 |
| B7FNP0 | 7227 |

| symbol TCF3 | |
|---|---|
| Q29CW9 | 46245 |
| Q86PA6 | 7227 |
| Q2TB39 | 9606 |
| Q6QR57 | 7998 |
| Q6QR58 | 7998 |
| Q86VM2 | 9606 |
| Q16TH3 | 7159 |
| B4DT37 | 9606 |
| B4DUG3 | 9606 |
| B4E1W1 | 9606 |
| Q8K1X3 | 10090 |
| Q91XK1 | 10090 |
| Q91YV0 | 10090 |

| symbol Yap1 | |
|---|---|
| P46938 | 10090 |
| Q2EJA0 | 10116 |
| Q7Z574 | 9606 |
| Q8IUY9 | 9606 |
| A1L1U5 | 7955 |
| Q1L8J7 | 7955 |
| P46936 | 9031 |
| Q32NJ6 | 8355 |
| B6NNB9 | 7739 |
| B2CMB6 | 7091 |
| A7SLN5 | 45351 |
| B4PAT4 | 7245 |
| Q45VV3 | 7227 |
| Q0E8X1 | 7227 |
| Q95TU5 | 7227 |
| A2RVH0 | 7227 |
| A2RVH4 | 7227 |
| Q7KVG6 | 7227 |
| B3NQ71 | 7220 |
| B4I2C6 | 7238 |
| Q5TR76 | 7165 |
| B7PRF1 | 6945 |
| P46937 | 9606 |

CLUSTERS

| symbol PRKCA (aPKC) | |
|---|---|
| A0JM65 | 8364 |
| A0JNZ5 | 10090 |
| A1CBE3 | 5057 |
| A1DDZ6 | 331117 |
| A2RA98 | 425011 |
| A3GHJ5 | 4924 |
| A4QQ12 | 148305 |
| A5D7N0 | 9913 |
| A6QZ29 | 339724 |
| A6S4G3 | 332648 |
| A6ZKJ9 | 307796 |
| A7F8U5 | 325569 |
| A7TMA2 | 436907 |
| A8DYG9 | 7227 |
| A8KBH6 | 8364 |
| A8PPV9 | 6279 |
| A8Q595 | 425265 |
| A9L903 | 9555 |
| A9V750 | 81824 |
| B0CVH0 | 486041 |
| B0JYP1 | 9913 |
| B0Y142 | 451804 |
| B1MTW7 | 9523 |
| B2AMZ1 | 5145 |
| B2KIL5 | 59479 |
| B2R5T1 | 9606 |
| B2VTM3 | 426418 |
| B3LNN5 | 285006 |
| B3MD92 | 7217 |
| B3MD93 | 7217 |
| B3NP50 | 7220 |
| B3NP51 | 7220 |
| B3RFF0 | 42254 |
| B3RQ26 | 10228 |
| B4GBQ2 | 7234 |
| B4GBQ5 | 7234 |
| B4HTB2 | 7238 |
| B4HTB4 | 7238 |
| B4JVL2 | 7222 |
| B4JVL3 | 7222 |
| B4KSR0 | 7230 |
| B4KSR1 | 7230 |
| B4LP68 | 7244 |
| B4LP69 | 7244 |
| B4MR94 | 7260 |
| B4MR97 | 7260 |
| B4P5T9 | 7245 |
| B4P8K3 | 7245 |
| B4QIH6 | 7240 |
| B4QIH7 | 7240 |
| B5BU22 | 9606 |

| symbol PRKCA (aPKC) | |
|---|---|
| B5DFC4 | 10116 |
| B5FWA6 | 30611 |
| B5VDP0 | 545124 |
| B6HK33 | 500485 |
| B6JW41 | 402676 |
| B6Q4R3 | 441960 |
| B7NZS1 | 9986 |
| B7PWS6 | 6945 |
| B7SJR4 | 7141 |
| B7Z3V9 | 9606 |
| B7Z3W6 | 9606 |
| B8LXH5 | 441959 |
| B8NKP5 | 332952 |
| O01715 | 6087 |
| O42632 | 5016 |
| O61224 | 56443 |
| O76850 | 7373 |
| P04409 | 9913 |
| P05126 | 9913 |
| P05129 | 9606 |
| P05130 | 7227 |
| P05696 | 10116 |
| P05771 | 9606 |
| P05772 | 9986 |
| P10102 | 9986 |
| P10829 | 9986 |
| P13677 | 7227 |
| P17252 | 9606 |
| P20444 | 10090 |
| P24583 | 4932 |
| P36583 | 4896 |
| P43057 | 5476 |
| P63318 | 10090 |
| P63319 | 10116 |
| P68403 | 10116 |
| P68404 | 10090 |
| P87253 | 5141 |
| P90980 | 6239 |
| Q00078 | 5061 |
| Q0CW56 | 341663 |
| Q0UME1 | 13684 |
| Q16974 | 6500 |
| Q17M58 | 7159 |
| Q1DKF7 | 5501 |
| Q25378 | 7653 |
| Q290K1 | 46245 |
| Q290K2 | 46245 |
| Q2H265 | 38033 |
| Q2PJA7 | 6239 |
| Q2TSD3 | 9606 |
| Q2U6A7 | 5062 |

| symbol PRKCA (aPKC) | |
|---|---|
| Q3UN66 | 10090 |
| Q4AED6 | 7091 |
| Q4R4U2 | 9541 |
| Q4VA93 | 10090 |
| Q4WVG0 | 5085 |
| Q55VU6 | 5207 |
| Q5ANK2 | 5476 |
| Q5BH74 | 162425 |
| Q5F3X1 | 9031 |
| Q5KKE3 | 5207 |
| Q6AZF7 | 8355 |
| Q6BI27 | 4959 |
| Q6C292 | 4952 |
| Q6FJ43 | 5478 |
| Q6UB96 | 40410 |
| Q6UB97 | 178876 |
| Q75BT0 | 33169 |
| Q76G54 | 162425 |
| Q7LZQ8 | 8355 |
| Q7LZQ9 | 8355 |
| Q7SY24 | 7955 |
| Q7T2C5 | 7955 |
| Q873Y9 | 5022 |
| Q8J213 | 28985 |
| Q8JFZ9 | 31033 |
| Q99014 | 51453 |
| Q9HF10 | 34373 |
| Q9HGK8 | 42251 |
| Q9UVJ5 | 40559 |
| Q9Y792 | 29908 |
| Q9Y7C1 | 148305 |

| symbol LIFR | |
|---|---|
| P42702 | 9606 |
| Q5XNR9 | 9615 |
| P42703 | 10090 |
| Q3U234 | 10090 |
| O70535 | 10116 |
| Q8QFQ7 | 9031 |
| A2CE47 | 7955 |
| Q58EK6 | 7955 |
| A8WH83 | 7955 |
| Q6UAM7 | 99883 |
| Q4RPS1 | 99883 |
| A8K1Z4 | 9606 |

CLUSTERS

| symbol TLE2 | |
|---|---|
| Q04725 | 9606 |
| Q9WVB2 | 10090 |
| Q3UN01 | 10090 |
| Q496Z7 | 10116 |
| B3RR30 | 10228 |
| O13166 | 7955 |
| P16371 | 7227 |
| Q296Z2 | 46245 |
| B1H1Y7 | 8355 |
| B3DGV3 | 7955 |
| B3KUA2 | 9606 |
| B4DPT0 | 9606 |
| B5THN7 | 10224 |
| O42469 | 8355 |
| Q04726 | 9606 |
| Q08122 | 10090 |
| Q1LXH1 | 7955 |
| Q3LFR4 | 31033 |
| Q3TY99 | 10090 |
| Q5FV99 | 8364 |
| Q6DEF0 | 8355 |
| Q6PHP0 | 10090 |
| Q6PI57 | 9606 |
| Q6PRX2 | 9606 |
| Q6PRX3 | 9606 |
| Q98TH2 | 7955 |
| Q9DGK0 | 8355 |
| Q9JIT3 | 10116 |
| B0WQA4 | 7176 |
| B3LY32 | 7217 |
| B3P6A6 | 7220 |
| B4GEG0 | 7234 |
| B4ICI5 | 7238 |
| B4JGJ3 | 7222 |
| B4LYS6 | 7244 |
| B4NK36 | 7260 |
| B4PSX2 | 7245 |
| B4QW19 | 7240 |
| Q17BC6 | 7159 |
| Q5UBX7 | 7654 |
| Q7PMQ2 | 7165 |

| symbol ACTR3 (ARP3) | |
|---|---|
| P61158 | 9606 |
| Q5R8R1 | 9601 |
| P61157 | 9913 |
| Q4V7C7 | 10116 |
| Q99JY9 | 10090 |
| Q3ULF7 | 10090 |
| B5APU4 | 9823 |
| Q90WD0 | 9031 |
| Q4RK67 | 99883 |
| Q6P309 | 8364 |
| Q6PUQ3 | 7955 |
| Q8JGM2 | 7955 |
| Q801P7 | 8355 |
| O73723 | 31033 |
| B6PL52 | 7739 |
| A3QX13 | 55567 |
| A7S3B3 | 45351 |
| Q9U5H4 | 7650 |
| B4LGZ8 | 7244 |
| Q7PQ13 | 7165 |
| Q16MG7 | 7159 |
| B0XB57 | 7176 |
| B0X7Y3 | 7176 |
| B3M425 | 7217 |
| B4KWX4 | 7230 |
| B4PCS6 | 7245 |
| B3NF57 | 7220 |
| P32392 | 7227 |
| B4HIZ2 | 7238 |
| Q29FE3 | 46245 |
| B4H1G3 | 7234 |
| B4IWR1 | 7222 |
| B4MXD7 | 7260 |
| B3RR76 | 10228 |
| A9V006 | 81824 |
| Q9N4I0 | 6239 |
| Q61WW9 | 6238 |
| A8P700 | 6279 |
| A8NTZ1 | 240176 |
| B0DA04 | 486041 |
| Q6C3K0 | 4952 |
| Q2UEC7 | 5062 |
| B8NH60 | 332952 |
| B0Y102 | 451804 |
| A1DE34 | 331117 |
| A2RAH8 | 425011 |
| Q4WVC0 | 5085 |
| A3LYF0 | 4924 |
| Q0CW19 | 341663 |
| A1CBA8 | 5057 |
| A6S1U1 | 332648 |

| symbol ACTR3 (ARP3) | |
|---|---|
| B6HIY6 | 500485 |
| P78712 | 5141 |
| B6Q5H3 | 441960 |
| A8QCN6 | 425265 |
| Q6FUU8 | 5478 |
| Q2GPB8 | 38033 |
| Q5BH40 | 162425 |
| Q4PFS9 | 5270 |
| B2B0V9 | 5145 |
| A4R4C8 | 148305 |
| B4NT15 | 7240 |
| Q53QM2 | 9606 |
| Q59Z11 | 5476 |
| A5DAY0 | 4929 |
| B8LTI8 | 441959 |

| symbol PARD3 | |
|---|---|
| A2BEM5 | 7955 |
| A2BEM6 | 7955 |
| A2BEM7 | 7955 |
| A5D6P2 | 10090 |
| A7RRU6 | 45351 |
| B2RUK1 | 10090 |
| B3MW54 | 7217 |
| B3NWC4 | 7220 |
| B4GY90 | 7234 |
| B4JKF9 | 7222 |
| B4NC67 | 7260 |
| B4Q264 | 7245 |
| B7ZNY3 | 10090 |
| O96782 | 7227 |
| Q0KHR3 | 7227 |
| Q0PVE6 | 9031 |
| Q17353 | 6239 |
| Q29HU6 | 46245 |
| Q5VWV0 | 9606 |
| Q5VWV1 | 9606 |
| Q5VWV2 | 9606 |
| Q5VWV4 | 9606 |
| Q5VWV5 | 9606 |
| Q6GN19 | 8355 |
| Q6IQ47 | 9606 |
| Q6QUY1 | 7955 |
| Q6QUY2 | 7955 |
| Q7T1R3 | 7955 |
| Q8TEW0 | 9606 |
| Q99NH2 | 10090 |
| Q9VX75 | 7227 |
| Q9Z340 | 10116 |

CLUSTERS

| symbol Gata4 | |
|---|---|
| Q08369 | 10090 |
| B9EHF7 | 10090 |
| Q8VI87 | 10090 |
| Q3UYJ1 | 10090 |
| P46152 | 10116 |
| P43694 | 9606 |
| B7ZKX0 | 9606 |
| Q5IFM8 | 9606 |
| B7ZKZ4 | 9606 |
| Q0Q0E4 | 9615 |
| Q8MIM5 | 9823 |
| B3DL22 | 8364 |
| Q28FV8 | 8364 |
| Q6GR27 | 8355 |
| Q91677 | 8355 |
| Q6GR12 | 8355 |
| A8DS45 | 8128 |
| Q09JY7 | 7955 |
| B8JKU1 | 7955 |
| Q9PTJ2 | 7955 |
| Q4RR24 | 99883 |
| B0LD06 | 283909 |
| B3MXA8 | 7217 |
| Q9NJ18 | 7213 |
| Q294U2 | 46245 |
| B4GML7 | 7234 |
| B4HLE9 | 7238 |
| B4PR51 | 7245 |
| B4NL23 | 7260 |
| B0WLM8 | 7176 |
| Q16TJ4 | 7159 |
| Q5IFI6 | 7159 |
| P52167 | 7091 |
| B6K6C1 | 402676 |
| Q95VY5 | 7165 |
| P52168 | 7227 |
| A8WHL1 | 7227 |
| Q9VEZ8 | 7227 |
| Q8T3J0 | 7227 |
| Q4H3F6 | 7719 |
| P43692 | 9031 |
| P43693 | 9031 |
| P43695 | 8355 |
| P46153 | 10116 |
| Q3SZJ5 | 9913 |
| Q3UQR0 | 10090 |
| Q5M7L8 | 8364 |
| Q5U2V0 | 10116 |
| Q61169 | 10090 |
| Q641I8 | 8355 |
| Q64HK6 | 7668 |

| symbol Gata4 | |
|---|---|
| Q7T1R5 | 8364 |
| Q92908 | 9606 |
| Q9BWX5 | 9606 |
| Q9PTJ1 | 7955 |
| Q9W6U0 | 7955 |
| B4JGA7 | 7222 |
| B4KAG0 | 7230 |
| B4LY20 | 7244 |
| P43696 | 8355 |
| P70005 | 8355 |
| Q1JQ00 | 7955 |
| Q6DFL7 | 8355 |
| Q6NW63 | 7955 |
| Q7T104 | 8355 |
| Q91678 | 8355 |
| Q16365 | 9606 |
| B3P3Y6 | 7220 |
| P97489 | 10090 |
| Q0VGI8 | 10090 |
| Q0VGJ0 | 10090 |

| symbol Jak2 | |
|---|---|
| Q62120 | 10090 |
| Q62689 | 10116 |
| O19064 | 9823 |
| O60674 | 9606 |
| Q506Q0 | 9606 |
| A8K910 | 9606 |
| Q8IXP2 | 9606 |
| Q5RB23 | 9601 |
| Q75R65 | 9031 |
| A0JM01 | 8364 |
| Q9PVI2 | 47145 |
| O93596 | 7955 |
| Q4FZX4 | 8355 |
| Q9PTN6 | 7962 |
| B6N510 | 7739 |
| Q4H3A3 | 7719 |
| B4L4A0 | 7230 |
| B4MCY4 | 7244 |
| Q29H22 | 46245 |
| B4NE99 | 7260 |
| B4NC66 | 7260 |
| B3N089 | 7217 |
| Q24592 | 7227 |
| B4DYV1 | 9606 |
| Q9TTI9 | 9823 |
| Q9TTJ0 | 9823 |
| B3NUE4 | 7220 |
| B4PYC0 | 7245 |

| symbol LATS2 | |
|---|---|
| Q9NRM7 | 9606 |
| Q7TSJ6 | 10090 |
| B2BJC0 | 7955 |
| B2BJC1 | 7955 |
| B0S8I4 | 7955 |
| A4III9 | 8364 |
| Q4RTQ1 | 99883 |
| B4HZV7 | 7238 |
| B3MT80 | 7217 |
| B6NTD6 | 7739 |
| O95835 | 9606 |
| Q29CB7 | 46245 |
| Q56SE2 | 9615 |
| Q58FB8 | 7955 |
| Q641H7 | 8355 |
| Q8BYR2 | 10090 |
| Q9VA38 | 7227 |
| B3P7W4 | 7220 |
| B4PML7 | 7245 |
| Q24096 | 7227 |
| Q24590 | 7227 |
| B2RY46 | 10090 |
| Q5RIL6 | 7955 |
| B4GNB6 | 7234 |

| symbol PECAM1 | |
|---|---|
| A7LKP4 | 9796 |
| A8K3S7 | 9606 |
| B0CLW7 | 7955 |
| B1ARB1 | 10090 |
| B1ARB2 | 10090 |
| B1ARB3 | 10090 |
| B1MV75 | 9796 |
| B5X2G9 | 8030 |
| B7Z8Y6 | 9606 |
| P16284 | 9606 |
| P51866 | 9913 |
| P79390 | 9909 |
| Q08481 | 10090 |
| Q0VCP8 | 9913 |
| Q2TAE7 | 8355 |
| Q3SWT0 | 10116 |
| Q3TES6 | 10090 |
| Q4RLG1 | 99883 |
| Q5W5X8 | 8355 |
| Q8CAW4 | 10090 |
| Q922E0 | 10090 |
| Q95242 | 9823 |

CLUSTERS

| symbol FOXD3 | |
|---|---|
| A2AD43 | 10090 |
| A2BDY3 | 10090 |
| A2CE80 | 7955 |
| A8Q3U6 | 6279 |
| B0UXI2 | 7955 |
| B2RRJ0 | 10090 |
| B3KVK3 | 9606 |
| B3MEK5 | 7217 |
| B3NNX7 | 7220 |
| B4H512 | 7234 |
| B4I8C7 | 7238 |
| B4J764 | 7222 |
| B4KSL2 | 7230 |
| B4LMW5 | 7244 |
| B4MJL9 | 7260 |
| B4P8L4 | 7245 |
| B4QI21 | 7240 |
| B7P2M9 | 6945 |
| B9EGF3 | 9606 |
| B9EGL7 | 9606 |
| O35392 | 10090 |
| O60548 | 9606 |
| O73782 | 7955 |
| P79770 | 9031 |
| P79772 | 9031 |
| P91587 | 7719 |
| Q02361 | 7227 |
| Q0PV86 | 7729 |
| Q12950 | 9606 |
| Q16676 | 9606 |
| Q16GR9 | 7159 |
| Q18694 | 6239 |
| Q28X48 | 46245 |
| Q3SYB3 | 9606 |
| Q3UQW8 | 10090 |
| Q4H3J2 | 7719 |
| Q4RJ48 | 99883 |
| Q4RUK2 | 99883 |
| Q4S2D4 | 99883 |
| Q502Q4 | 7955 |
| Q5JTX9 | 9606 |
| Q5M7L9 | 8364 |
| Q5VV16 | 9606 |
| Q60688 | 10090 |
| Q61060 | 10090 |
| Q61345 | 10090 |
| Q6DHP5 | 7955 |
| Q6F2E4 | 8364 |
| Q6NXD6 | 7955 |
| Q6VAE7 | 9598 |
| Q6VAE8 | 9598 |

| symbol FOXD3 | |
|---|---|
| Q6VAE9 | 9598 |
| Q6VAF0 | 9593 |
| Q6VAF2 | 9600 |
| Q6VAF3 | 9600 |
| Q6VB84 | 9606 |
| Q6VB85 | 9606 |
| Q6VB86 | 9606 |
| Q7Q419 | 7165 |
| Q8MUS6 | 7739 |
| Q8WXT5 | 9606 |
| Q90WN4 | 8355 |
| Q95YK7 | 51511 |
| Q98937 | 9031 |
| Q9DEN3 | 8355 |
| Q9DEN4 | 8355 |
| Q9NU39 | 9606 |
| Q9PSY4 | 8355 |
| Q9UJU5 | 9606 |

| symbol Klf5 | |
|---|---|
| Q9Z0Z7 | 10090 |
| B4DL96 | 9606 |
| Q13887 | 9606 |
| Q5T6X2 | 9606 |
| B4E2I1 | 9606 |
| A1XWJ7 | 9823 |
| A4IFB2 | 9913 |
| Q66HP1 | 10116 |
| Q9N1Y4 | 9986 |
| Q4KLU4 | 8355 |
| Q6DIM6 | 8364 |
| B8JL64 | 7955 |
| B6KXG3 | 7739 |
| B6NSW5 | 7739 |
| B7P607 | 6945 |
| Q2PHB0 | 218308 |
| B4LDI2 | 7244 |
| B4IZV3 | 7222 |
| B3NBY1 | 7220 |
| Q9VZN4 | 7227 |
| B4HTT2 | 7238 |
| B4PH49 | 7245 |
| B3M818 | 7217 |
| Q4H3A0 | 7719 |
| B4N578 | 7260 |
| B4KYB6 | 7230 |
| A7S781 | 45351 |
| B5DPM3 | 46245 |
| A2TJX0 | 9606 |
| Q923C0 | 10090 |

| symbol MYC | |
|---|---|
| P01106 | 9606 |
| P23583 | 9598 |
| A1YG22 | 9597 |
| B8XIA5 | 9544 |
| P49033 | 9580 |
| A2T7L5 | 9600 |
| Q9MZT9 | 9395 |
| P49032 | 9483 |
| Q9MZU0 | 482537 |
| Q28566 | 9940 |
| Q9MZT7 | 9438 |
| Q28350 | 9615 |
| Q2HJ27 | 9913 |
| Q29031 | 9823 |
| Q9MZT8 | 9405 |
| B2RSN1 | 10090 |
| P01108 | 10090 |
| P09416 | 10116 |
| P68271 | 9685 |
| Q9MZT6 | 9988 |
| P22555 | 9995 |
| P01109 | 9031 |
| Q6P1T1 | 8364 |
| Q6NRN4 | 8355 |
| P15171 | 8355 |
| P06171 | 8355 |
| Q2TAU1 | 8355 |
| Q90342 | 7962 |
| Q90341 | 7962 |
| Q7ZVS9 | 7955 |
| B0UY64 | 7955 |
| P52160 | 7955 |
| Q2L4Q4 | 8022 |
| Q91421 | 8022 |
| P49709 | 7957 |
| Q9DGD0 | 7957 |
| Q3LFR6 | 31033 |
| Q9DFB9 | 7998 |
| Q589Y4 | 7741 |
| B6N4V3 | 7739 |
| B5THP1 | 10224 |
| A7RIE4 | 45351 |
| A8WFE7 | 9606 |
| B4E1N7 | 9606 |

CLUSTERS

| symbol GJA3 | |
|---|---|
| Q9Y6H8 | 9606 |
| B2RRM1 | 10090 |
| Q64448 | 10090 |
| P29414 | 10116 |
| Q9TU17 | 9940 |
| Q8JFD2 | 7955 |
| P41987 | 9913 |
| B0BM18 | 8364 |
| P29415 | 9031 |
| Q7ZYF9 | 8355 |
| Q532L5 | 8355 |
| Q4S8R0 | 99883 |
| P51915 | 29154 |
| B5X655 | 8030 |
| B5XFL7 | 8030 |
| B5X5N6 | 8030 |
| Q1HG93 | 61084 |
| Q9DGB5 | 143350 |
| Q6GLA5 | 8364 |
| Q8AWR4 | 7955 |
| B0R0X0 | 7955 |
| A7YYA8 | 7955 |
| B3DHP8 | 7955 |
| P51916 | 29154 |
| Q5TYP1 | 7955 |
| Q7T047 | 7955 |

| symbol Klf4 | |
|---|---|
| Q60793 | 10090 |
| Q923V7 | 10116 |
| B4YSE4 | 9823 |
| Q52JJ4 | 9823 |
| A7YWE2 | 9913 |
| Q4R355 | 9541 |
| B8XIA4 | 9544 |
| O43474 | 9606 |
| Q28D29 | 8364 |
| A9X6Q5 | 7955 |
| B5X2J1 | 8030 |
| B5X3X7 | 8030 |
| B5X170 | 8030 |
| Q4H2J1 | 7719 |
| B7P1E1 | 6945 |
| B6MI74 | 7739 |
| B2RS60 | 10090 |
| Q13351 | 9606 |
| Q28BQ7 | 8364 |
| Q28D19 | 8364 |
| Q8QGW5 | 8355 |
| Q90XE7 | 7955 |
| Q90XS5 | 8355 |

| symbol Klf4 | |
|---|---|
| Q9DFS2 | 7955 |
| Q9ET58 | 10116 |
| Q9Y5W3 | 9606 |
| B4X5Z2 | 9823 |
| Q3V293 | 10090 |
| Q60843 | 10090 |
| B4YSE2 | 9823 |
| P46099 | 10090 |
| Q0P5B6 | 9913 |
| Q1RME5 | 10090 |
| Q3TZ82 | 10090 |
| Q2TAV3 | 8355 |
| Q7SZU5 | 8355 |
| Q8QGW6 | 8355 |
| Q1LXI7 | 7955 |
| Q4RWQ1 | 99883 |
| Q6P3M5 | 7955 |
| Q90XE8 | 7955 |

| symbol DPP3 | |
|---|---|
| Q6CE11 | 4952 |
| A1CGS6 | 5057 |
| Q4WNL2 | 5085 |
| B0Y5F9 | 451804 |
| A7E778 | 325569 |
| B6HRY6 | 500485 |
| A1CY04 | 331117 |
| Q2UG48 | 5062 |
| B0S4Q0 | 245881 |
| A2QHM6 | 425011 |
| A4RMZ0 | 148305 |
| Q2HAE1 | 38033 |
| A6SI42 | 332648 |
| A5DBU3 | 4929 |
| A8NFG5 | 240176 |
| A3LRF8 | 4924 |
| Q5A8H6 | 5476 |
| Q6FWJ9 | 5478 |
| A5E5U5 | 379508 |
| Q6BWZ8 | 4959 |
| Q08225 | 4932 |
| B3LJ02 | 285006 |
| B5VRJ4 | 545124 |
| B8NA87 | 332952 |

| symbol DPP3 | |
|---|---|
| Q9NY33 | 9606 |
| Q5RCB1 | 9601 |
| O55096 | 10116 |
| Q8BU29 | 10090 |
| Q3THP1 | 10090 |
| Q99KK7 | 10090 |
| Q58CS3 | 9913 |
| B5X435 | 8030 |
| Q6DI20 | 7955 |
| Q6PA12 | 8355 |
| Q6DE90 | 8355 |
| B3S0Q5 | 10228 |
| Q298R2 | 46245 |
| B4K999 | 7230 |
| B4G4H0 | 7234 |
| B4LYY4 | 7244 |
| Q17GV2 | 7159 |
| B4JV57 | 7222 |
| B0WDL4 | 7176 |
| Q2XMY9 | 6239 |
| Q9U3K7 | 6239 |
| B3M0E9 | 7217 |
| B4PT52 | 7245 |
| B3P4Y4 | 7220 |
| Q1DZR2 | 5501 |
| B2VQV5 | 426418 |
| B8LTS2 | 441959 |
| Q5JPB8 | 9606 |
| B4DLX4 | 9606 |
| A7RZW4 | 45351 |
| Q9VHR8 | 7227 |
| Q8IGZ7 | 7227 |
| B4QYL5 | 7240 |
| B4HLK9 | 7238 |
| A9VAS0 | 81824 |
| Q5B559 | 162425 |
| Q6CUL1 | 28985 |
| Q756M5 | 33169 |
| B0CSE4 | 486041 |
| A7TI55 | 436907 |
| B6LUF4 | 7739 |
| Q7SH28 | 5141 |
| A6RH25 | 339724 |
| Q0UNF1 | 13684 |
| A6ZNF3 | 307796 |
| B4E357 | 9606 |
| Q3UDD5 | 10090 |
| Q3UDF3 | 10090 |
| Q6NXZ0 | 10090 |
| Q8C0I6 | 10090 |
| Q0CHM4 | 341663 |

| symbol FGF3 | |
|---|---|
| P11487 | 9606 |
| Q8R5L9 | 10116 |
| Q0VG15 | 10090 |
| P05524 | 10090 |
| P48801 | 9031 |
| P36386 | 8355 |
| B7ZPF9 | 8355 |
| Q3KQ53 | 8355 |
| Q66IF7 | 8364 |
| P48802 | 7955 |
| B3DHR1 | 7955 |
| B5X6L5 | 8030 |
| A6H6W8 | 9913 |
| A7SG69 | 45351 |
| A7SI24 | 45351 |
| A8P371 | 6279 |
| A8Q0I7 | 6279 |
| B6KY19 | 7739 |
| B6M7K3 | 7739 |
| O15520 | 9606 |
| O35565 | 10090 |
| O35622 | 10090 |
| O42407 | 9031 |
| O43320 | 9606 |
| O54769 | 10116 |
| O60258 | 9606 |
| O76093 | 9606 |
| O88182 | 10116 |
| O89101 | 10090 |
| O95750 | 9606 |
| P03968 | 9913 |
| P03969 | 9913 |
| P05230 | 9606 |
| P08620 | 9606 |
| P09038 | 9606 |
| P10767 | 9606 |
| P11403 | 10090 |
| P12034 | 9606 |
| P12226 | 8355 |
| P13109 | 10116 |
| P15655 | 10090 |
| P15656 | 10090 |
| P19596 | 9031 |
| P21658 | 10090 |
| P21781 | 9606 |
| P31371 | 9606 |
| P36363 | 10090 |
| P36364 | 10116 |
| P37237 | 10090 |
| P48798 | 13616 |
| P48800 | 9031 |

| symbol FGF3 | |
|---|---|
| P48804 | 9031 |
| P48807 | 10116 |
| P54130 | 10090 |
| P55075 | 9606 |
| P61148 | 10090 |
| P61149 | 10116 |
| P61150 | 10116 |
| P61328 | 9606 |
| P61329 | 10090 |
| P63075 | 10090 |
| P63076 | 10116 |
| P70378 | 10090 |
| P70379 | 10090 |
| P70492 | 10116 |
| P79150 | 9615 |
| Q02195 | 10116 |
| Q11184 | 6239 |
| Q197G3 | 9031 |
| Q20FD0 | 9615 |
| Q28DZ3 | 8364 |
| Q2LGG1 | 7955 |
| Q5KRA4 | 9031 |
| Q5M7N7 | 8364 |
| Q5PRC3 | 7955 |
| Q5TKR1 | 7955 |
| Q641J3 | 8364 |
| Q6DFL4 | 8355 |
| Q6GLR6 | 8355 |
| Q6GLX1 | 8355 |
| Q6NZS6 | 7955 |
| Q6SJP8 | 7955 |
| Q76LI5 | 10116 |
| Q7ZZN4 | 9031 |
| Q8AY90 | 7955 |
| Q8I6J3 | 7719 |
| Q8I6J6 | 7719 |
| Q8R5L5 | 10116 |
| Q8R5L6 | 10116 |
| Q8R5L7 | 10116 |
| Q8R5L8 | 10116 |
| Q8VCY9 | 10090 |
| Q8VI79 | 10116 |
| Q8VI80 | 10116 |
| Q8VI81 | 10116 |
| Q8VI82 | 10116 |
| Q90722 | 9031 |
| Q92913 | 9606 |
| Q92914 | 9606 |
| Q92915 | 9606 |
| Q95L12 | 9823 |
| Q9DDN0 | 9031 |

| symbol FGF3 | |
|---|---|
| Q9EPC2 | 10090 |
| Q9ERW3 | 10116 |
| Q9ESL8 | 10090 |
| Q9ESL9 | 10090 |
| Q9ESS2 | 10090 |
| Q9EST9 | 10116 |
| Q9GZV9 | 9606 |
| Q9HCT0 | 9606 |
| Q9I950 | 9031 |
| Q9IAI3 | 9031 |
| Q9IAI5 | 9031 |
| Q9JJN1 | 10090 |
| Q9NP95 | 9606 |
| Q9NSA1 | 9606 |
| Q9W6A1 | 9031 |
| A0AUQ1 | 7955 |
| A6P7H5 | 8355 |
| A6P7H6 | 8355 |
| A6QPP3 | 9913 |
| A8K1P5 | 9606 |
| B1AK18 | 9606 |
| B2R976 | 9606 |
| B3DHZ8 | 7955 |
| B7U3Y0 | 8364 |
| B7U3Y1 | 8364 |
| B7Z4M7 | 9606 |
| B7Z8N0 | 9606 |
| B8Y4H7 | 8364 |
| B8Y4H8 | 8364 |
| B8Y4H9 | 8364 |
| O89096 | 10090 |
| P70377 | 10090 |
| Q0IHY3 | 8364 |
| Q3UR31 | 10090 |
| Q4RP64 | 99883 |
| Q4RVA8 | 99883 |
| Q5R5C0 | 9601 |
| Q5RDS9 | 9601 |
| Q5TKR2 | 7955 |
| Q5TKR3 | 7955 |
| Q5TKR4 | 7955 |
| Q5U3Q2 | 7955 |
| Q794I6 | 10116 |
| Q9W6A2 | 9031 |
| B5LX82 | 8355 |
| B7ZSQ9 | 8364 |
| Q07G49 | 8364 |
| Q5R2I6 | 13735 |
| Q6RX23 | 9863 |
| Q8QG59 | 8296 |
| Q95K97 | 9541 |

CLUSTERS

| symbol FGF3 | |
|---|---|
| Q9N1B9 | 9940 |
| Q790L8 | 10090 |
| A2RTU5 | 10090 |
| B7ZLG4 | 9606 |
| B7ZMS7 | 10090 |
| Q0VF19 | 10090 |
| Q8C471 | 10090 |
| Q0VCA0 | 9913 |
| Q5SQB2 | 10090 |
| Q5TKR7 | 7955 |
| Q5TKR8 | 7955 |
| A8K147 | 9606 |
| B0CM87 | 9555 |
| B0KW92 | 9483 |
| B1MTE5 | 9523 |
| B2KI41 | 59479 |
| B2R5T0 | 9606 |
| B3DGE3 | 7955 |
| B3EX47 | 42254 |
| B4USY8 | 30611 |
| B7NZB1 | 9986 |
| B7U3X6 | 8364 |
| P34004 | 10036 |
| Q5NVQ3 | 9601 |
| Q6ZWS1 | 10090 |
| Q7M303 | 9940 |
| A4LBB8 | 9606 |
| P20003 | 9940 |
| P78443 | 9606 |
| Q4JH23 | 9940 |
| Q541T2 | 10090 |
| Q8QFR9 | 31033 |
| B7U3X7 | 8364 |
| B7U3X8 | 8364 |
| P48803 | 9913 |
| P48805 | 8355 |
| P48806 | 8355 |
| Q542N0 | 10090 |
| Q9DFC9 | 7955 |
| Q9YH31 | 8316 |
| Q5MFQ8 | 13489 |
| Q5TLE1 | 7955 |
| Q8AXA1 | 7955 |
| Q9YGD8 | 8022 |
| A0MTF4 | 9913 |
| A2AXN8 | 9685 |
| Q8NF90 | 9606 |
| A6Y7S4 | 42716 |
| B7ZPK6 | 8355 |
| Q5FXK5 | 8022 |
| Q90Y92 | 8330 |

| symbol FGF3 | |
|---|---|
| Q925A3 | 10090 |
| B2MVW3 | 9940 |
| P48808 | 9940 |
| Q4KL95 | 8355 |
| Q544I6 | 10090 |
| Q5D0X0 | 9860 |
| Q5RAY8 | 9601 |
| Q8C386 | 10090 |
| Q9N198 | 9823 |
| A8K427 | 9606 |
| A9CSE5 | 8410 |
| B2RPH5 | 9606 |
| B7UCP9 | 8364 |
| B7ZQK4 | 8355 |
| Q059W0 | 10090 |
| Q0KJ02 | 8090 |
| Q0KJ03 | 8090 |
| Q2HXK8 | 7955 |
| Q2LJ24 | 7955 |
| Q38PL0 | 7955 |
| Q3ZFI5 | 42716 |
| Q499I9 | 10090 |
| Q91875 | 8355 |
| Q9PVY1 | 8355 |
| B3DJ36 | 7955 |
| B6EBS1 | 7959 |
| B7TIR5 | 38577 |
| O42278 | 7955 |
| O57341 | 7955 |
| Q0PJT5 | 7994 |
| Q3V279 | 10090 |
| Q4R0X9 | 9031 |
| Q805B2 | 7955 |
| Q80ZL6 | 10090 |
| Q8AXC5 | 8355 |
| Q8MJV6 | 9378 |
| Q9DE51 | 8296 |
| A1A514 | 9606 |
| A1A515 | 9606 |
| A2CF52 | 7955 |
| B8Q2W1 | 42716 |
| Q0E9Y8 | 7955 |
| A4IIV2 | 8364 |
| B1AU20 | 10090 |
| B5X194 | 8030 |
| Q0P4D2 | 7955 |
| Q5E9M0 | 9913 |
| Q5F291 | 10090 |
| Q5R8V8 | 9601 |
| Q9IAI7 | 9031 |
| B7Z1C3 | 9606 |

| symbol FGF3 | |
|---|---|
| Q3KNZ6 | 10090 |
| Q1XG75 | 7955 |
| Q4VBV7 | 7955 |
| Q8I6J1 | 51511 |
| B2RPH4 | 9606 |
| O60371 | 9606 |
| Q8N683 | 9606 |
| Q0VBJ8 | 10090 |
| Q3U1V5 | 10090 |

CLUSTERS

| symbol FGFR2 | |
|---|---|
| A1YYN4 | 10090 |
| A1YYM9 | 10090 |
| A1YYN8 | 10090 |
| A1YYM4 | 10090 |
| A1YYN0 | 10090 |
| Q8MY86 | 6161 |
| Q8MY85 | 6161 |
| B3RWV1 | 10228 |
| A1YYM3 | 10090 |
| A1YYM5 | 10090 |
| A1YYM6 | 10090 |
| A1YYM7 | 10090 |
| A1YYM8 | 10090 |
| A1YYN2 | 10090 |
| A1YYN5 | 10090 |
| A1YYN7 | 10090 |
| A1YYP0 | 10090 |
| A1YYP1 | 10090 |
| A2BEU6 | 7955 |
| A4F228 | 8090 |
| A4F229 | 8090 |
| A4F230 | 8090 |
| A4F231 | 8090 |
| A4F236 | 8090 |
| A4F237 | 8090 |
| A4F238 | 8090 |
| A4F239 | 8090 |
| A4IFL5 | 9913 |
| A4JYQ2 | 7955 |
| B1WB19 | 8364 |
| B3DGB8 | 7955 |
| B3DGT9 | 7955 |
| B4DFC2 | 9606 |
| B4F6W5 | 8364 |
| B7ZR24 | 8355 |
| B7ZRE2 | 8355 |
| B7ZVT9 | 7955 |
| B7ZWP2 | 10090 |
| B9EKY4 | 10090 |
| O42127 | 8355 |
| P11362 | 9606 |
| P16092 | 10090 |
| P18460 | 9031 |
| P21804 | 9031 |
| P22182 | 8355 |
| P22455 | 9606 |
| P22607 | 9606 |
| Q03142 | 10090 |
| Q04589 | 10116 |
| Q1LY25 | 7955 |
| Q28GC1 | 8364 |

| symbol FGFR2 | |
|---|---|
| P21802 | 9606 |
| A1YYN9 | 10090 |
| P21803 | 10090 |
| A1YYN6 | 10090 |
| A5A754 | 9823 |
| P18461 | 9031 |
| Q90749 | 9031 |
| Q9TTZ3 | 9986 |
| Q9QVV7 | 10118 |
| Q91286 | 8319 |
| Q9TT07 | 9615 |
| Q8JG38 | 7955 |
| Q8AYP3 | 7955 |
| Q805B9 | 7955 |
| A4JYI8 | 7955 |
| A4QN31 | 7955 |
| A4IHW8 | 8364 |
| Q6GNP8 | 8355 |
| Q03364 | 8355 |
| Q91147 | 8316 |
| Q91150 | 8316 |
| Q2EKC1 | 7741 |
| Q9PSC9 | 8353 |
| Q9PSD1 | 8353 |
| Q95YM9 | 7729 |
| Q9GSH3 | 7729 |
| B3ND12 | 7220 |
| B3P0E3 | 7220 |
| B4MKJ8 | 7260 |
| B4NB61 | 7260 |
| Q09147 | 7227 |
| Q07407 | 7227 |
| A4V320 | 7227 |
| B4PI01 | 7245 |
| B4PL61 | 7245 |
| B4KV63 | 7230 |
| B4KCE2 | 7230 |
| B4HHC2 | 7238 |
| B4IBB0 | 7238 |
| B6MNZ3 | 7739 |
| Q28J96 | 8364 |
| Q3UPE1 | 10090 |
| Q498D6 | 10116 |
| Q4VBK2 | 7955 |
| Q5R8Q3 | 9601 |
| Q60818 | 10090 |
| Q60830 | 10090 |
| Q61563 | 10090 |
| Q61851 | 10090 |
| Q63709 | 10118 |
| Q63827 | 10116 |

| symbol FGFR2 | |
|---|---|
| Q6DD66 | 8355 |
| Q6GNS5 | 8355 |
| Q7TSI8 | 10090 |
| Q800Z1 | 7955 |
| Q8CFK8 | 10090 |
| Q8CIM9 | 10090 |
| Q90330 | 9091 |
| Q90413 | 7955 |
| Q90Z00 | 7955 |
| Q91287 | 8319 |
| Q91288 | 8319 |
| Q91742 | 8355 |
| Q91743 | 8355 |
| Q91897 | 8355 |
| Q95M13 | 9913 |
| Q99052 | 10090 |
| Q9DGK3 | 8355 |
| Q9I8X3 | 7955 |
| Q9JHX9 | 10116 |
| Q9PS96 | 8355 |
| Q9PSV8 | 8355 |
| Q9PSV9 | 8355 |
| Q9QZM7 | 10090 |
| Q91285 | 8319 |
| Q96KE5 | 9606 |
