# Supplementary figures and images for: Preimplantation development regulatory pathway construction through a text-mining approach
Source: BMC Genomics. 2011 Dec 22;12(Suppl 4):S3. doi: 10.1186/1471-2164-12-S4-S3 (PMC3287586; doi:10.1186/1471-2164-12-S4-S3)

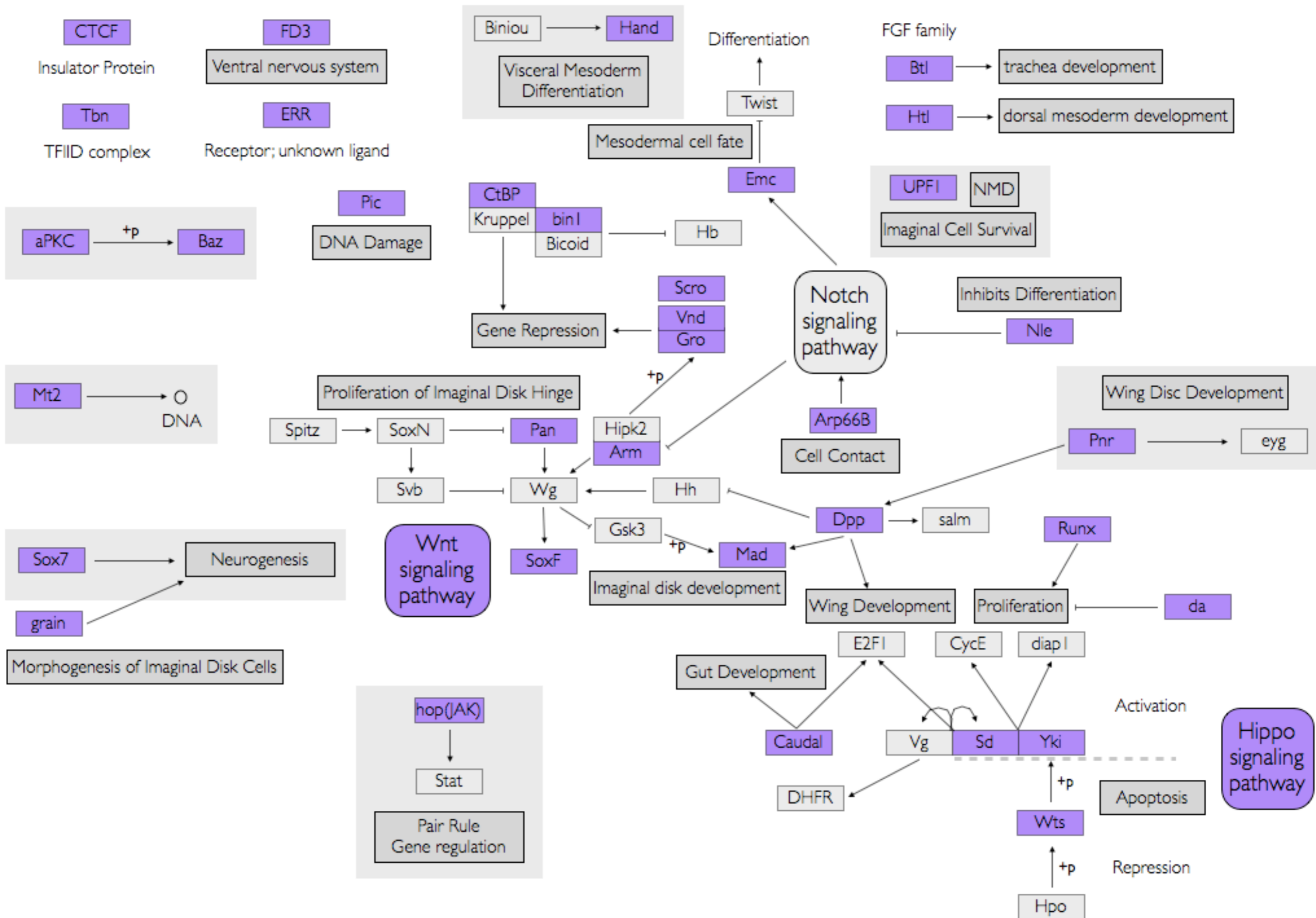

Supplement: Additional file 2 — Ortholog functions in Drosophila melanogaster. This figure represents the corresponding D. melanogaster orthologs found by SeedServer and their respective interactions and functions in fruit fly development. Note that these orthologs are involved in processes related to D. melanogaster embryo development. See Additional file 3 for a table with gene name correspondence between the genes in this figure and the ones on Figure 3. [file 1471-2164-12-S4-S3-S2.pdf]
