# Supplementary material for: Preimplantation development regulatory pathway construction through a text-mining approach
Source: BMC Genomics. 2011 Dec 22;12(Suppl 4):S3. doi: 10.1186/1471-2164-12-S4-S3 (PMC3287586; doi:10.1186/1471-2164-12-S4-S3)
Supplement: Additional file 3 — Gene correspondence table. Human and Drosophila melanogaster gene name correspondence for the orthologs grouped by SeedServer. Column 3 lists the PubMed identifiers (PMIDs) from the papers where functions described in Additional file 2 were found. [file 1471-2164-12-S4-S3-S3.pdf]

Pathway Gene	*D. melanogaster* Gene	Function Reference (PMID)
**BORIS**	CTCF	19210964; 17805343
**PARD3**	Baz	19472188
**RENT1**	Upf1	17196039
**GATA6**	Pnr	12900462; 11731463
**CDX2**	Cad	11846473; 12368268
**SOX7**	Sox100B; Q9va16	19754708
**LATS2**	Wts	16096061; 18328423
**NLE1**	Nle	16785210; 9857191
**DPP3**	DppIII	12846841; 9006064; 8700205
**ATP6AP2**	CG8444	not found
**PKA**	PkaC1; PkaC2; PkaC3	18646611
**TCF3**	Da	12070099; 15024404
**aPKC**	Pkc53e; Wnt6	19472188
**GATA2**	Grn	11044401
**DNMT1**	Mt2	12944428; 15533947
**CtBP**	Rbbp8	19049982
**YAP1**	Yki	16096061; 18331708
**ARP3**	Arp66B	18976918; 19543274
**RPS14**	RpS14a	not found
**CTNNB1**	Arm	19668359; 19232093; 19088090
**SAP18**	Bin1	19049982; 15649476; 16823614
**TLE2**	Gro	17070676; 15802274
**GATA4**	Pnr	12900462; 11731463
**ETIF2B**	Eif2B	12466526; 18313299
**TEAD4**	Sd	18258485; 18313299
**DDB1**	Pic	15514059
**NANOG**	Scro; Vnd	17070676; 10842079
**JAK2**	Hop	11804783; 12441298
**TBN**	Taf8	11438666
**HAND1**	Hand	17904115; 17511863; 16467358
**BMP4**	Dpp	9006064; 9012523
**GATA3**	Grn; GATAc grain	11044401
**ID2**	Emc	11840322; 15128668; 16026970
**RUNX1**	CG34145	19447650; 18758811
**FOXD3**	FD3	1356269
**SOX17**	Sox15; Sox50e	19176582
**ESRRB**	ERR	12767224
**KLF5**	Nucleic acid binding (unnamed)	not found
**FGFR2**	Btl; Htl	12175485; 15084280
**HNF4A**	Hnf4	19254568
**SMAD1**	Mad	19657393; 19896409; 18997322
